# Calpain-2 participates in the process of calpain-1 inactivation

**DOI:** 10.1042/BSR20200552

**Published:** 2020-11-03

**Authors:** Fumiko Shinkai-Ouchi, Mayumi Shindo, Naoko Doi, Shoji Hata, Yasuko Ono

**Affiliations:** 1 Calpain Project, Department of Basic Medical Sciences, Tokyo Metropolitan Institute of Medical Science (TMiMS), 2-1-6 Kamikitazawa, Setagaya-ku, Tokyo 156- 8506, Japan; 2 Center for Basic Technology Research, Tokyo Metropolitan Institute of Medical Science (TMiMS), 2-1-6 Kamikitazawa, Setagaya-ku, Tokyo 156- 8506, Japan

**Keywords:** autolysis, calpains, inhibition mechanism, proteolysis, regulation, substrate specificity

## Abstract

Calpain-1 and calpain-2 are highly structurally similar isoforms of calpain. The calpains, a family of intracellular cysteine proteases, cleave their substrates at specific sites, thus modifying their properties such as function or activity. These isoforms have long been considered to function in a redundant or complementary manner, as they are both ubiquitously expressed and activated in a Ca^2+^- dependent manner. However, studies using isoform-specific knockout and knockdown strategies revealed that each calpain species carries out specific functions *in vivo*. To understand the mechanisms that differentiate calpain-1 and calpain-2, we focused on the efficiency and longevity of each calpain species after activation. Using an *in vitro* proteolysis assay of troponin T in combination with mass spectrometry, we revealed distinctive aspects of each isoform. Proteolysis mediated by calpain-1 was more sustained, lasting as long as several hours, whereas proteolysis mediated by calpain-2 was quickly blunted. Calpain-1 and calpain-2 also differed from each other in their patterns of autolysis. Calpain-2–specific autolysis sites in its PC1 domain are not cleaved by calpain-1, but calpain-2 cuts calpain-1 at the corresponding position. Moreover, at least *in vitro*, calpain-1 and calpain-2 do not perform substrate proteolysis in a synergistic manner. On the contrary, calpain-1 activity is suppressed in the presence of calpain-2, possibly because it is cleaved by the latter protein. These results suggest that calpain-2 functions as a down-regulation of calpain-1, a mechanism that may be applicable to other calpain species as well.

## Introduction

Calpains are a family of intracellular Ca^2+^-dependent cysteine proteases (EC 3.4.22.17) expressed in almost all eukaryotes. The primary function of calpains is limited proteolysis of substrate proteins and modulation of their functions, localization or turnover rates. Defects in individual calpain species induce serious pathogenic states at the cell and organ levels, including neurodegenerative diseases and muscular dystrophy [1]. Among the calpain isoforms, calpain-1 (μ-calpain, C1, CAPN1/CAPNS1) and calpain-2 (m-calpain, C2, CAPN2/CAPNS1) are the best studied, and are consequently referred to as conventional or classical calpains [2]. Until now, the two proteins were thought to share functions because their structures and substrate specificities are quite similar, and both are ubiquitously expressed. There are, however, potentially important differences between them. For example, the required calcium concentration for activation of C1 is two to three orders of magnitude lower than that of C2 [3]. In addition, the results of recent studies utilizing isoform-specific knockout or knockdown have supported the idea that C1 and C2 have isoform-specific physiological functions. Knockout mice for *Capn2*, encoding the catalytic subunit of C2, are embryonic lethal [4,5], whereas *Capn1* knockout mice lacking the catalytic subunit of C1 were initially reported to have almost no defective phenotypes except for platelet dysfunction [6]. Later, a mild form of ataxia due to abnormal cerebellar development was established as a phenotype in *Capn1* knockout mice [7,8]. Another indication of a functional difference between C1 and C2 is that knockdown of their catalytic subunits in neural stem cells revealed that C1 represses neural differentiation, whereas C2 promotes glial differentiation [9]. Furthermore, in fibroblasts, cell motility was altered in C2-knockdown but not C1-knockdown cells [10]. Based on these observations, it is reasonable to revisit the question of whether C1 and C2 possess individual functions for which they cannot reciprocally compensate [11]. Among the issues relevant to such a question, substrate specificity of calpain has the potential to define isoform-specific functions, as the activities of these proteins are manifested through regulation of their substrates’ functions. Previously, we quantitatively analyzed the substrate specificities of C1 and C2 using oligopeptide arrays [12]. The results revealed that these two isoforms have different preference/sensitivity for the sequences flanking the cleavage site. Specifically, the cleavability (*k*_cat_/*K*_m_) of peptides varies over 100-fold, and some peptides are more efficiently cleaved by either C1 or C2. These results, together with those of previous studies, suggest that there is a way to quantitatively discern C1 from C2 [13].

In addition, studies of the structural conversion of calpains upon activation have revealed an interesting difference between C1 and C2 [14–16]. Most significantly, the protease core of C2, which is expected to be produced by activation-induced autolysis, is in an intrinsically inactive state; this inactivation is brought about through the instability of a helix in C2, which induces skewness, and a Trp residue proximal to the active site [14]. An independent biochemical study also demonstrated that the C2 protease core has very low activity [17]. In other words, activation of C2 seems to be accompanied by rapid inactivation caused by autolysis. Interestingly, this scenario was not applicable to C1, for which the protease core exhibits weak but sustained activity. In addition, this form of C1 is not inhibited by calpastatin (CAST), an endogenous inhibitory protein, because it lacks two out of three CAST-binding sites [18]. Considering that many examples have shown that sustained activation of calpain could be detrimental *in vivo*, there must be some regulatory mechanisms for the protease core of C1 to ensure that C1 functions properly.

Hence, in the present study, we sought to comparatively characterize C1 and C2, as well as to elucidate the relevance of their properties to their relationship from the standpoint of substrate proteolysis. First, using cardiac troponin T (TnT) as a protein substrate *in vitro*, we showed that the proteolytic activity of C1 lasts longer than that of C2. Second, although both C1 and C2 undergo autolysis, C2 has a stronger tendency to autolyze at multiple sites, including three unique sites in the PC1 subdomain. The corresponding sites in C1 are also proteolyzed by C2, indicating that the substrate specificity of C2 is involved. The interactions between C1 and C2 have been discussed in regard to activation process that modulates the Ca^2+^ requirement of C2 [19]. In line with this, we found that C1 proteolyzes C2 at the N-terminus of its catalytic subunit, which could lower Ca^2+^ requirement for C2 activation. Our results further suggest that the protease activity of C2 down-regulates the activity of C1. Therefore, C1 and C2 may reciprocally regulate each other, which is not always for mutual reinforcement; instead, C2 plays a role in quenching proteolysis by C1, which would otherwise be prolonged and damaging to the cells.

## Materials and methods

### Materials

Human cardiac TnT (Merck, 648484-100 μg, purity 95%), recombinant human TnT type 2 (Novus Bio, NBC-1-28765-0.1mg), porcine calpain-1 (porcine erythrocyte, 208712-1mg, Merck Millipore) and domain 1 fragment of CAST (CAST-d1) (TaKaRa Bio) were purchased as purified proteins. Recombinant human calpain-2 and proteolytically inactive mutant calpains, calpain-1:CS (C1:CS) and calpain-2:CS (C2:CS), were produced and purified as described previously [20,21]. Specific activity of calpains was defined by casein assay (100–350 U/mg) [22]. Trypsin was obtained from Promega (V5111). The FRET substrate AF-648, LDPMDSTYLE(Edans)ALGIGTIPPK(Dabcyl)YRKLLELNEA, was designed using the sequence of inhibitory domain 1 of rat CAST (Uniprot: P27321) with a two-amino acid deletion (Lys235 and Glu236) and Glu242Lys substitution, and was synthesized by Peptide Institute (Osaka, Japan).

### SDS-PAGE, Western blot and image processing

Proteins were separated by SDS-PAGE and stained with Oriole Fluorescent Gel Stain (Bio-Rad). For better resolution of low molecular weight protein fragments, the gel was run with 2× running buffer [23]. Gels were scanned on an ImageQuant LAS 4000 (GE Healthcare Life Sciences). Quantitation of visualized signals was performed using ImageJ (ver. 1.52k, http://imagej.nih.gov/ij). Images were processed with Adobe Photoshop and Illustrator. For Western blot, proteins separated by SDS-PAGE were transferred onto PVDF membranes (Millipore) and analyzed with the following antibodies: anti- CAPN1 (1:1000, clone 9A4H8D3, C-266, Sigma-Aldrich), anti-CAPN2 (1:1000, clone 1D10A7 and 4B9F) [24], anti-CAPNS1 (1:1000, P-1, ATCC), anti-CAST (1:1000, #4146, Cell Signaling Technology), anti-spectrin alpha chain (nonerythroid, 1:500, cloneAA6, MAB1622, Merck), anti- tubulin (1:2000, clone DM1A, Sigma-Aldrich), anti-μ-post (post autolytic CAPN1) (1:500) [25], and horseradish peroxidase-coupled secondary antibodies (Nichirei Bioscience or Dako). Signals were visualized using Clarity™ Western ECL substrate (Bio-Rad) or a POD immunostaining set (FUJI FILM Wako Pure Chemical). Chemiluminescent signals and digitized membrane images were detected and recorded by ImageQuant LAS 4000 or FUSION FX7.EDGE (VILBER LOURMAT). Uncropped images of the western blots were provided as Supplementary Figures S6–10.

### Proteolysis of Troponin T in vitro

For SDS-PAGE analysis, the indicated amount of TnT was incubated with C1 or C2, separately or in combination, in 50 mM Tris-HCl (pH7.5), 1 mM DTT and 5 mM CaCl_2_ at 30°C. The reaction was terminated by adding SDS-PAGE sample buffer at the indicated time. Degradation of TnT was evaluated by quantitating band intensities as described above.

For LC-MS/MS analysis, 1.5 μg of TnT was digested with 0.2 units of calpains in 5 μl of 80 mM HEPES (pH 7.5), 1 mM tris-(2-carboxyethyl)phosphine (TCEP) and 1 mM (for C1) or 6 mM (for C2) CaCl_2_ for 20 or 40 min at 30°C, followed by addition of methyl methanethiosulfonate (final concentration, 33 mM) to stop the reaction.

### Amino terminal sequencing

The N-terminal amino acid sequence of detected protein fragments was analyzed by APRO Science Inc.

### MS-based analysis of Troponin proteolysis by calpain and autolysis of calpain

For quantitation of TnT peptides generated by calpain, the products were labeled with iTRAQ® reagents (114: C2, 40 min; 115: C2, 20 min; 116: C1, 40 min; 117: C1, 20 min) (Sciex). Samples were then combined and analyzed using the Triple TOF 5600+system (Sciex) equipped with nanoLC Ultra 1D Plus system (Eksigent). In brief, the samples were loaded on the nano cHiPLC Trap column (0.2 mm × 0.5 mm 3-μm ChromXP C18, Eksigent) and analytical column (0.075 mm × 125 mm 3-μm C18, Nikkyo Technos) eluted in a 100-min gradient from 2 to 30% acetonitrile in 0.1% formic acid at flow rate 300 nl/min. MS/MS spectra were acquired with the Analysit TF software (ver.1.5.1, Sciex). The measurement was carried out in triplicate.

Merged data were then processed using ProteinPilot (ver.5. 0. 1, Sciex) using the Paragon algorithm with the following parameters: enzyme, none; database, SwissProt (ver.2015.03.); taxonomy, all. Peptides identified with a false discovery rate (FDR) < 1% were selected for further analysis (Table 1).

**Table 1:** Summary of LC-MS analysis of TnT proteolysis by C1 or C2

Qualification	No.
Spectra	39165
Identified spectra	9255
peptides with FDR < 1%	5082
Distinct peptides	1699
Quantified distinct peptides with FDR < 1% *	436
TnT peptides clear the qualifications above	242
C1 peptides clear the qualifications above	40
C2 peptides clear the qualifications above	38

*: Distinct peptides derived from identified proteins with acceptable modifications. Accepted modification: iTRAQ at N-terminus and/or Lys, oxidation at Met, methylthiolation at Cys, conversion of N-terminal Glu to PyroGlu.

Both the N- and the C-terminal ends of the identified peptides represent cleavage sites by calpain, and these positions referred to the number of peptide bonds of TnT. Peptides derived from calpain were likewise analyzed to profile autolysis coinciding with substrate proteolysis. Preference for cleavage at the *N*^th^ peptide bond was evaluated based on the sum of the intensity (SI) of the iTRAQ signal for the peptides whose sequences started from the *N* + 1^th^ residue or ended at the *N*^th^ amino acid residue. Alternatively, the occurrence rate of the *N*^th^ peptide bond among all identified cleavage sites was used as an index.

### Reciprocal proteolysis between C1 and C2

Three micrograms of C2:CS was incubated with 0.2 μg of C1 or C2 in 10 μl of 80 mM HEPES (pH 7.5), 1 mM TCEP and 1 mM (for C1) or 6 mM (for C2) CaCl_2_ for 20 min at 30°C; this condition corresponds to 4.6 and 6.4 U/μl of C1 and C2, respectively. The reaction was terminated by adding 7% trichloroacetic acid (TCA) to precipitate the relatively large product (>3000 Da). The precipitate was dissolved in SDS-PAGE sample buffer and analyzed by SDS-PAGE to confirm proteolysis. The peptide product recovered from the supernatant of the TCA precipitation was desalted using ZipTip Pipette Tips (Millipore) and subjected to LC-MS/MS analysis with modifications: nLC, EASY-nLC 1200 (Thermo Fisher Scientific); trap column, Acclaim PepMap 100 C18 Trap column (0.075 mm × 10 mm 3-μm, Thermo Fisher Scientific) and analytical column (0.075 mm × 125 mm 3-μm C18, Nikkyo Technos); gradient, 0–36% acetonitrile in 0.1% formic acid in 20 min. To capture the cleavage at the N-terminus of Asn117 and C-terminus of Asn128 in CAPN2 and/or C2:CS, the extracted ion chromatograms (XIC) were created using PeakView for the following two peptide products: 117–128 aa, *m/z* = 683.88 ± 0.02, and 503–517 aa, *m/z* 580.60 ± 0.02. The latter peptide was selected as a control based on the fact that the sequence was identified without redundancy and was not too close to the former in the primary sequence of CAPN2; therefore, little interference with generation or detection of the former was expected.

Inversely, proteolysis of C1:CS by C2 was performed at a molar ratio of 1:2, at a total protein concentration of 0.6 μg/μl, for 20 min at 30°C. The autolytic reaction of C2 without C1:CS under the same conditions was carried out in parallel. Generated products were subject to quantitation using iTRAQ reagent (114: C2 only; 115: C1:CS and C2). The LC-MS/MS data were analyzed with ProteinPilot as described above, except that the taxonomy was set to human. Relative signal intensity (iTRAQ115/iTRAQ114) was further analyzed for the sequences identified in both reactions.

### Fluorescence-based measurement of calpain activity

Hydrolysis of FRET substrate AF-648 or suc-LLVY-AMC (Peptide Institute) was performed in 50 μl of reaction buffer consisting of 2 mU/μl calpains, 100 mM Tris-HCl (pH 7.5), 1 mM DTT, 0.2% CHAPS, 0.1% BSA, and 10 mM CaCl_2_ with 45 μM substrate [26]. The reaction was monitored on an FP8300 spectrofluorophotometer (Jasco Corp.) equipped with a Peltier thermostat cell holder at 30°C for 200 s (10-s interval), with excitation and emission wavelengths of 340 and 490 nm, respectively. Activity was measured at the endpoint or by the initial velocity of the reaction. To assay effect of autolysis, C1 was preincubated in reaction buffer at 30°C for 10 min prior to the addition of substrate. Where indicated, incubation was performed in the presence of active or inactive C2 at a C1:C2 molar ratio of 40:1.

### Cell culture and protein lysate preparation

HCT116, HeLa and HEK293 cells were cultured in Dulbecco’s Modified Eagle’s Medium (08459- 64, Nacalai tesque) supplemented with 10% FBS. Total protein lysate was prepared in TED buffer [10 mM Tris/Cl (pH 7.0), 1 mM EDTA, 1 mM DTT] including 2 mM AEBSF as described previously [27]. For the assay of cellular calpain activities, protein concentration was adjusted to 1 mg/ml, and the lysate was incubated in the presence of 5 mM CaCl_2_ with or without 40 nM of CAST-d1 for an indicated time at 30°C. The reaction was terminated by adding SDS-PAGE sample buffer.

### Real-time quantitative PCR analysis

Total RNA from cultured cells was extracted by TRIzol® reagent (Invitrogen) according to manufacturer’s instruction. First-strand cDNA was synthesized from 0.3 μg of RNA using PrimeScript RT Master Mix (Perfect Real Time, TaKaRa). Real-time qPCR (RT-qPCR) was run in the LightCycler 480 II (Roche) using SYBR Premix EX Taq II (T1i RNase Plus, TaKaRa). Reactions were carried out in duplicate or triplicate and results were normalized according to GAPDH quantitation in the same reaction. The primer sets were listed in Table 2.

**Table 2:** Primer sequences for RT-qPCR

**huCAPN1 FW**	5′-cctcaataggatcatcagcaaa-3′
**huCAPN1 RV**	5′-tcacgatccatgaggttcac-3′
**huCAPN2 FW2**	5′-tcggctttggcatctatga-3′
**huCAPN2 RV2**	5′-gctgaggtggatgttggtct-3′
**huCAPNS1 FW2**	5′-ggtggccgtgatggatag-3′
**huCAPNS1 RV2**	5′-tcggtcagtgtcgaactgttt-3′
**huCAST FW**	5′-aaaccccagcttcaacgac-3′
**huCAST RV**	5′- catcgaggtctttgtcactctg-3′
**GAPDH Fw**	5′-agccacatcgctcagacac-3′
**GAPDH Rv**	5′-gcccaatacgaccaaatcc-3′

### CAPN knockdown by small interfering RNA

Cells were transiently transfected with 20 nM small interfering (si) RNA using Lipofectamine RNAiMAX (Thermo Fischer Scientific) and subsequently cultured for 48 h. The siRNAs for CAPN1 (sc-29885), CAPN2 (sc-41459) and non-targeting siRNA (Control siRNA-A, sc-37007) were purchased from Santa Cruz Biotechnology [28–30]. At 24 and 48 h after transfection, efficiency of knockdown was analyzed by RT-qPCR and Western blot, respectively.

### Structure modeling

Tertiary structures of CysPc domain of C1 and C2 were modeled according to PDB entries 1TL9 and 3BOW, respectively [32,33]. Data were processed in the Molecular Operating Environment (MOE) ver. 2018.01 (Chemical Computing Group and MOLSIS Inc.).

### Sequence alignments of calpain family members

Protein sequence data were retrieved from UniProtKB. CAPN1: P07384 (human), Q9GLG2 (macaque), Q27970 (cow), P35750 (pig), F6UZV8 (dog), P97571 (rat), O35350 (mouse), and CAPN2; P17655 (human), Q9GLG1 (macaque), Q27971 (bovine), I3LQD3 (pig), F1P975 (dog), Q07009 (rat), O08529 (mouse). Sequence alignment was carried out by Genetyx vers. 15 (Genetyx Corp.) or Clustal Omega program (https://www.uniprot.org/align/).

### Cleavage site prediction

Cleavage site prediction was carried out using Calpacchopper (http://calpain.org/predict.rb?cls=substrate), which was constructed based on the result of peptide substrate digestions [12].

### Statistics

Data were analyzed triplicate unless otherwise mentioned, and are expressed as mean ± SEM. By two-tailed *t*-test, values of *P*<0.05 were considered as statistically significant.

## Results

### C1 is a long-lasting protease

We first sought to determine whether a difference in substrate specificity between C1 and C2 is reflected by the efficacy or pattern of substrate proteolysis. For this assay, we used cardiac TnT, a well-established *in vivo* substrate of calpain [34].

**Figure 1:**
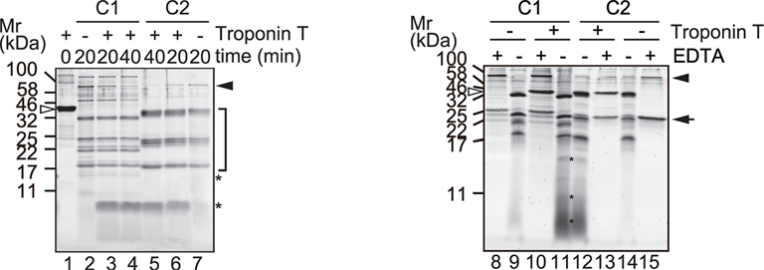
TnT digestion by calpain under exhaustive conditions TnT (1.1 μg) was incubated with 100 mU of calpain-1 (C1) or -2 (C2) in the presence of 5 mM CaCl_2_ at 30°C for the indicated time (left panel) or 20 min (right panel). Lane 1, TnT; lanes 2, 8, 9, C1; lanes 3, 4, 10, 11, TnT and C1; lanes 5, 6, 12, 13, TnT and C2; lane 7,14, 15, C2. For lanes 8, 10, 13 and 15, incubation was carried out with additional 50 mM EDTA. The gel was run with 2× running buffer [23] for better resolution of low molecular weight fragments (right panel). Open and black arrowheads, intact TnT and calpain catalytic subunit, respectively; arrow, calpain small subunit; *, proteolyzed TnT fragments; bracket, autolytic fragments of calpain.

When TnT was incubated with excess C1 or C2 in the presence of Ca^2+^, intact TnT completely disappeared after 20 min (Figure 1, lane 1 vs. 3 and 6, also see Supplementary Figure S1A). Most of the signal originated from autolytic fragments of C1 or C2, whereas proteolyzed TnT was barely detectable as a heterologous fragment smaller than 17 kDa (Figure 1, lanes 2 and 7 vs. 3 and 6, lane 9 and 14 vs. 11 and 12). Based on N-terminal amino acid sequencing, we identified two sequences starting from Met81 and Val85 of TnT as components of these fragments. These amino acid residues are located in the linker sequence between the N-terminal variable region and the tropomyosin binding region. In addition to these sites, which are in the vicinity of C1-mediated cleavage site identified *in vivo* [35], the molecular weights of these fragments implied that proteolysis occurred at the C-terminal region of TnT. No difference between reactions using C1 and C2 was detectable, except in the pattern of autolytic fragments (Figure 1, lane 2 vs. 7, see also Supplementary Figure S2).

**Figure 2:**
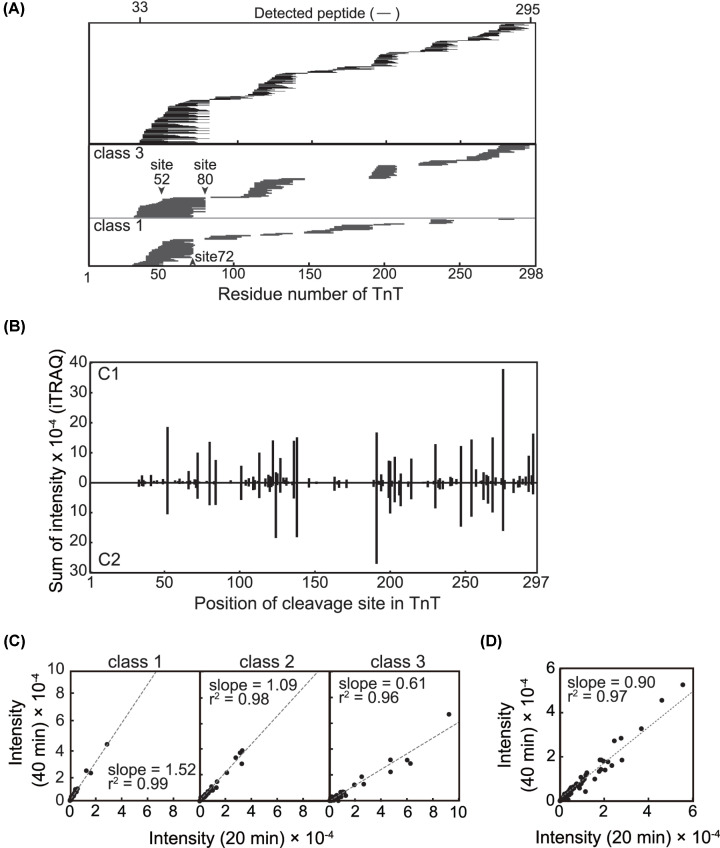
Peptide-based characterization of TnT digestion by calpain (**A**) Peptide map of the TnT**.** (upper panel) TnT was digested by C1 or C2 for 20 min (lanes 3 and 6 in Figure 1) and 40 min (lanes 4 and 5 in Figure 1), labeled with iTRAQ reagents and analyzed by LC-MS/MS (see ‘Materials and methods’ section). The position of 242 distinct peptides of TnT is depicted by horizontal lines. The identified peptide spans 33-295 aa of TnT sequence yielding 88.6 % coverage. Positions of peptides in class 1 (lower panel) and 3 (middle panel) generated by C1 digestion, whose relative intensities increased and decreased between 20 and 40 min, respectively, are represented schematically. Please see the description about the class in legend of (**C**) and (**D**). (**B**) Overview of cleavage site for calpain. Sites of cleavage by calpain are represented by both the N-terminal and C-terminal ends of the identified peptides. As an index of cleavage frequency at each peptide bond, the intensities of the iTRAQ reagent derived from the corresponding peptides were summed (Supplementary Table S1). Sites of cleavage by C1 and C2 did not significantly differ from each other. The relative frequency of each cleavage event shows some difference. Time-dependence of TnT digestion by (C) C1 and (D) C2. For all the distinctive peptides identified, relative intensities acquired after 40-min digestions were plotted against those for the 20-min reaction. Each dot corresponds to an individual peptide. C1 cleavage products were categorized into three classes based on the ratio of their intensities at 40 versus 20 min (*R*_40m/20m_) in order to draw regression lines with correlation factors comparable to those for peptides generated by C2 digestion. Class1, *R*_40m/20m_ > 1.25; class2, 1.25 > *R*_40m/20m_ > 0.8; class 3, *R*_40m/20m_ < 0.8.

Next, using iTRAQ labeling and LC-MS/MS, we quantitatively analyzed the peptide sequences derived from TnT after 20 or 40 min of incubation with C1 or C2. In total, we identified 242 distinctive peptides distributed over the TnT molecule, corresponding to a sequence coverage of 88.6% (Figure 2A, upper panel, Table 1, Supplementary Table S1). PlseCleavage sites were defined as both termini of these distinct peptides, and the SI values of the iTRAQ signal for the corresponding peptides from the 20- and 40-min reactions were used as indices for relative preferences (Figure 2B). The cleavage sites determined for C1 and C2 were both distributed over the TnT molecule in a similar manner. The numbers of sites with an SI value over 5000, i.e*.* the preferred sites, were 73 and 63 for C1 and C2, respectively. In both cases, cleavage at the C-terminus of Phe80 was one of the preferred sites which complements that Met81 was identified at the N-terminus of the protein fragments above detected. It was also noted that, in the rat TnT sequence, this site corresponds to Phe73 proximal to the reported physiological cleavage site, Arg71 [35]. There were other preferred sites, such as the C- terminus of Arg52, which has not been previously reported and whose physiological relevance remains to be determined.

Comparison of the intensity change between the 20- and 40-min reactions for each peptide indicated that proteolysis of TnT follows different time courses for C1 and C2 (Figure 2C,D). When TnT was digested with C1, the quantities of some peptides increased (Figure 2C, left panel) or decreased (Figure 2C, right panel) between 20 and 40 min. On the other hand, generation of TnT peptide by C2 was complete, or blunted, after 20 min (Figure 2D).

Peptides generated by C1 were classified based on the ratio of their intensities at 40 versus 20 min (*R*_40m/20m_): class 1, *R*_40m/20m_ > 1.25; class 2, 1.25 > *R*_40m/20m_ > 0.8; class 3, *R*_40m/20m_ < 0.8 (Table 3). The slopes of the regression lines for the individual classes were 1.52, 1.09, and 0.61. The distribution of peptide sequences in classes 1 and 3 are summarized in Figure 2A (lower and middle panel). It is indicated that class 3 peptides were further processed even after 20 min and some of the resultant shorter peptides were detected as class 1 peptides. For instance, one of the class 1 peptides, 53–72 aa, could be generated from the class 3 peptides 53–80 aa or 42–72 aa between 20 min and 40 min. This set of peptides implies that activity of C1 lasts as long as 40 min or even longer (see Supplementary Figure S1B) under the conditions employed here, and that proteolysis of TnT by C1 takes multiple steps where the initial cleavage primes the next cleavage.

**Table 3:** Relative quantity of TnT peptides generated by C1 or C2

	R_C1/C2_*	Category of peptide generated by C1			
		Increase (Class 1, *R*_40m/20m_ > 1.25)	No change (Class 2, 1.25 > *R*_40m/20m_ > 0.8)	Decrease (Class 3, 0.8 > *R*_40m/20m_)	Sum
**At 20min**	>1.25	37	38	51	126
**At 40min**	>1.25	51	38	28	117
**At 20min**	1.25> >0.8	10	8	13	31
**At 40min**	1.25> >0.8	10	6	18	34
**At 20min**	0.8>	22	21	42	85
**At 40min**	0.8>	8	23	60	91
	Total	69	67	106	242

Relative amount of the TnT peptides generated by C1 or C2 after 20 min and 40 min was evaluated by the ratio of their intensities at each time point. *, R_C1/C2_, the relative intensity iTRAQ117/iTRAQ115 and iTRAQ116/iTRAQ114 for 20 min and 40 min, respectively; R_40m/20m_, the relative intensity iTRAQ116/iTRAQ117.

For the TnT peptides generated by C2, the slope of the regression line was 0.90 (Figure 2D) demonstrating that these peptides were not further proteolyzed. Supplementing C2 after 20 min did not significantly change the quantity of peptide products (Supplementary Figure S1C). Therefore, we assumed that almost all the potential cleavage sites in TnT were completely cleaved by C2 within 20 min.

### Different autolytic profiles of C1 and C2

**Figure 3:**
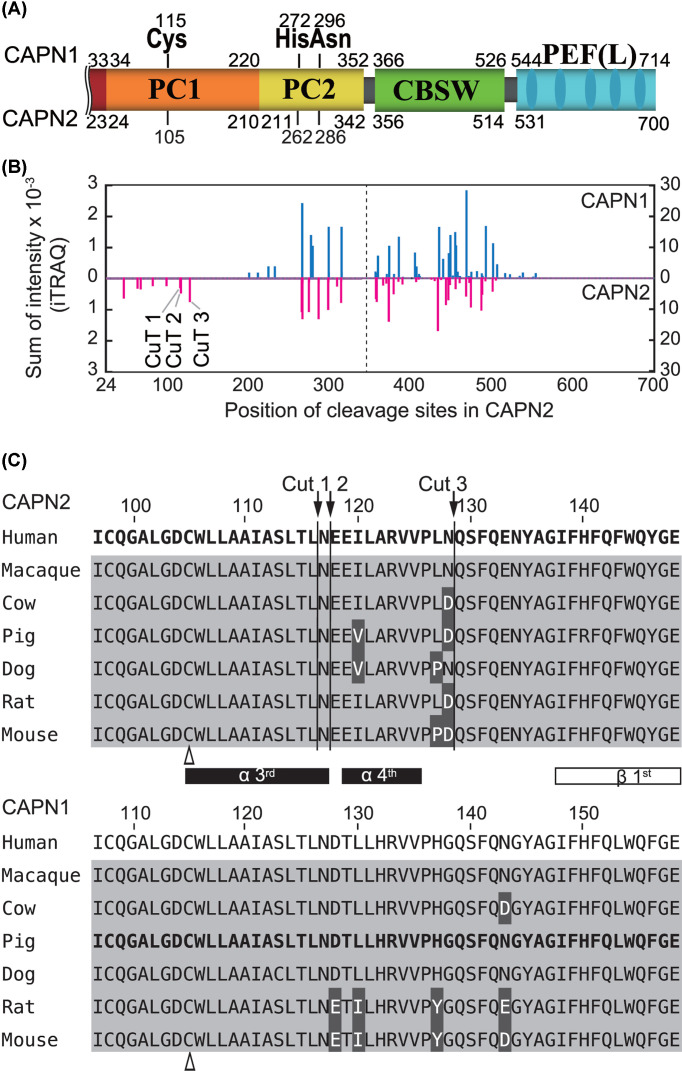
Autolysis of C1 and C2 (**A**) Domain structure of CAPN1 and CAPN2. Due to its extended N-terminal sequence, CAPN1 (catalytic subunit of C1) is 14 amino acids longer than CAPN2 (catalytic subunit of C2). The alignment of the two amino acid sequences was adjusted using the catalytic amino acid residues as indices. (**B**) Comparison of autolytic sites in catalytic subunits, CAPN1 and CAPN2. Using the identified peptide sequence of C1 and C2 in the LC-MS/MS data described in Figure 2, autolytic sites of C1 and C2 are defined and the SI values were calculated as in Figure 2B (Supplementary Tables S2 and S3). C2-specific cleavage sites were identified in the PC1 domain of CAPN2, and were designated CuT1–3 (cleavage site unique to calpain-2 (Two)). Cleavage sites are located C-terminal side of the 116th, 117th and 128th amino acids. In the right half of the panel, i.e*.*, beyond the dashed line, the scale is 10-fold larger than the left. (**C**) Amino acid sequence of mammalian CAPN1 and CAPN2 in PC1 domain. The amino acid sequence covering the P20 position from CuT1 to the P' 20 position from CuT3, 97–148 aa of CAPN2 is aligned with the corresponding sequences from mammalian orthologs (upper panel). The α-helices and β-strand are drawn according to the human ortholog [33]. The corresponding region of CAPN1 is also aligned accordingly (lower panel). Amino acid sequence identities in the selected region are 94.6% and 92.9% for CAPN2 and CAPN1, respectively. Sequences for human CAPN2 and pig CAPN1, the latter of which was actually used in the experiment, are in bold. Amino acid residues identical to and different from human sequence are highlighted in gray and black, respectively. Arrows, CuT1–3; open arrowhead, active site Cys residue.

As often observed in *in vitro* proteolytic assays, calpain undergoes autolysis in parallel with substrate proteolysis. In fact, during TnT digestion, both C1 and C2 autoproteolyzed, and their profiles differed slightly (Figure 1, bracket). To determine whether the autolytic sites were distributed differently in C1 and C2, we reanalyzed the LC-MS/MS data described above. Using the peptide sequences generated after 20- and 40-min reactions, we determined the autolytic sites in CAPN1 and CAPN2 (the catalytic subunits of C1 and C2, respectively) Figure 3A, and estimated cleavability based on the SI values (Figure 3B; Supplementary Tables S2 and S3). Due to material availability, we used CAPN1 (pig) and CAPN2 (human) in the present study. Nevertheless, the overall sequence similarity and identity between these calpains were 91% and 62%, respectively, enabling a comparison of the relative positions of autolytic sites in each molecule. Among autolytic sites unique to CAPN1 or CAPN2, we focused on three cleavage sites unique to CAPN2 in the proximity of catalytic residue Cys105: 116Leu/Asn, 117Asn/Glu and 128Asn/Gln (Figures 3B,C, and 9D,G). These sites were designated as CuT [cleavage unique to calpain-2 (two)] 1, 2 and 3, respectively.

Next, we analyzed autolytic fragments of C1 and C2 detectable by SDS-PAGE (Supplementary Figure S2A). The N- and C-terminal ends of these fragments generated in the course of autolysis revealed that C1 and C2 differed in terms of the complexity of their autolytic products. Comparison of the deduced structures of autolytic fragments from CAPN1 and CAPN2 suggested that C2 undergoes more heterogenous and aggressive proteolysis at consecutive peptide bonds. Although the exact CuT sites were not detected as the N-termini or C-termini of any of the fragments, proteolysis at nearby sites was indicated for several fragments from CAPN1 (Supplementary Figure S2B, arrow in the left panel), as well as CAPN2 (Supplementary Figure S2B, arrows in the right panel).

### Reciprocal proteolysis of C1 and C2

To determine whether autolysis at CuT sites is related to substrate specificity of C2, we analyzed reciprocal proteolysis between C1 and C2.

**Figure 4:**
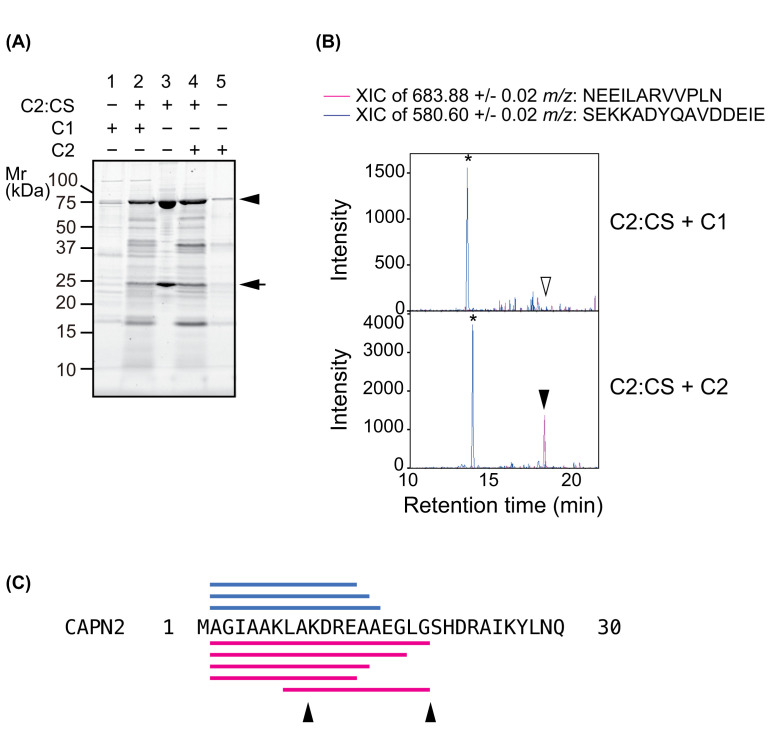
Cleavage in the protease domain of C2 (**A**) Inactive mutant C2:CS as a substrate for C1 and C2. C2:CS was incubated with or without C1 or C2 at a molar ratio of 15:1 for 20 min at 30°C and analyzed by SDS-PAGE. C1 or C2 alone was also subjected to the same analysis (lanes 1 and 5, respectively). For C2:CS, generation of proteolyzed fragments was observed in the presence of C1 (lane 2) or C2 (lane 4), but not in the absence of either (lane 3; ca. 80 kDa and 25 kDa represents CAPN2:CS and CAPNS1, indicated with arrow heads and arrow, respectively). (**B**) Detection of proteolysis at CuT1 and CuT3 in C2. Peptides generated during the proteolysis of C2:CS by C1 and C2 (lanes 2 and 4 in Figure 4A) were identified by LC-MS/MS (Supplementary Tables S4 and S5). An XIC was created for the peptide NEEILARVVPLN (117–128 aa) and SEKKADYQAVDDEIE (503–517 aa), which are located in the PC1 and CBSW, respectively, of CAPN2. Peptide corresponding to cleavage at CuT1 and CuT3 was detected when C2:CS was incubated with C2 (arrowhead), but not with C1 (open arrowhead), whereas proteolysis of C2:CS in CBSW was performed by C1, as well as by C2 (*). (**C**) Identified peptide sequence in the N-terminus of CAPN2. Peptide sequences generated from CAPN2:CS by C1 and C2 are labeled by bars on the top (blue) and bottom (red), respectively. Arrowheads indicate reported autolytic sites in CAPN2; 9Ala/Lys and 19Gly/Ser.

First, we investigated whether C1 cleaves CuT sites in C2 using proteolytically inactive mutant C2:CS as a substrate. In these experiments, C2:CS, expressed and purified as a complex of CAPN2:CS and CAPNS1, was incubated with C1 or C2 and subjected to SDS-PAGE (Figure 4A) and LC-MS analysis (Figure 4B; Supplementary Tables S4 and S5). In the SDS-PAGE analysis, the profile of C2:CS fragments generated by C1 and C2 were mostly similar, with some signals differing between the reactions (Figure 4A, lanes 2 and 4).

LC-MS/MS analysis of the above reactions showed that proteolysis of C2:CS by C1 and C2 was detected with sequence coverage of 15.1% and 37.7%, respectively. Extracted ion chromatograms (XIC) were drawn for two peptides: NEEILARVVPLN (117–128 aa, *m/z* 683.88), which corresponds to the sequence flanked by CuT1 and CuT3; and SEKKADYQAVDDEIE (503–517 aa, *m/z* 580.60), one of the fragments commonly generated by C1 and C2 (Table 4). For *m/z* 683.88, a signal was detectable only when the digestion was performed using C2, whereas the control ion (*m/z* 580.60) was detected in both. Therefore, we concluded that C1 cleaves C2:CS, but not at the CuT sites.

**Table 4:** CAPN2-specific peptide commonly observed after proteolysis of C2:CS by C1 or C2

Sequence	Position (aa)	Intensity (cps)	
		C1	C2
AGIAAKLAKDRE	2–13	2464.38	8548.28
AGIAAKLAKDREA	2–14	214.29	475.89
QDYEALRNECLE	30–41	141.54	158.81
ADPQFIIGGATR	83–94	197.22	945.59
GAEEVESNGSLQK	268–280	1737.13	7915.38
SDTYKKWKLTK	350–360	185.01	1396.76

Abbreviation: cps, counts per second.

On the other hand, C1 cleaves C2 at the N-terminus of its catalytic subunit and two of the fragments are common to those generated by C2 (Figure 4C and Table 4). Since an activation-associated intermolecular autolysis of C2 in this region lowers Ca^2+^ requirement for its activation [36,37], C1 may have a substrate specificity suitable to facilitate the activation of C2 [19].

**Figure 5:**
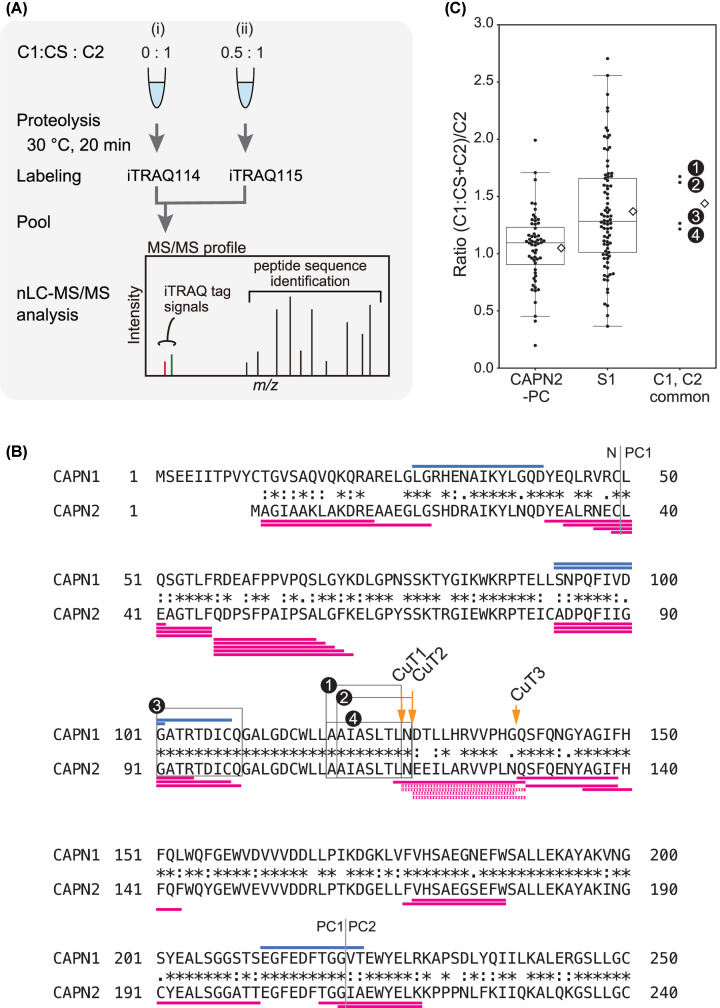
Detection of proteolysis of C1:CS by C2 (**A**) Procedure for the iTRAQ analysis. C2 was incubated alone (i), or together with C1:CS (ii), at the indicated ratios, and generated peptides were labeled with iTRAQ reagent followed by LC-MS/MS quantitation. In theory, when C1:CS is proteolyzed by C2 in a manner comparable to autolysis of C2, the relative intensity iTRAQ115/iTRAQ114 for the peptide sequence present in both C1:CS and C2 is expected to be between 1.5 and 1. (**B**) Identified peptide sequence in the PC1 domain. Among the identified peptides, four sequences from PC1, box 1 and 4, are common to CAPN1 and CAPN2. Sequences unique to CAPN1 and CAPN2 are labeled by bars on the top (blue) and bottom (red, solid and dotted) of the aligned sequences, respectively. Identified peptides are summarized in Supplementary Tables S6–8. (**C**) Quantitation of peptides derived from CysPc domain by inter-isoform proteolysis. Relative intensities for peptides specific to CAPN2 CysPc (CAPN2-PC) or peptides from calpain small subunit (S1), as well as those common to CAPN1 and CAPN2 (C1, C2 common), are summarized as box plots with swarm. Average relative intensities for each set of peptides were 1.1, 1.4 and 1.4, respectively. Peptides specific to CAPN1 theoretically have no intensity value for iTRAQ114, and were therefore omitted. Open diamond, average value; line in the box, median.

Inversely, to determine whether C2 cleaves C1 at a site corresponding to CuT, we incubated a proteolytically inactive C1:CS mutant with C2 at a molar ratio of 1:2, and then quantitatively analyzed the resultant peptides using the iTRAQ reagent (Figure 5A; Supplementary Tables S6, S7 and S8). Cleavage at CuT sites in CAPN2 was detectable by the identification of peptide sequences specific to CAPN2 (Figure 5B, dotted lines in red), whereas the corresponding sequence in CAPN1 was not identified. However, four peptides representing a sequence common to CAPN1 and CAPN2 were identified in the PC1 domain (Figure 5B; box 1, 109–116 aa; box 2, 110-117aa; box 3, 91–99 aa; box 4, 109-117aa in CAPN2), and three of them signifies the cleavage at the CuT1 and CuT2.

We then compared the relative intensities of the peptides common to CAPN1 and CAPN2 with those of the peptides specific to the CysPc domain of CAPN2 and CAPNS1. The rationale for this comparison is that when proteolysis of C1:CS by C2 occurs in a manner similar to autolysis of C2, the relative intensity of the resultant peptide, with a sequence common to CAPN1 and CAPN2, will be as high as 1.5. The average values for the peptides specific to CysPc of CAPN2 and CAPNS1 were 1.0 and 1.4, respectively (Figure 5C). The values for peptides 109–116 aa (box 1 in Figure 5B), 110-117aa (box 2 in Figure 5B), 91–99 aa (box 3 in Figure 5B) and 109-117aa (box 4 in Figure 5B) in CAPN2 increased, to about 1.7, 1.6, 1.3 and 1.2 respectively, in the presence of C1:CS (Figure 5C, C1, C2 common). Therefore, we concluded that C2 proteolyzes C1 as a substrate, and that one of the cleavages occurs between 126 and 127 aa of CAPN1, corresponding to the CuT1 and CuT2.

Another peptide sequence generated from C1 by C2, 28-41 aa in CAPN1, also took our attention (Figure 5B, blue line). In the course of C1 activation, autolysis occurs in the N-terminus of CAPN1, at 15Ser/Ala and 27Gly/Leu producing catalytically active 78 and 76 kDa fragment, respectively. The identified peptide sequence indicates that C2 further trims the N-terminal region of 76 kDa CAPN1 fragment and destabilizes autolyzed form of C1.

### C2 interferes with C1 activity

**Figure 6:**
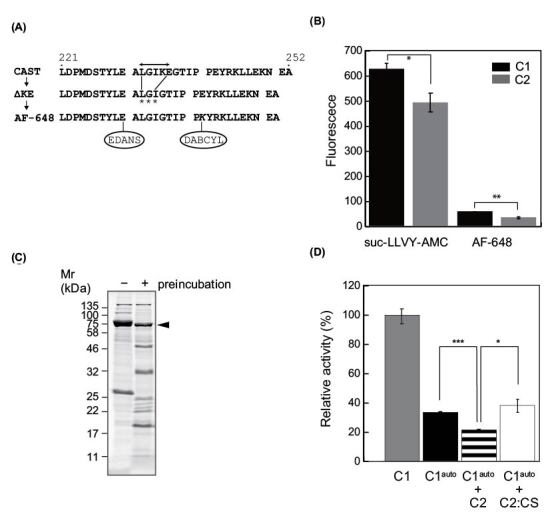
Interference between C1 and C2 (**A**) Modification of the CAST sequence for FRET substrate. In an inhibitory domain B of rat CAST (221-252 of P27321), the sequence GIKEG escapes from the catalytic cleft of calpain (bidirectional arrow). Deletion of two amino acid residues, Lys235 and Glu236, results in proteolysis of CAST within the kink (ΔKE, asterisks) [18]. In a FRET substrate, AF-648, substitution of Glu232Lys (lower, bold italic) were introduced in addition to two amino acid residues (Lys235 and Glu236). (**B**) Activity assay of C1 and C2 using FRET substrates. Utility of AF-648 as a calpain substrate was compared with that of suc-LLVY-AMC by endpoint measurement. The rate of increase in fluorescence of AF-648 in the presence of C1 was 12.7-fold lower than that of suc-LLVY-AMC. Both suc-LLVY-AMC and AF-648 were cleaved more efficiently by C1 than by C2 (1.2-fold and 1.7-fold, respectively). (**C**) C1 after 10 min of autolysis. Preincubation of C1 to induce autolysis resulted in ∼50% reduction in the level of CAPN1 protein, as assessed by signal intensity. (**D**) Inhibitory effect of C2 on C1. Autolyzed C1 (C1^auto^) exhibited a ∼65% decrease in protease activity in the assay using AF-648 (black). When incubation was performed in the presence of C2, an additional ∼40% decrease in the activity toward AF-648 was observed (stripe). Under the same conditions, the protease-inactive mutant C2:CS did not affect the activity of C1. C1 and C2 were mixed at a molar and activity ratio of 40:1 and 30:1, respectively. Activity was measured using the initial velocity of the reaction; *, *P*<0.05; **, *P*<0.01; ***, *P*<0.001.

Next, we asked whether the activity of C1 is affected by C2-mediated proteolysis in addition to autolysis. To measure such interference, it would be convenient to use a substrate more selective for C1. Among the FRET peptide substrates we designed, inspired by a previous structural study showing that deletion mutants at the kink region is proteolyzed by calpain [33], AF-648 was relatively selective for C1. As shown in Figure 6A, AF-648 consists of 30 aa from the inhibitory domain of rat CAST (UniProt ID: P27231, aa 221–252) with modifications as follows: deletion of two central amino acids that allows CAST to escape from the active site of calpain, and the Glu242Lys substitution that permits chemical modification [33].

The sensitivity of AF-648 was much lower than that of the conventional fluorogenic substrate suc- LLVY-AMC for both C1 and C2: the rate of increase in fluorescence of AF-648 using C1 was 12.7- fold lower than that of suc-LLVY-AMC (Figure 6B). However, AF-648 was cleaved 1.7-fold more efficiently by C1 than C2 versus 1.2-fold in the case of suc-LLVY-AMC. Therefore, we evaluated the interfering action of C2 on C1 using AF-648 as a “semi-C1-specific” substrate.

To induce autolysis, C1 was incubated in the presence of Ca^2+^ for 10 min prior to the activity assay with AF-648. This preincubation resulted in a ∼65% decrease in activity (Figure 6C,D, gray and black). When C2 was added to the preincubation at a C1:C2 molar ratio of 40:1, an additional ∼40% decrease in activity was observed (Figure 6D, stripe). This decrease was dependent on protease activity of C2, as preincubation of C1 with C2:CS at the same concentration did not cause any effect (Figure 6D, white). These results demonstrated that, when coactivated, C2 has the potential to proteolyze C1 and counteracts substrate proteolysis by C1. C1-dependent activation of C2 was not detected in this assay condition where 5 mM CaCl_2_ was present.

**Figure 7:**
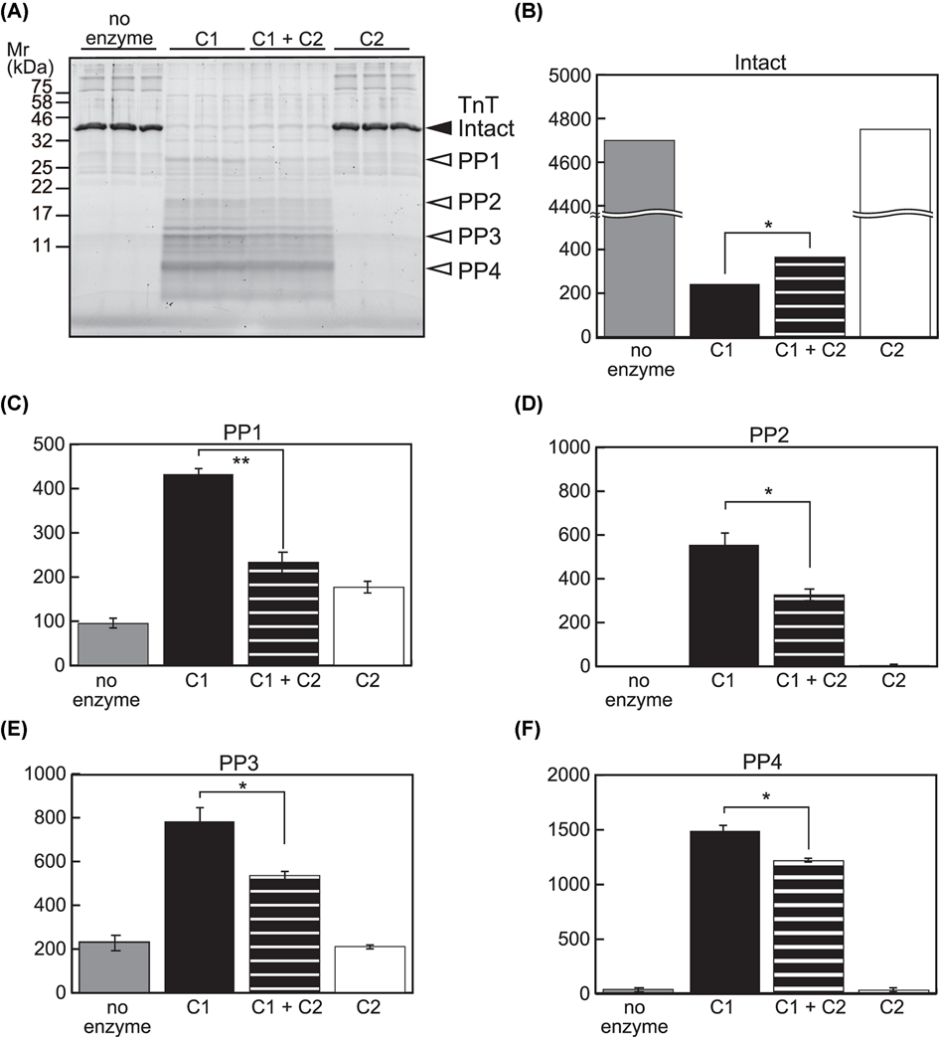
No synergy between C1 and C2 in TnT proteolysis (**A**) Proteolysis of TnT by C1 and/or C2. One microgram of TnT was incubated with C1 (6 mU), C2 (1.5 mU), or both for 20 min, followed by SDS-PAGE analysis. Reactions were performed by triplicate. Average intensities of the full-length (intact) and representative proteolyzed products of TnT (PP1 to 4) were quantitated. (**B–F**) Inhibitory effect of C2 on TnT proteolysis by C1. Proteolysis of TnT protein by C1 was suppressed in the presence of C2, as measured by the decrease in the level of intact TnT (B), as well as the amount of proteolyzed product (C–F). At the amount used, proteolysis of TnT by C2 was barely detectable (A, three lanes from the right); *, *P*<0.05; **, *P*<0.01.

Next, we examined the potential of C2 to interfere with C1 using a protein substrate, TnT. When TnT was incubated with a small amount of C1 (substrate:enzyme = 23:1; a condition far less exhaustive than the one used in Figures 1 and 2) for 20 min, proteolysis of TnT was captured as a decrease in the level of intact TnT and generation of multiple proteolyzed products (Figure 7A–F, no enzyme vs. C1). Considering that the autolysis of C1 were detected as fragment patterns (Figure 1, lane 2) different from the band pattern in Figure 7A and that the amount of C1 used was expected to be below detection, it was reasoned that these products were generated from TnT (Figure 7A, PP1-4). When C2 was included in the reaction, there was a decrease in the amount of proteolyzed product (Figure 7C–F, C1 vs. C1+C2) as well as an increase in the remnant of intact TnT (Figure 7B, C1 vs. C1+C2). Proteolysis of TnT by C2 at the amount used here was negligible (Figure 7A–F, C2). These observations agree with the results obtained using peptide substrate AF-648, and suggest that C2 has a negative effect on C1-mediated proteolysis of protein substrates such as TnT.

### C2 interferes with activation-associated autolysis of C1

We further examined if inhibitory effect of C2 on C1 could be detected under more heterogeneous conditions, for example, in lysates from cultured cells. For this purpose, we compared the expression levels of CAPN1, CAPN2, CAPNS1 and CAST in different cell lines and chose human HCT116 colon cancer cells (Supplementary Figure S3A). HCT116 have increased levels of CAPN2 in comparison with other colon cancer cell lines [38], and we also confirmed that protein levels of CAPN1 and CAPN2 are significantly higher than those in HeLa or HEK293 cells while the amount of CAST is moderate (Supplementary Figure S3B). Difference of calpain enzymatic activities in these cell lines was discernible as proteolysis of spectrin and autolysis of CAPN1 (Supplementary Figure S3C,D). In contrast with C2, activation-associated autolysis of C1 is detectable as generation of 78 kDa and 76 kDa fragments of CAPN1, both of which are functional as catalytic subunit (Supplementary Figure S3C, gray and open arrowhead, respectively) [39].

**Figure 8:**
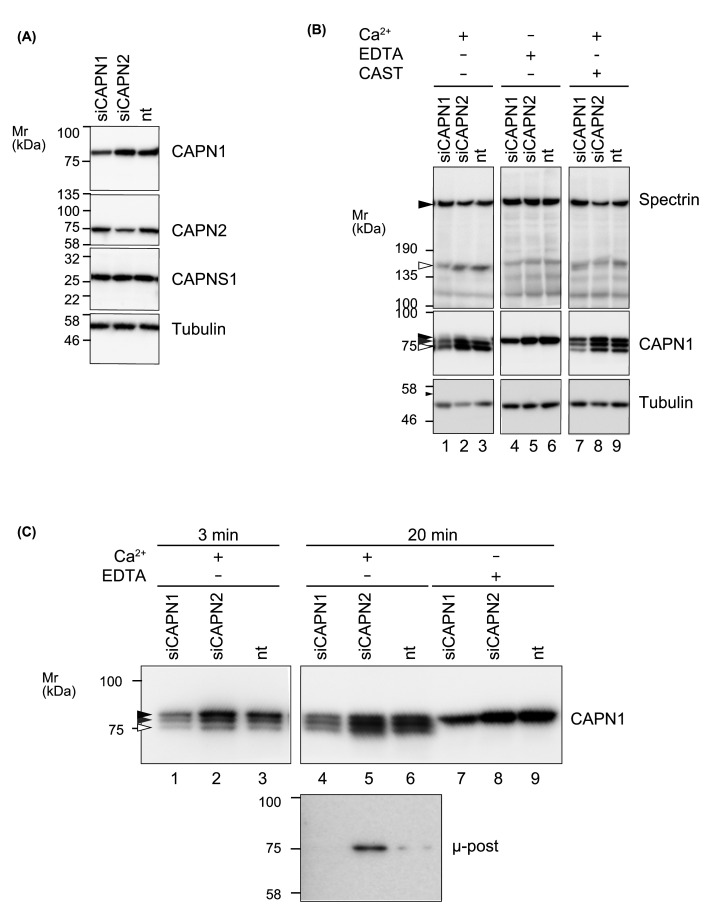
Modification of sequential autolysis of C1 in cultured cells (**A**) Isoform specific knockdown of calpains. HCT116 cells were transfected with siRNA targeting CAPN1 or CAPN2, or non-targeting siRNA (nt). After 48 h, expression levels of indicated proteins were analyzed by Western blot to confirm the efficiency and specificity of knockdown. Each lane contains 20 μg of proteins and tubulin was used as a loading control. (**B**) Activation-associated autolysis of C1. Lysates of siRNA-treated cells were treated with 5mM CaCl_2_ at 30°C for 20 min. Autolysis of C1 was detected as generation of two fragments, 78 kDa (gray arrowhead) and 76 kDa (open arrowhead), using anti-CAPN1 antibody. Arrowhead indicates pre-autolytic, full-length CAPN1 (80 kDa). When C2 was down-regulated by siRNA targeting CAPN2, attenuation of autolysis of C1 was detectable (lane 2 vs. 3, gray and open arrowheads). In the presence of 40 nM CAST-d1, autolysis of CAPN1 as well as proteolysis of spectrin was partially suppressed. Each lane contains 20 μg of proteins. (**C**) Effect of C2 on activation-associated autolysis of C1. Autolytic profiles of CAPN1 were further analyzed by performing another set of incubation using lysates from cells treated with indicated siRNAs. Down-regulation of C2 attenuated the decrease of pre-autolytic fragment of CAPN1 at 3 min (lane 2 vs. 3, arrowhead) and 76 kDa post-autolytic fragment at 20 min (lane 5 vs. 6, μ-post). Lanes contain 8 μg (1–3) and 16 μg (4–9) of proteins.

To evaluate the role of C2 on down-regulation of C1, CAPN1 or CAPN2 was knocked down. Analysis of mRNA levels by RT-qPCR confirmed the specificity of treatment and denied the possible cross-isoform compensation at the transcript level (Supplementary Figure S4). At the protein level, specific decrease of CAPN1 or CAPN2 was confirmed (Figure 8A). Although it was observed that proteolysis of spectrin was less efficient in CAPN1 knocked down cells, no significant enhancement was achieved by knocking down CAPN2 (Figure 8B, lanes 1–3).

Alternatively, in CAPN2 knocked down cells, we observed a slight increase of 78 and 76 kDa fragments of CAPN1 (Figure 8B, lane 2 vs. 3) after 20 min of incubation with 5 mM Ca^2+^. Increased abundance of the 76 kDa fragment in CAPN2 knocked down cells was also confirmed using an antibody specific to its N-terminal sequence (Figure 8C, lane 5). This observation is consistent with that one of the C1 derived peptide sequences generated by C2 matches the N-terminus of 76 kDa CAPN1 fragment (Figure 5B) Addition of CAST in the reaction partially attenuated the sequential autolysis, however, the same trend was maintained (Figure 8B, lane 8 vs 9). When the reaction is terminated earlier, pre-autolytic, full-length CAPN1 was most abundant in CAPN2 knocked down cells (Figure 8C, lane 2). These results indicate that C2 recognizes C1 as a substrate in the cell lysate and enhances a decay of C1.

## Discussion

Here, we uncovered the difference between C1 and C2 in terms of their duration of activity and propensity for autolysis-mediated down-regulation. The protease activity of C1 is sustained for longer than that of C2, and tolerates the effect of autolysis. In the presence of C2, however, the activity of C1 is reduced due to proteolysis by C2. These results suggest that reciprocal cooperativity between C1 and C2 is rare, except for C1-mediated activation of C2, and that this interference maintains the integrity of calpain function by avoiding unnecessary sustained proteolysis, resulting in cellular damages.

### Time-dependent comparison of *in vitro* TnT proteolysis by C1 and C2

TnT is an *in vivo* substrate of calpain. Its affinity for thin filament components is increased by calpain-mediated N-terminal cleavage, after which it modifies the function of cardiac myofilament [35,40,41]. Although C1, rather than C2, has been implicated in the proteolysis of TnT [34], we observed that C2 could also cleave the peptide sequence from TnT as efficiently as C1 [12]. In addition, the cleavage sites in TnT identified in the present study, at the protein and peptide levels, were proximal to yet different from the previously reported sites [35]. Hence, we reasoned that there are theoretically cleavable sites for calpain in protein substrates that have simply not yet been reported, and that the calpain-mediated “limited” proteolysis observed *in vivo* is likely an effect of constraints imposed by secondary and/or higher order structures.

The results shown in Figures 1 and 2 are consistent with this notion. First, TnT was exhaustively but not randomly cleaved by both C1 and C2 *in vitro*, apparently in the same manner. Second, by time-dependent quantitation, we detected a hierarchical framework of the TnT proteolytic reaction (Figure 2A,C). It is anticipated that there is a transition in preferred proteolytic sites due to structural changes in substrates during the proteolytic reaction; cleavage at some sites (first attack) uncovers other cleavage sites (second attack).

Time-dependent quantitation of the TnT proteolysis also revealed a difference between C1 and C2 in regard to duration of activity. In contrast with C1, under the conditions used here, TnT proteolysis by C2 stalled after a 20-min reaction (Figure 2D), although this could be rescued to a limited degree by addition of supplementary of C2 (Supplementary Figure S1C). Therefore, in addition to showing that C1 is superior to C2 in recognizing TnT as a substrate [34], our findings predict that the loss of C2 activity advances in parallel with TnT proteolysis. The most likely mechanism underlying this phenomenon is activation-induced autolysis, and it could be that C2 autocatalytically down-regulates itself more rapidly than C1 does.

### Autolytic site specificity of C1 and C2

The protease core domain of C1, but not C2, has reasonable activity due to conformational stability when it is isolated from the full-length protein by autolysis and/or recombinant expression [14]. Previous studies using rat calpain sequences revealed the importance of Ala213 and Gly203 in CAPN1 and CAPN2, respectively, for differentiating the stability of C1 and C2 after autolysis. On the other hand, due to experimental constraints, we used pig C1 and human C2, both of which have Gly at the corresponding positions. Considering that human and pig have 98–99% similarity at the amino acid sequence level, our results indicate that human C1 and C2 differ from each other in a manner similar to rat C1 and C2. Therefore, we looked for the autolytic site specificities of C1 and C2 in the peptides sequence generated from CAPN1 and CAPN2, catalytic subunits of C1 and C2, respectively, in the course of TnT proteolysis for 20 min. These peptides, which are readily measurable by mass spectrometry, are likely to represent the end products of exhaustive autolysis, whereas the proteolyzed fragment detectable by SDS-PAGE could represent a mixture of the end and intermediate products.

As previously reported for C2 [15], the CBSW domain of CAPN1, the catalytic subunit of C1, is also predominantly proteolyzed. On the other hand, for about half of the autolytic sites identified in CAPN1 and CAPN2, the relative positions within the molecule are unique (26 unique sites out of 47 determined sites, for both). By focusing on autolysis in the proximity of active-site Cys residues, we revealed that the substrate specificities of CAPN1 and CAPN2 are different. In particular, we found that some autolytic sites, such, as CuT1 through CuT3 in CAPN2, accelerate the decay of activity through autolysis.

**Figure 9:**
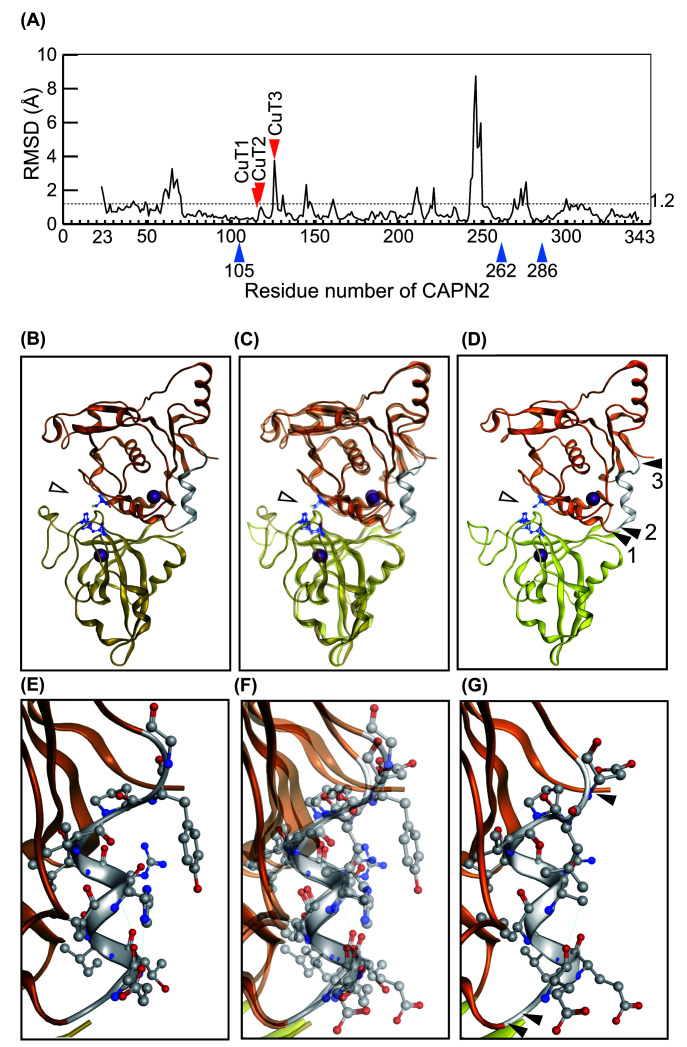
Spatial representation of CuT sites (**A**) RMSD value for CysPc domains of C1 and C2. The average RMSD value for the entire CysPc domain of C1 (PDB ID: 1TL9) and C2 (PDB ID: 3BOW) is 1.2 Å (dotted line). In the proximity of the CuT3 site, the RMSD value is relatively high. Arrowhead, positions of active-site amino acid residues; arrow, autolytic sites identified in CAPN2, CuT1–3. (**B–D**) Comparison of the CysPc domain structure of C1 and C2. The CysPc domains of C1 (B) and C2 (D) are also shown as merged images (C). (D) Autolytic sites in CAPN2, CuT1–3, are located in the region flanking the fourth helix [42]. Arrowheads 1–3, positions of CuT1–3. Open arrowheads, catalytic amino acid residues (See also Figure 3C). (**E**–**G**) Enlarged view of CuT-containing region from B to D. The overall structures of these two molecules are well aligned, reflecting the result shown in Figure 3A.

In the Ca^2+^-bound active conformation, the protease domain of C1 and C2 are well superimposed, with an average RMSD value of 1.22 Å (Figure 9A,C). Because the CuT sites are located at the surface of PC1 domain flanking the helical region [the fourth α-helix in [42]] (Figure 9D), it is likely that cleavages at CuT sites are intermolecular reactions. It is tempting to speculate that cleavage at these sites divides the catalytic domain and breaks a catalytic triad.

For the CuT3 site, local RMSD values were relatively high (Figure 9A, 3.76 and 2.24 Å), which was visible as a slight divergence between C1 and C2 at the C-terminus of helix 7 (Figure 9C). Interestingly, CuT3 was also recognized by a calpain cleavage site predictor (Calpacchopper) as a potential calpain cleavage site in CAPN2, but not in other human CAPNs except for CAPN14 (see ‘Materials and Methods’ section; Supplementary Figure S5). This can be explained by the facts that the probability scores calculated by Calpacchopper depends on the scores of amino acid descriptors at positions P6, P2 and P1 [12], and that the amino acids at P1 and P2 of CuT3 vary among calpain isoforms. Furthermore, this variation is present in other mammalian CAPN2, but only the probability scores of human and macaque CAPN2 were over the threshold. These observations indicate the importance of CuT1 and CuT2, although the predictor does have limitations.

The Tyr137 residue at P2 of CuT3 in CAPN1 protrudes outwards (Figure 9E–G), and occupies a larger space than Leu127 at the corresponding position in CAPN2. Although in the present study we used commercially available pig C1, in which the Tyr137 is substituted by His, based on the result of reciprocal proteolysis assay we concluded that CuT3 is a unique autolytic site in CAPN2 that matches the substrate specificity of C2, but not C1.

On the other hand, CuT1 and CuT2 are theoretically predicted to be incompetent and competent, respectively, as cleavage sites in both CAPN1 and CAPN2 (Supplementary Figure S5B). The prediction is in part contradictory to the experimental result, where C2 recognized CuT1 in the sequence of CAPN1 as well as CAPN2. It is possible that additional parameters that were logically missing in the prediction algorithm reflect autolytic site specificity, e.g*.*, structural changes upon Ca^2+^ binding that cause CuT1 and CuT2 to be more exposed at the surface, as shown previously [43].

### Reciprocal action between C1 and C2

**Figure 10:**
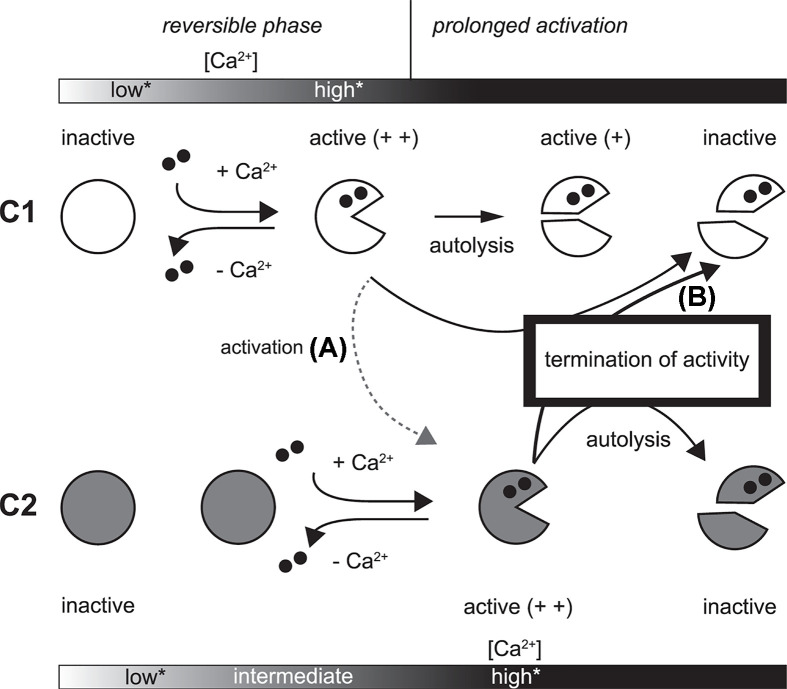
Reciprocal regulation of activity of calpain Differences in the state of the CysPc domains in C1 and C2 as a consequence of activation and inactivation are shown. When sustained activation is caused by persistence of sufficient Ca2^+^ concentration for activation, both C1 and C2 undergo autolysis, the products of which include the isolated protease core (upper part of pie in C1 and C2), which retains some activity. On the other hand, the isolated protease core of C2 is inactive due to an intrinsic structural change. As a result of this difference, activated C1 persists longer in vitro than C2. *, “low” and “high”, respectively, indicate insufficient and sufficient concentration of Ca^2+^ for activation of C1 and C2. “intermediate”indicate a concentration Ca^2+^ sufficient for C1 but insufficient for C2. When C1 is activated at an intermediate Ca^2+^ concentration in close proximity to C2, limited N-terminal proteolysis of C2 by C1 may lower the Ca^2+^ requirement for C2 activation (arrow A). Then, both C1 and C2 are activated and, if the increase in Ca^2+^ concentration is sustained, C2 may target the PC1 domain of C1 as well as itself. In this case, sustained activation of C1 is not achieved, and total proteolytic activity could be promptly down-regulated (arrow B).

Consistent with their high similarity, C1 and C2 exhibit reciprocal proteolytic activity toward each other. At the level of cleavage site preference, however, the reciprocal proteolysis was not identical to autolysis by each protein. This difference led us to examine the impact of reciprocal action between C1 and C2, in particular, the possibility that the long-lasting activity of C1 was shortened in the presence of C2 (Figure 10, arrow B). In addition to CysPc domain, the N-terminal region of CAPN1 is targeted by C2, which likely disturbs the CysPc domain structure [43]. On the other hand, a previous study showed that the Ca^2+^ requirement of C2 was reduced in the presence of C1 under a low Ca^2+^ concentration at which C1 was activated and digested the small regulatory subunit of C2, proposing the calpain cascade (Figure 10, arrow A) [19]. We identified some peptide sequences by our reciprocal proteolysis assay suggesting that intermolecular cleavage of C2 by C1 generates C2 moieties that are different from those generated by genuine autolysis but similar enough to acquire lower Ca^2+^ requirement for activation [44]. Therefore, reciprocal action between C1 and C2 may be involved in both the initiation and termination of calpain activation.

Under two different *in vitro* conditions, we showed that the coexistence of C1 and C2 does not necessarily increase net proteolytic activity, but rather results in reduced substrate proteolysis. The substrates used in these assays are technically cleavable by both C1 and C2, but are preferentially cleaved by C1. Therefore, these results suggest that C1 undergoes pseudo-autolysis by C2, which down-regulates the activity of C1 more rapidly than genuine autolysis by C1 itself.

As an indication of physiological significance of reciprocal regulation between C1 and C2, we focused on that relative expression levels of CAPN1, CAPN2, CAPNS1 and CAST vary among cell lines. For example, altered expression levels of CAPN1 or CAPN2 have been related to pathogenic phenotypes such as malignancy potency [38,45]. In these cases, however, the phenomenon is often explored to identify which calpain, C1 or C2, is the preferential isoform involved in the proteolysis of defined substrates. Rather, we aim to investigate the impact of altered balance between C1 and C2. Sicne C2 requires a higher concentration of Ca^2+^ for activation than C1 does, it might be reasonable that activation of C2 is more tightly regulated in terms of occurrence and duration than that of C1. In addition, sustained activity of C1 and its autolytic product should be appropriately terminated. This hypothesis is consistent with the result that C2-specific knockdown induced an increase in C1 activity in C2C12 cells [46]. Cleavability predictions suggested that some human CAPNs are comparable to CAPN2 as substrates for calpain (Supplementary Figure S5), raising the question of whether reciprocal interference between C1 and C2 are extendable to these calpain family members.

Our results suggest that in a cellular context in which activation of C2 is enabled, C2 is a candidate quencher of C1. Further, using the cell lysates, it was indicated that the ratio of CAPN1/CAPN2 has some impact on the longevity of activated C1. Our model needs further verification, and identification of a condition where C1 and C2 colocalize to realize reciprocal regulation through inter-isoform proteolysis *in vivo* may be critical.

## Supplementary Material

Supplementary Figures S1-S10 and Tables S1-S8

## Data Availability

The mass spectrometry proteomics data have been deposited to the ProteomeXchange Consortium (http://proteomecentral.proteomexchange.org) via the jPOSTrepo [31] with the dataset identifier ProteomeXchange: PXD017015.

## Abbreviations

aa: amino acid residue
C1: calpain-1 (CAPN1/CAPNS1, μ-calpain)
C2: calpain-2 (CAPN2/CAPNS1, m-calpain)
CAPN1: calpain catalytic subunit 1
CAPN2: calpain catalytic subunit 2
CAPNS1: calpain small subunit 1
CBSW: calpain-type β-sandwich domain
CuT: Cleavage unique to calpain-2 (Two)
iTRAQ: isobaric tag for relative and absolute quantitation
*k*_cat_: catalytic reaction rate constant
*K*_m_: Michaelis-Menten constant
*m/z*: mass to charge ratio
min: minutes
MS: mass spectrometry
nLC: nano-scale LC
PC1: protease core 1
PC2: protease core 2
PEF: penta EF-hand
PP: proteolyzed product
RMSD: root mean square deviation
RT-qPCR: real-time quantitative PCR
suc-LLVY-AMC: Succinyl-L-leucyl- L-leucyl-L-valyl-L-tyrosine 4-methylcoumaryl- 7-amide
TCA: trichloroacetic acid
TCEP: tris-(2-carboxyethyl)phosphine
TnT: Troponin T
XIC: extracted ion chromatogram
