# Supplementary material for: Calpain-2 participates in the process of calpain-1 inactivation
Source: Biosci Rep. 2020 Nov 3;40(11):BSR20200552. doi: 10.1042/BSR20200552 (PMC7610153; doi:10.1042/BSR20200552)
Supplement: Supplementary Figures S1-S10 and Tables S1-S8 [file BSR-2020-0552_supp.pdf]

Supplementary Figures

Figure S1. Sustained proteolytic reaction by C1 but not by C2.

Figure S2. Summary of autolytic fragments from C1 and C2.

FFigure S3. Expression of calpain related molecules in different cell lines.

Figure S4. Downregulation of CAPN1 and CAPN2 in HCT116 cells.

Figure S5. Cleavability prediction of human calpain PC1 domains.

Figure S6. Uncropped images of the western blots presented in the Fig. S3B.

Figure S7. Uncropped images of the western blots presented in the Fig. S3C.

Figure S8. Uncropped images of the western blots presented in the Fig. 8A

Figure S9. Uncropped images of the western blots presented in the Fig. 8B.

Figure S10. Uncropped images of the western blots presented in the Fig. 8C.

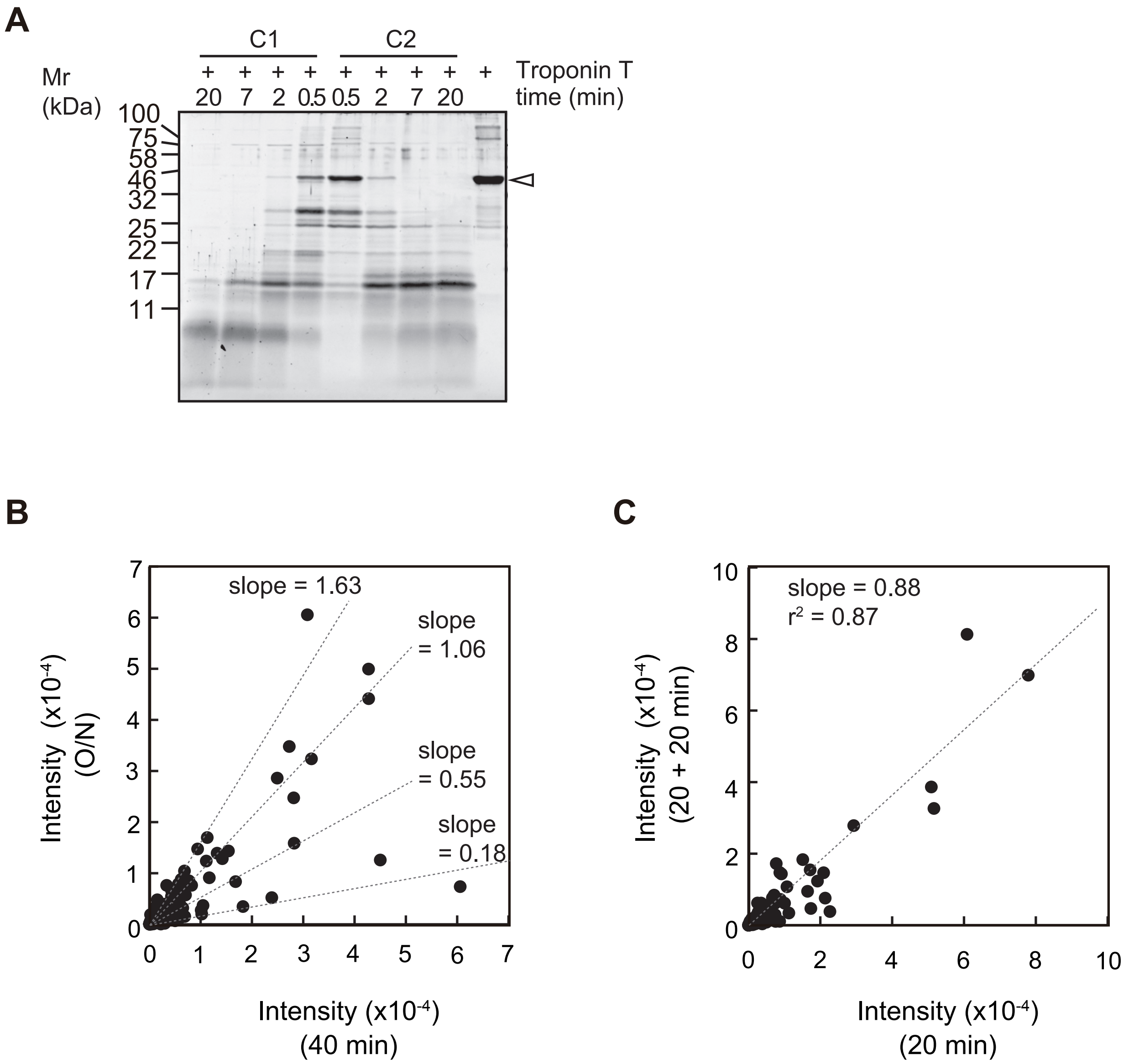

Figure S1. Sustained proteolytic reaction by C1 but not by C2.
(A) One microgram of TnT was incubated with C1 or C2 in the presence of Ca^{2+} for an indicated time. The amount of calpain was 1/50 of that used in Fig. 1A. Open arrowhead, intact TnT.
(B) Digestion of TnT by C1 was carried out overnight, and the products were subjected to LC-MS/MS analysis. Each dot represents a distinct peptide. When compared to the result after 40 min of digestion, the intensities of some peptides decreased or increased over time, suggesting sustained proteolytic activity of C1. The cleavage products were categorized into four groups, and regression lines were drawn. The categories, based on the ratio of intensities after an overnight reaction vs. a 40-min reaction, were as follows: > 1.25; 0.8-1.25; 0.33-0.8; and < 0.33.
(C) After 20 min of digestion of TnT with 0.16 units of C2, the same amount of C2 was added. After an additional 20 min of reaction, no significant change in intensity was observed, suggesting rapid completion of TnT proteolysis by C2.

Shinkai-Ouchi et al, Figure S2

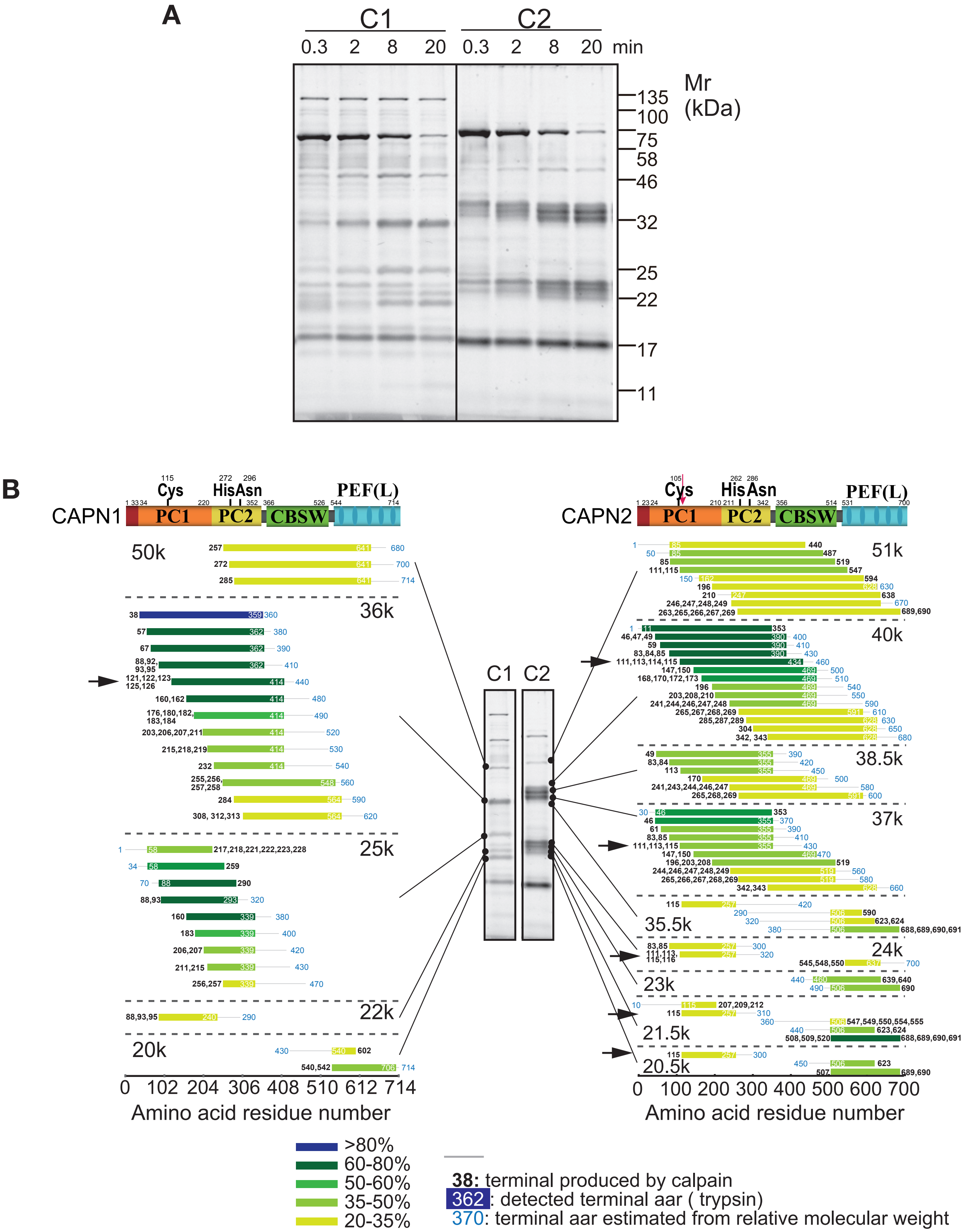

Figure S2. Summary of autolytic fragments from C1 and C2.
(A) C1 or C2 was incubated in 80mM HEPE(pH7.5), 1mM TCEP, and 1mM (for C1) or 6mM (for C2) $CaCl_2$ at 30 °C for 20 sec, or 2, 8, or 20 min. The reaction was terminated by addition of SDS-PAGE sample buffer, and the products were resolved by SDS-PAGE. Representative degradation fragments were subjected to in-gel digestion with trypsin or Asp-N and analyzed by LC-MS/MS.
(B) Autolytic fragments of C1 or C2, deduced from the identified sequence and fragment size in SDS-PAGE, are shown schematically. When the terminal amino acid residue of peptides did not match cleavage by either trypsin or Asp-N, *i.e.*, at the C-terminus of Lys or Arg or the N-terminus of Asp or Glu, the position was defined as a calpain autolytic site. Rectangles indicate regions for which representative peptides were actually identified. The rectangles were colored according to the amino acid coverage: dark blue, > 80%; dark green, 60-80%; green, 50-60%; light green, 35-50%; yellow-green, 20-35%. Bold, calpain autolytic site; outlined, trypsin or Asp-N cleavage site; blue, estimated terminal amino acid residue number. Arrows indicate the cleavage proximal to the CuT1-3.

Shinkai-Ouchi et al, Figure S3

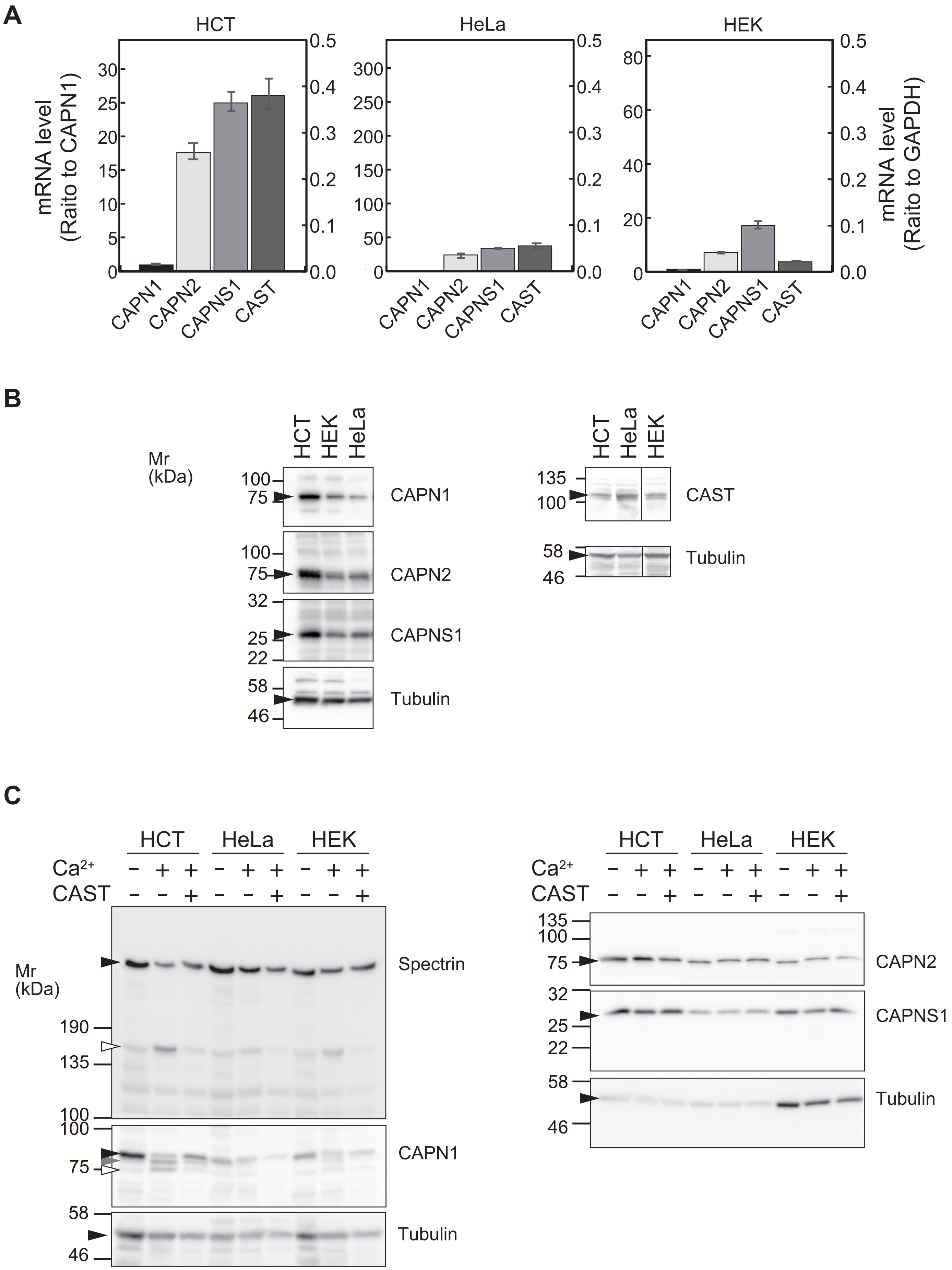

Figure S3. Expression of calpain related molecules in different cell lines.

(A) Relative amount of expressed mRNA. mRNA expression levels of CAPN1, CAPN2, CAPNS1, and CAST in HCT116, HeLa and HEK293 cells were analyzed by RT-qPCR. Reactions were carried out in triplicate and results were normalized according to GAPDH quantitation in the same reaction (right scale). Relative ratio to CAPN1 expression is also shown (left scale).

(B) Expression level of proteins. CAPN1, CAPN2, CAPNS1, and CAST protein levels in cell lysates were analyzed by western blot. Equal amounts of protein (10 µg in left panel, 27 µg in right panel) were separated by SDS-PAGE, and tubulin was used as a loading control.

(C) Calpain-dependent proteolysis of spectrin and calpains.

Left panel: Cell lysates were diluted to 1 mg/mL and incubated with 5mM $CaCl_2$ in the absence or presence of 40 nM CAST-d1 at 30 °C. Proteolysis of spectrin and autolysis of calpain-1 were analyzed by western blot. Generation of proteolyzed fragment, ca. 150 kDa, was most obviously detected in HCT116 cells. Each lane contains 10 µg of proteins.

Right panel: Cell lysates were diluted to 1 mg/mL and incubated with 5mM $CaCl_2$ in the absence or presence of 40 nM CAST-d1 at 30 °C. Autolysis of calpains were analyzed by western blot. Each lane contains 15 µg (HCT116 and HeLa cells) and 20 µg (HEK293 cell) of proteins. Activation-associated autolysis of C2 is not observable as a change of fragment size of CAPN2 [36].

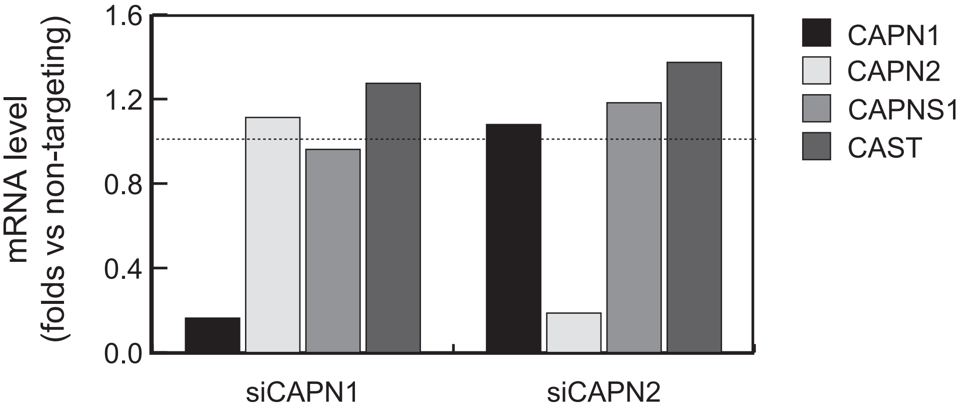

Figure S4. Downregulation of CAPN1 and CAPN2 in HCT116 cells.
HCT116 cells were transfected with siRNA targeting CAPN1 or CAPN2, or non-targeting siRNA (nt). After 24 h, the efficiency and specificity of knockdown was analyzed by RT-qPCR. Analysis was performed in duplicate, and results were normalized according to GAPDH quantitation in the same reaction. Data are presented as relative ratio to values obtained for the cells treated with nt siRNA.

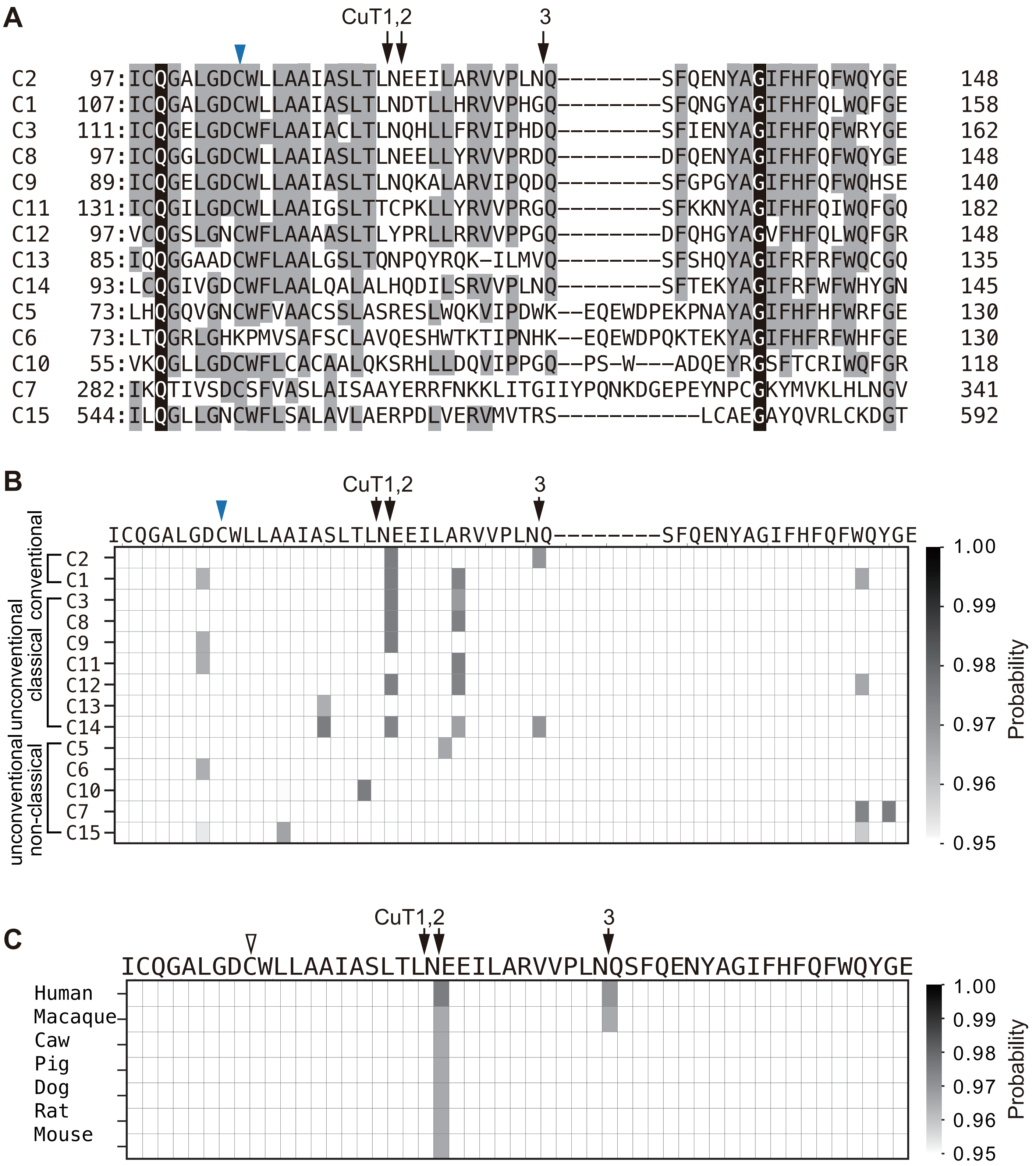

Figure S5. Cleavability prediction of human calpain PC1 domains.
(A) The amino acid sequence 91-156 aa of CAPN2 was aligned with the corresponding region form human CAPN1-15. Arrows, CuT1-3. Arrow head, active site Cys (except in CAPN6). Highly and moderately conserved residues are highlighted in black and gray, respectively. Human CAPN sequence data were retrieved from UniProtKB. CAPN1, P07384; CAPN2, P17655; CAPN3, P20807; CAPN5, O15484; CAPN6, Q9Y6Q1; CAPN7, Q9Y6W3; CAPN8, A6NHC0; CAPN9, O014815; CAPN10, Q9HC96; CAPN11, Q9UMQ6; CAPN12, Q6ZSI9; CAPN13, Q6MZZ7; CAPN14, A8MX76; CAPN15, O75808.
(B) Cleavage probabilities within the above sequences were calculated using Calpacchopper (http://calpain.org/predict.rb?cls=substrate). Results are shown for probability values larger than 0.95.
(C) Cleavage probabilities of mammalian CAPN2 orthologues were calculated using Calpacchopper.

Shinkai-Ouchi et al, Figure S6

Figure S3B (left)

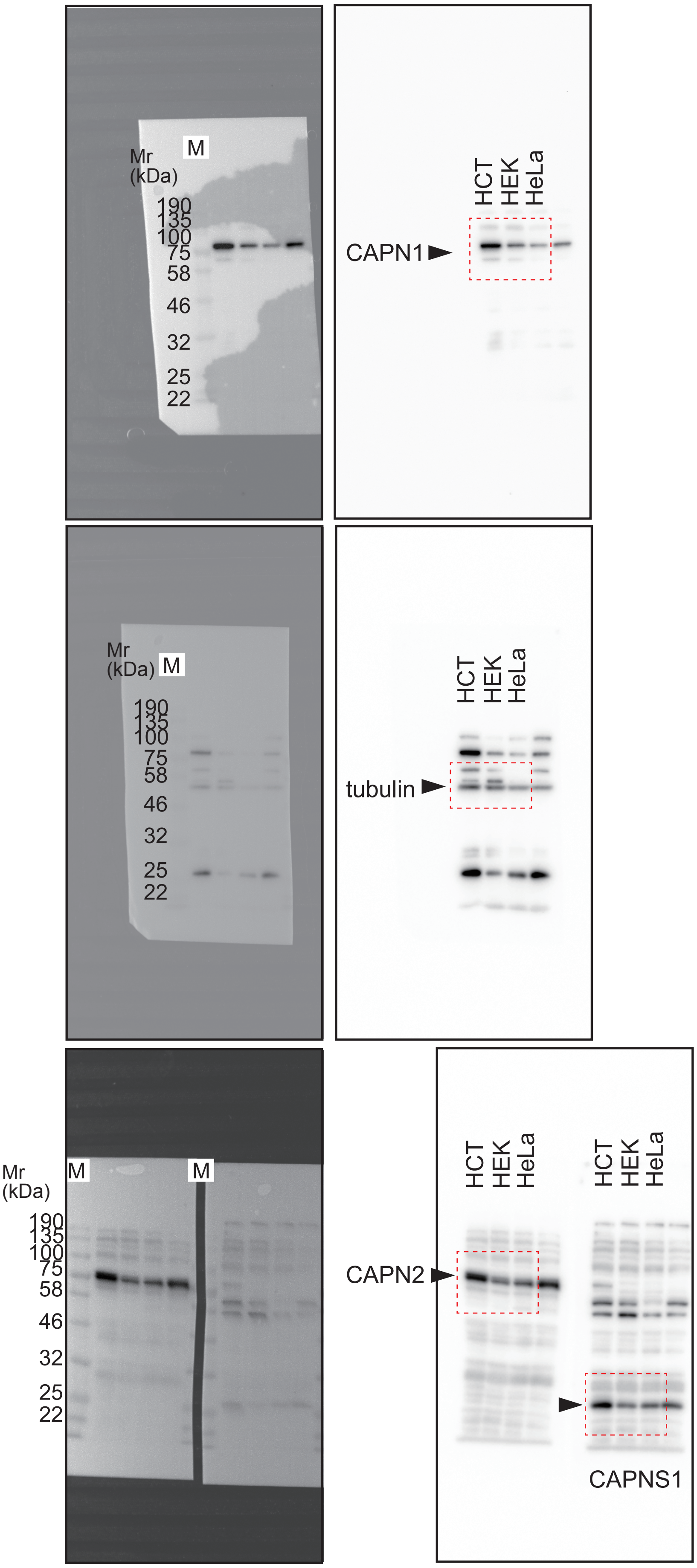

Shinkai-Ouchi et al, Figure S6, continued.

Figure S3B (right)

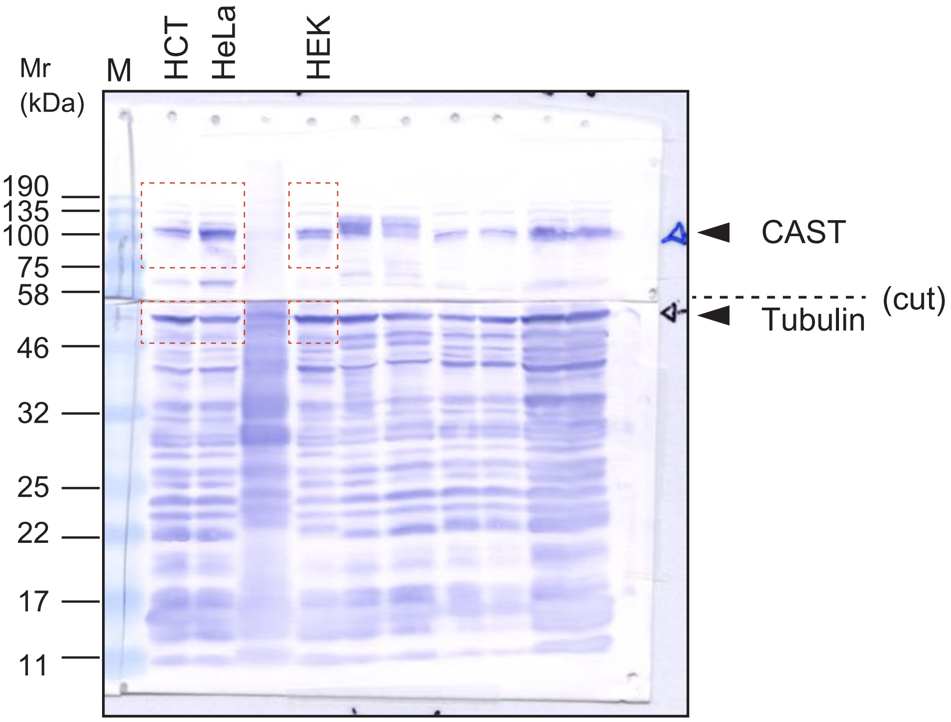

Figure S6. Uncropped images of the western blots presented in the Fig. S3B.
M: Blue prestained Protein Standard (P7706, NEB). For the right panels of Fig. S3B, signals were visualized using a POD immunostaining set. Scanned image was cropped and converted to black and white mode. Other images were acquired using FUSION FX7.EDGE (VILBER LOURMAT) by digitization (left) and chemiluminescence (right) functions.

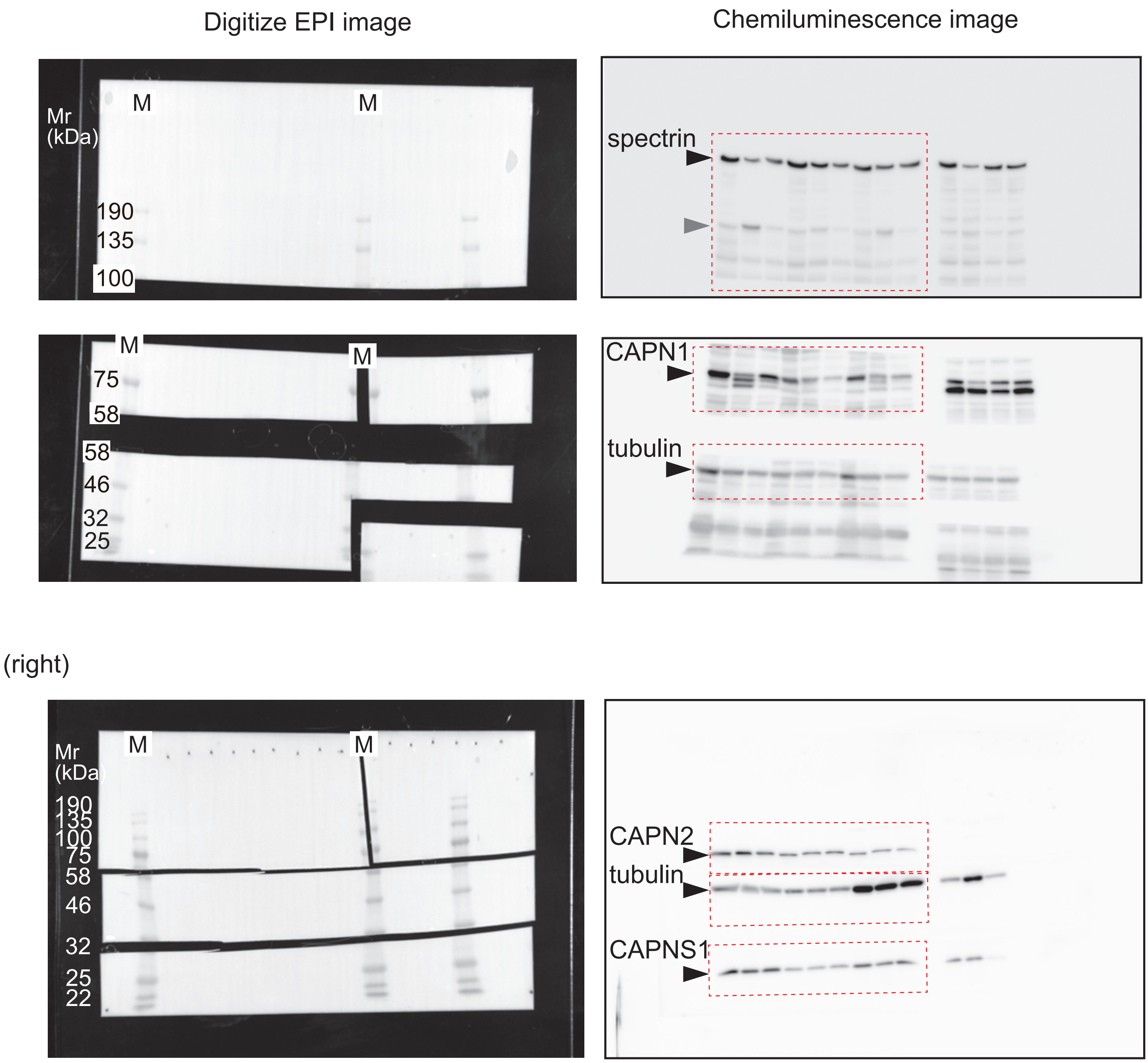

Figure S7. Uncropped images of the western blots presented in the Fig. S3C.
M: Blue prestained Protein Standard (P7706, NEB). Images were acquired using ImageQuant LAS 4000 by digitization (left) and chemiluminescence (right) functions.

Shinki-Ouchi et al, Figure S8

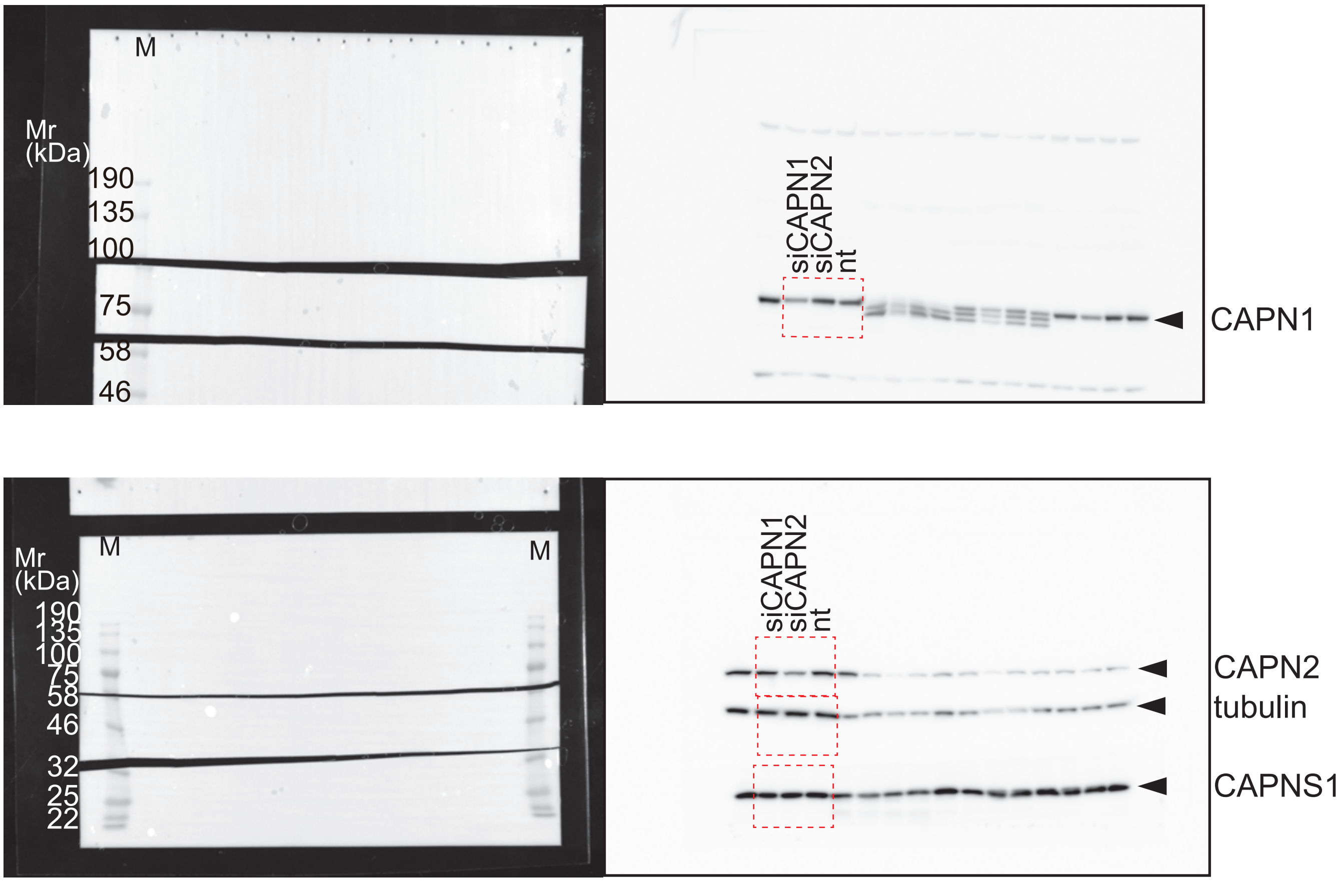

Figure S8. Uncropped images of the western blots presented in the Fig. 8A.
M: Blue prestained Protein Standard (P7706, NEB). Images were acquired using ImageQuant LAS 4000 by digitization (left) and chemiluminescence (right) functions.

Shinki-Ouchi et al, Figure S9

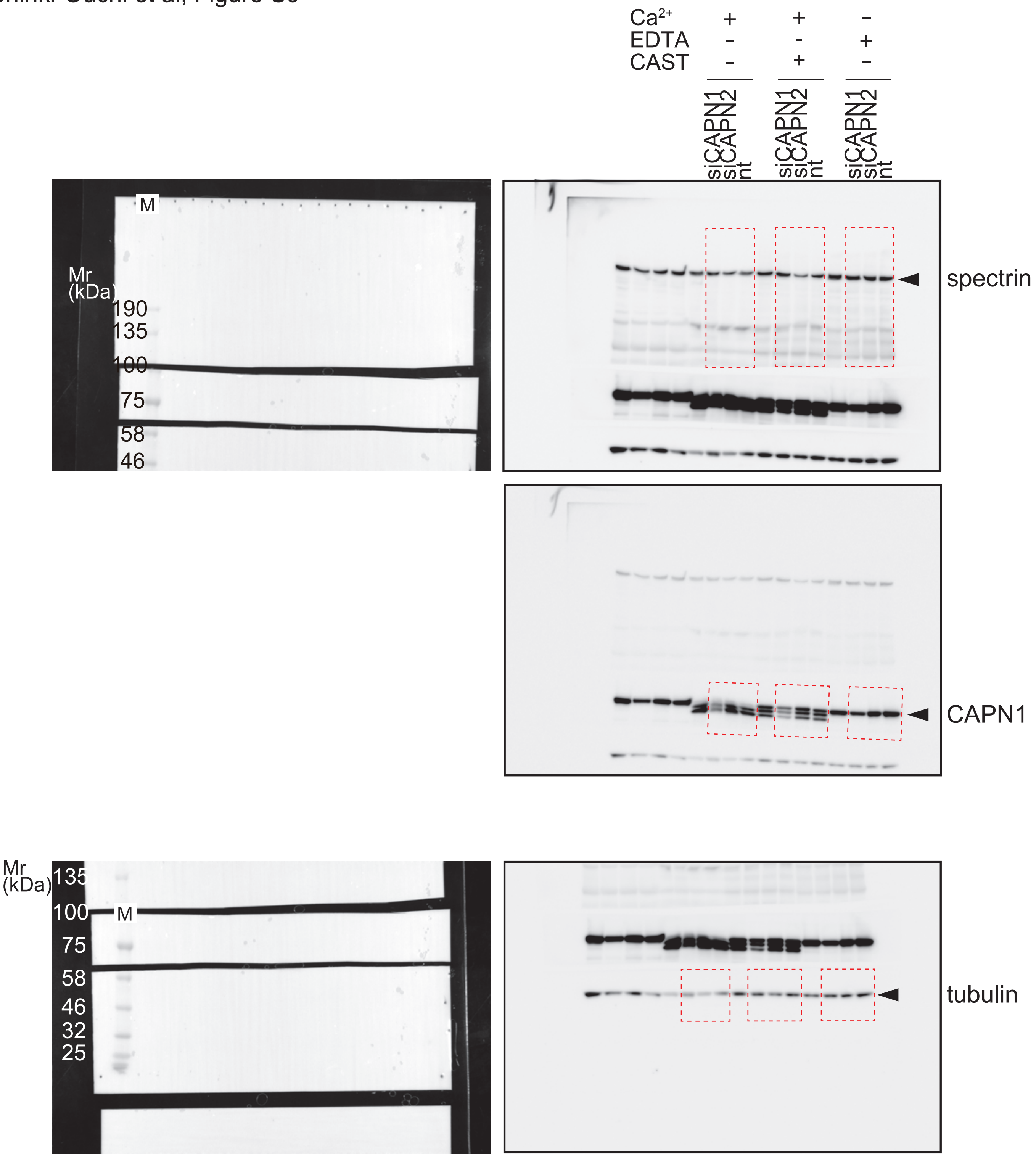

Figure S9. Uncropped images of the western blots presented in the Fig. 8B.
M: Blue prestained Protein Standard (P7706, NEB). Images were acquired using ImageQuant LAS 4000 by digitization (left) and chemiluminescence (right) functions.

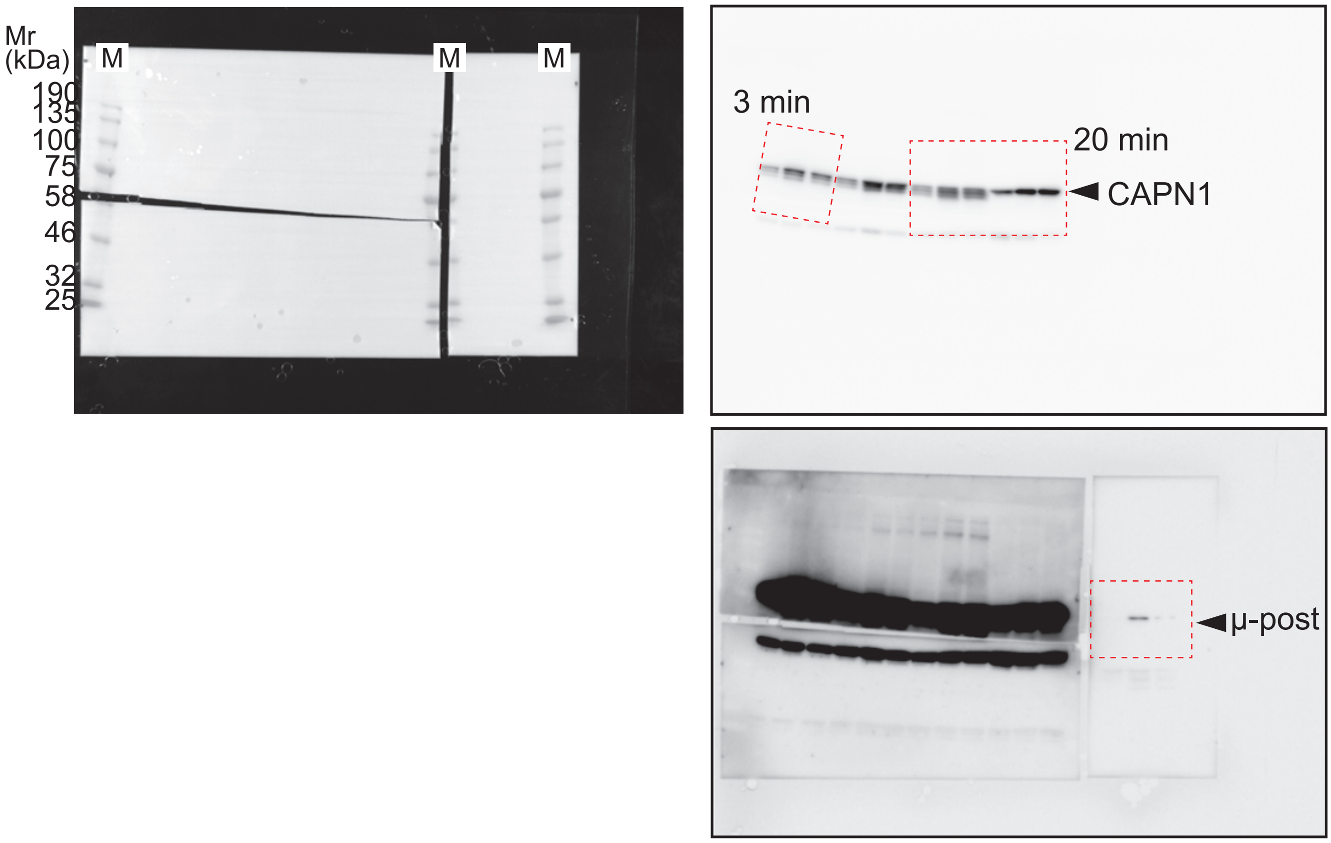

Figure S10. Uncropped images of the western blots presented in the Fig. 8C.
M: Blue prestained Protein Standard (P7706, NEB). Images were acquired using ImageQuant LAS 4000 by digitization (left) and chemiluminescence (right) functions.

Supplementary Tables

Table S1. List of Troponin T peptides generated by calpain.
Peptides were extracted from the distinct peptide list generated with ProteinPilot software by the following conditions; Accessions, P45389 TNNT2_human; "Best conf (Peptide)" > 96.8 (FDR < 1%); permitted modifications, iTRAQ at N-terminus and/or Lys, oxidation at Met, methylthio at Cys, PyroGlu at N-terminus; omit decoy sequence; quant annotation, Auto or Auto--shared MS/MS. The cleavage sites are indicated by the number of amino acid position N-terminus to the cleavage site.

Table S2. List of catalytic subunit of calpain-1 peptides generated by autolysis.
Peptides were extracted from the distinct peptide list generated with ProteinPilot software by the following conditions; Accessions, P35750_CAN1_PIG; "Best conf (Peptide)" > 96.8 (FDR < 1%); permitted modifications; iTRAQ at N-terminus and/or Lys, oxidation at Met, methylthio at Cys, PyroGlu at N-termimus; ommit decoy sequence; quant annotation, Auto or Auto--shared MS/MS. The cleavage sites are indicated by the number of amino acid position N-terminus to the cleavage site.

Table S3. List of catalytic subunit of calpain-2 peptides generated by autolysis.
Peptides were extracted from the distinct peptide list generated with ProteinPilot software by the following conditions; Accessions, P17655_CAN2_HUMAN+ "Best conf (Peptide)" > 96.8 (FDR < 1%); permitted modifications, iTRAQ at N-terminus and/or Lys, oxidation at Met, methylthio at Cys, PyroGlu at N-termimus; omit decoy sequence; quant annotation, Auto or Auto--shared MS/MS. The cleavage sites are indicated by the number of amino acid position N-terminus to the cleavage site.

Table S4. List of catalytic subunit of calpain-2 peptides generated by calpain-1.
Peptides were extracted from the distinct peptide list generated with ProteinPilot software by the following conditions; Accessions, P17655_CAN2_HUMAN; "Best conf (Peptide)" > 93.7 (FDR < 1%); permitted modifications, oxidation at Met, methylthio at Cys, PyroGlu at N-termimus; omit decoy sequence. The cleavage sites are indicated by the number of amino acid position N-terminus to the cleavage site.

Table S5. List of catalytic subunit of calpain-2 peptides generated by calpain-2.
Peptides were extracted from the distinct peptide list generated with ProteinPilot software by the following conditions; Accessions, P17655_CAN2_HUMAN; "Best conf (Peptide)" > 94.1 (FDR < 1%); permitted modifications, oxidation at Met, methylthio at Cys, PyroGlu at N-termimus; omit decoy sequence. The cleavage sites were described with number of amino acid position of N-terminus of the cleavage site. Bold letter, product of CuT cleavage. *, Peptides produced by cleavage at least one of the CuT sites.

Table S6. List of calpain small subunit peptides generated by C2.
Peptides were extracted from the distinct peptide list generated with ProteinPilot software by the following conditions; Accessions, P04632|CPNS1_HUMAN; "Best conf (Peptide)" > 81.6 (FDR < 1%); permitted modifications, iTRAQ at N-terminus and/or Lys, oxidation at Met, methylthio at Cys, PyroGlu at N-termimus; permitted quant annotation, Auto and Auto--shared MS/MS with intensity higher than 50; start position, > 25. The cleavage sites are indicated by the number of amino acid position N-terminus to the cleavage site.

Table S7. List of C2 specific peptides in PC domain generated by C2 in the presence and absence of C1:CS.
Peptides were extracted from the distinct peptide list generated with ProteinPilot software by the following conditions; Accessions, P04632|CPNS1_HUMAN; "Best conf (Peptide)" > 81.6 (FDR < 1%); permitted modifications, iTRAQ at N-terminus and/or Lys, oxidation at Met, methylthio at Cys, PyroGlu at N-termimus; permitted quant annotation, Auto and Auto--shared MS/MS with intensity higher than 50; start position, > 25. The cleavage sites are indicated by the number of amino acid position N-terminus to the cleavage site.

Table S8. List of common peptides among C1 and C2 generated by C2.
Peptides were extracted from the distinct peptide list generated with ProteinPilot software by the following conditions; Accessions, P04632|CPNS1_HUMAN; "Best conf (Peptide)" > 81.6 (FDR < 1%); permitted modifications, iTRAQ at N-terminus and/or Lys, oxidation at Met, methylthio at Cys, PyroGlu at N-termimus; permitted quant annotation, Auto and Auto--shared MS/MS with intensity higher than 50; start position, > 25. The cleavage sites are indicated by the number of amino acid position N-terminus to the cleavage site.

Table S1. List of TroponinT peptide generated by calpain.

								Intensity (iTRAQ)												cleavage sites	
Sequence	Theor z	Obs m/z	Theor m/z	dMass	Modifications	Best Conf (Peptide)	Intensity (Peptide)	C2 40min Area 114	Err 114	C2 20min Area 115	Err 115	C1 40min Area 116	Err 116	C1 20min Area 117	Err 117	C1, 40min/20min 116/117	40min C1/C2 116/114	20min C1/C2 117/115	Start Position	N site	C site
EQEEAAEEDAEAEAETEETR	3	804.0146	804.0093	0.0158	iTRAQ4plex@N-term	99	4933.25	254.71	49.08	217.13	47.95	474.39	68.11	325.01	56.75	1.460	1.862	1.497	33	32	52
QEEAAEEDAEAEAETEETRAEEDEEEEEAKEAEDGPM	4	1111.4790	1111.4633	0.0645	iTRAQ4plex@N-term; iTRAQ4plex(K)@30	99	584.36	193.53	42.66	109.63	33.61	11.42	13.78	48.01	21.21	0.238	0.059	0.438	34	33	70
QEEAAEEDAEAEAETEETRAEEDEEEEEAKEAEDGPME	4	1143.7390	1143.7239	0.0620	iTRAQ4plex@N-term; iTRAQ4plex(K)@30	99	1938.73	443	64.8	422.98	64.57	13.36	19.75	52.01	22.13	0.257	0.030	0.123	34	33	71
QEEAAEEDAEAEAETEETRAEEDEEEEEAKEAEDGPMEE	5	941.0002	940.9891	0.0555	iTRAQ4plex@N-term; iTRAQ4plex(K)@30	99	4.20E+03	662.08	79.42	795.73	88.13	109.44	39.36	199.72	43.58	0.548	0.165	0.251	34	33	72
QEEAAEEDAEAEAETEETR	3	761.0008	760.9951	0.0170	iTRAQ4plex@N-term	99	49979.21	4756.81	212.34	4531.2	214.35	4306.63	209.66	3860.11	193.91	1.116	0.905	0.852	34	33	52
QEEAAEEDAEAEAET	2	883.3817	883.3721	0.0193	iTRAQ4plex@N-term	99	420.82	31.47	17.32	37.9	20	103.27	31.62	59.84	24.49	1.726	3.282	1.579	34	33	48
QEEAAEEDAEAEAETE	2	947.9067	947.8934	0.0267	iTRAQ4plex@N-term	99	1116.61	46.97	21.21	68.07	26.45	144.07	37.41	74.21	27.38	1.941	3.067	1.090	34	33	49
EEAAEEDAEAEAETEETRAEEDEEEEEAKEAEDGPMEE	5	915.3884	915.3774	0.0553	iTRAQ4plex@N-term; iTRAQ4plex(K)@29	99	977.5	172.98	40.49	164	40.37	44.48	23.23	84.2	28.28	0.528	0.257	0.513	35	34	72
EEAAEEDAEAEAETEETR	3	718.3126	718.3089	0.0111	iTRAQ4plex@N-term	99	13651.81	807.51	87.57	837.03	92.56	1187.54	109.16	1155.65	105.86	1.028	1.471	1.381	35	34	52
EAAEEDAEAEAETEETRAEEDEEEEEAKEAEDGPME	4	1079.4630	1079.4486	0.0577	iTRAQ4plex@N-term; iTRAQ4plex(K)@28	99	1468.54	338.66	56.65	319.67	56.21	26.35	22.36	176.88	40.62	0.149	0.078	0.553	36	35	71
EAAEEDAEAEAETEETRAEEDEEEEEAKEAE	4	947.1624	947.1525	0.0396	iTRAQ4plex@N-term; iTRAQ4plex(K)@28	99	724.66	99.49	30.82	127.97	35.35	28.13	20	166.18	39.37	0.169	0.283	1.299	36	35	66
EAAEEDAEAEAETEETRAEEDEEEEEAKEAEDGPMEESKPKPRSF	7	809.3823	809.3703	0.0843	iTRAQ4plex@N-term; iTRAQ4plex(K)@28; iTRAQ4plex(K)@39; iTRAQ4plex(K)@41	99	1403.17	340.58	57.09	479.31	68.33	129.69	41.47	616.06	75.82	0.211	0.381	1.285	36	35	80
EAAEEDAEAEAETEETRAEEDEEEEEAKEAEDGPM	4	1047.2010	1047.1880	0.0520	iTRAQ4plex@N-term; iTRAQ4plex(K)@28	99	855.49	105.58	31.62	95.01	30.82	31.05	20	144.44	36.74	0.215	0.294	1.520	36	35	70
EAAEEDAEAEAETEETRAEEDEEEEEAKEAEDGPMEE	4	1111.7240	1111.7092	0.0610	iTRAQ4plex@N-term; iTRAQ4plex(K)@28	99	4.40E+03	430.98	63.94	430.13	65.34	135.08	41.23	506.13	68.84	0.267	0.313	1.177	36	35	72
EAAEEDAEAEAETEETRAEEDE	3	866.3662	866.3586	0.0226	iTRAQ4plex@N-term	99	550.99	31.68	17.32	28.46	17.32	59.04	24.49	94.65	30	0.624	1.864	3.326	36	35	57
EAAEEDAEAEAETEETRAEEDEEE	3	952.3950	952.3871	0.0239	iTRAQ4plex@N-term	99	785.65	79.25	27.38	68.3	26.45	73.04	27.38	88.33	29.15	0.827	0.922	1.293	36	35	59
EAAEEDAEAEAETEETRAEEDEEEE	3	995.4115	995.4012	0.0307	iTRAQ4plex@N-term	99	779.19	0	0.61	28.42	17.32	122.31	34.64	134.91	36.05	0.907	#DIV/0!	4.747	36	35	60
EAAEEDAEAEAETE	2	819.3516	819.3428	0.0177	iTRAQ4plex@N-term	99	2020.13	29.14	16.73	46.33	21.91	114.18	33.46	96.08	30.66	1.188	3.918	2.074	36	35	49
EAAEEDAEAEAETEETR	3	675.2988	675.2947	0.0123	iTRAQ4plex@N-term	99	64260.27	3122.91	172.2	3242.16	187.72	11297.5	331.6	9309.41	301.95	1.214	3.618	2.871	36	35	52
AAEEDAEAEAETEETRAEEDEEEEEAKEAEDGPME	4	1047.2000	1047.1880	0.0500	iTRAQ4plex@N-term; iTRAQ4plex(K)@27	99	2125.33	631.07	77.19	483.3	69.49	0	21.04	151.7	37.41	0.000	0.000	0.314	37	36	71
AAEEDAEAEAETEETRAEEDEEEEEAKEAEDGPMEESKPKPRSF	7	790.9426	790.9356	0.0490	iTRAQ4plex@N-term; iTRAQ4plex(K)@27; iTRAQ4plex(K)@38; iTRAQ4plex(K)@40	99	5605.57	680.88	80.98	1173.5	106.14	106.87	43.01	479.49	67	0.223	0.157	0.409	37	36	80
AAEEDAEAEAETEETRAEEDEEEEEAKEAEDGPMEE	4	1079.4620	1079.4486	0.0533	iTRAQ4plex@N-term; iTRAQ4plex(K)@27	99	7503.08	749.58	84.55	932.39	95.28	93.37	39.36	375.46	59.32	0.249	0.125	0.403	37	36	72
AAEEDAEAEAETEETRAEEDEEEEEAKEAEDGPM	4	1014.9410	1014.9273	0.0533	iTRAQ4plex@N-term; iTRAQ4plex(K)@27	99	668.42	178.1	41.23	226.22	46.9	20.55	18.71	74.63	26.45	0.275	0.115	0.330	37	36	70
AAEEDAEAEAETEETRAEEDEEEEEAKEAE	4	914.9003	914.8919	0.0338	iTRAQ4plex@N-term; iTRAQ4plex(K)@27	99	694.92	122.26	34.35	220.41	46.15	74.46	29.32	115.36	33.16	0.645	0.609	0.523	37	36	66
AAEEDAEAEAETEETR	3	632.2856	632.2805	0.0152	iTRAQ4plex@N-term	99	31342.87	2742.47	161.53	3109.98	177.34	3702.27	192.46	2611.82	160.64	1.418	1.350	0.840	37	36	52
AEEDAEAEAETEETRAEEDEEEEEAKEAEDGPMEESKPKPRSF	6	910.7616	910.7509	0.0646	iTRAQ4plex@N-term; iTRAQ4plex(K)@26; iTRAQ4plex(K)@37; iTRAQ4plex(K)@39	99	726.58	187.03	42.18	212.88	45.6	12.34	16.73	107.56	31.62	0.115	0.066	0.505	38	37	80
AEEDAEAEAETEETRAEEDEEEEEAKEAEDGPMEE	4	1061.7020	1061.6893	0.0523	iTRAQ4plex@N-term; iTRAQ4plex(K)@26	99	2313.17	426.22	63.87	599.66	76.21	69.79	33.16	277.24	50.99	0.252	0.164	0.462	38	37	72
AEEDAEAEAETEETRAEEDEEEEEAKEAEDGPME	4	1029.4400	1029.4287	0.0461	iTRAQ4plex@N-term; iTRAQ4plex(K)@26	99	821.01	137.32	36.05	120.61	34.64	18.09	15.81	31.44	17.32	0.575	0.132	0.261	38	37	71
AEEDAEAEAETEETR	3	608.6056	608.6015	0.0123	iTRAQ4plex@N-term	99	14385.59	926.82	93.85	999.7	101.67	1846.84	134.59	1192.68	108.91	1.548	1.993	1.193	38	37	52
EDAEAEAETEETR	3	541.9209	541.9083	0.0379	iTRAQ4plex@N-term	99	416.34	136.99	36.05	136.2	37.41	202.94	44.71	114.61	33.91	1.771	1.481	0.841	40	39	52

AEAEAETEETRAEEDEEEEEAKEAEDGPMEESKPKPRSF	7	717.3424	717.3376	0.0339	iTRAQ4plex@N-term; iTRAQ4plex(K)@22; iTRAQ4plex(K)@33; iTRAQ4plex(K)@35	99	7256.59	1401.42	115.66	1796.28	133.85	1582.01	131.17	3896.87	191.66	0.406	1.129	2.169	42	41	80
AEAEAETEETRAEEDEEEEEAKEAEDGPMEES	4	972.4189	972.4100	0.0355	iTRAQ4plex@N-term; iTRAQ4plex(K)@22	99	692.45	30.57	17.32	80	28.28	140.82	37.41	133.69	36.05	1.053	4.606	1.671	42	41	73
AEAEAETEETRAEEDEEEEEAKEAEDGPMEE	4	950.6606	950.6520	0.0343	iTRAQ4plex@N-term; iTRAQ4plex(K)@22	99	23813.87	1670.99	126.24	2078.79	147.66	5308.69	228.05	4426.59	208.09	1.199	3.177	2.129	42	41	72
AEAEAETEETRAEEDEEEEEA	3	847.3561	847.3515	0.0137	iTRAQ4plex@N-term	98.2	478.62	52.72	22.36	51.08	23.45	154.29	38.72	111.64	33.16	1.382	2.927	2.186	42	41	62
AEAEAETEETRAEEDE	3	651.6210	651.6157	0.0158	iTRAQ4plex@N-term	99	1033.4	135.78	35.91	143.12	39.74	582.59	75.02	410.07	63.63	1.421	4.291	2.865	42	41	57
AEAEAETEETRAEEDEEE	3	737.6527	737.6441	0.0259	iTRAQ4plex@N-term	99	1051.14	36.37	18.71	60.6	26.45	388.99	61.06	252.73	50.09	1.539	10.695	4.170	42	41	59
AEAEAETEETRAEEDEEEEEAKEAEDGPME	4	918.3989	918.3914	0.0303	iTRAQ4plex@N-term; iTRAQ4plex(K)@22	99	4036.3	507.13	69.49	591.17	77.13	634.87	79.74	381.7	61.81	1.663	1.252	0.646	42	41	71
AEAEAETEETRAEEDEEEEEAKEAE	4	786.1006	786.0953	0.0212	iTRAQ4plex@N-term; iTRAQ4plex(K)@22	99	1741.74	199.02	43.58	255.84	52.05	755.69	85.49	450.36	67.07	1.678	3.797	1.760	42	41	66
AEAEAETEETRAEEDEEEEEAKEAEDGPM	4	886.1376	886.1307	0.0275	iTRAQ4plex@N-term; iTRAQ4plex(K)@22	99	2369.82	200.41	43.7	240.05	50.98	870.15	91.53	503.68	71.07	1.728	4.342	2.098	42	41	70
AEAEAETEETR	2	690.3293	690.3240	0.0106	iTRAQ4plex@N-term	99	4102.18	232.18	47	265.51	56.29	1911.99	134.78	874.77	94.54	2.186	8.235	3.295	42	41	52
AEAEAETEETRAEEDEEEE	3	780.6638	780.6583	0.0166	iTRAQ4plex@N-term	99	1954.63	46.94	21.21	69.27	29.15	600.88	75.49	270.53	52.62	2.221	12.801	3.905	42	41	60
AEAETEETRAEEDEEEEEAKEAEDGPMEESKPKPRSF	6	803.3840	803.3793	0.0278	iTRAQ4plex@N-term; iTRAQ4plex(K)@20; iTRAQ4plex(K)@31; iTRAQ4plex(K)@33	99	1046.83	195.12	43.01	185.73	44.15	377.66	62.36	731.26	83.17	0.516	1.936	3.937	44	43	80
AEAETEETRAEEDEEEEEAKEAEDGPMEE	4	900.6394	900.6321	0.0293	iTRAQ4plex@N-term; iTRAQ4plex(K)@20	99	3996.62	340.42	56.82	335.75	60.32	1106.14	103.67	836.53	90.72	1.322	3.249	2.492	44	43	72
AEAETEETRAEEDEEEEEAKEAE	4	736.0788	736.0754	0.0137	iTRAQ4plex@N-term; iTRAQ4plex(K)@20	99	563.08	51.47	22.36	109.17	33.91	366.71	59.41	198.59	44.72	1.847	7.125	1.819	44	43	66
AEAETEETR	2	590.2864	590.2841	0.0045	iTRAQ4plex@N-term	99	462.31	29.51	16.73	28.95	18.97	247.27	48.47	121.07	35.07	2.042	8.379	4.182	44	43	52
AEAETEETRAEEDEEEEEAKEAEDGPM	4	836.1137	836.1108	0.0117	iTRAQ4plex@N-term; iTRAQ4plex(K)@20	98.4	417.86	95.85	30	47.35	24.49	349.95	57.61	145.18	38.73	2.410	3.651	3.066	44	43	70
AEAETEETRAEEDEEEEEAKEAEDGPME	4	868.3854	868.3714	0.0558	iTRAQ4plex@N-term; iTRAQ4plex(K)@20	99	461.92	137.1	36.05	130.93	36.74	199.96	44.15	65.85	26.45	3.037	1.458	0.503	44	43	71
EAETEETRAEEDEEEEEAKEAEDGPMEE	4	882.8912	882.8728	0.0735	iTRAQ4plex@N-term; iTRAQ4plex(K)@19	99	633.28	90.68	29.32	87.48	30.98	323	55.85	211.69	45.82	1.526	3.562	2.420	45	44	72
AETEETRAEEDEEEEEAKEAEDGPMEESKPKPRSF	6	770.0386	770.0327	0.0351	iTRAQ4plex@N-term; iTRAQ4plex(K)@18; iTRAQ4plex(K)@29; iTRAQ4plex(K)@31	99	2618.61	485.19	67.81	460.37	69.49	904.92	97.71	2302.33	147.15	0.393	1.865	5.001	46	45	80
AETEETRAEEDEEEEEAKEAEDGPMEE	4	850.6201	850.6121	0.0319	iTRAQ4plex@N-term; iTRAQ4plex(K)@18	99	6684.81	448.98	65.34	495.4	73.2	1706.28	128.36	1069.33	103.2	1.596	3.800	2.159	46	45	72
AETEETRAEEDEEEEEAKEAEDGPME	4	818.3576	818.3515	0.0244	iTRAQ4plex@N-term; iTRAQ4plex(K)@18	99	1518.14	200.29	43.58	195.54	44.83	284.25	53	175.27	41.83	1.622	1.419	0.896	46	45	71
AETEETRAEEDEEEEEAKEAE	4	686.0613	686.0554	0.0234	iTRAQ4plex@N-term; iTRAQ4plex(K)@18	99	513.54	52.01	22.36	83.98	30	310.42	54.4	109.2	33.91	2.843	5.968	1.300	46	45	66
TEETRAEEDEEEEEAKEAEDGPMEE	4	800.5977	800.5922	0.0220	iTRAQ4plex@N-term; iTRAQ4plex(K)@16	99	1095.11	105.69	31.62	90.69	31.62	328.44	56.29	205.89	45.27	1.595	3.108	2.270	48	47	72
EETRAEEDEEEEEAKEAEDGPMEESKPKPRSF	6	719.8503	719.8448	0.0328	iTRAQ4plex@N-term; iTRAQ4plex(K)@15; iTRAQ4plex(K)@26; iTRAQ4plex(K)@28	99	1700.24	156.27	38.72	240.38	50.09	630.74	79.74	985.41	96.84	0.640	4.036	4.099	49	48	80
ETRAEEDEEEEEAKEAEDGPMEESKPKPRSF	6	698.3424	698.3377	0.0281	iTRAQ4plex@N-term; iTRAQ4plex(K)@14; iTRAQ4plex(K)@25; iTRAQ4plex(K)@27	99	5606.1	609.57	76.47	936.01	97.81	1777.2	135.88	3781.6	189.03	0.470	2.915	4.040	50	49	80
ETRAEEDEEEEEAKEAEDGPM	4	678.5513	678.5483	0.0117	iTRAQ4plex@N-term; iTRAQ4plex(K)@14	99	1683.31	104.34	31.62	153.7	40.12	420.72	64.18	361.23	59.41	1.165	4.032	2.350	50	49	70
ETRAEEDEEEEEAKEAEDGPMEE	4	743.0740	743.0696	0.0173	iTRAQ4plex@N-term; iTRAQ4plex(K)@14	99	13255.46	506.91	69.63	701.37	85.49	1744.68	130.67	1391.13	116.79	1.254	3.442	1.983	50	49	72
ETRAEEDEEEEEAKEAEDGPME	4	710.8138	710.8090	0.0191	iTRAQ4plex@N-term; iTRAQ4plex(K)@14	99	2952.8	230.36	47	349.93	58.81	307.38	55.85	198.02	44.38	1.552	1.334	0.566	50	49	71
ETRAEEDEEEEEAKEAE	4	578.5145	578.5129	0.0063	iTRAQ4plex@N-term; iTRAQ4plex(K)@14	99	874.23	72.65	26.45	125.21	36.05	332.62	56.65	162.49	40.61	2.047	4.578	1.298	50	49	66
TRAEEDEEEEEAKEAEDGPMEESKPKPRSF	6	676.8337	676.8306	0.0183	iTRAQ4plex@N-term; iTRAQ4plex(K)@13; iTRAQ4plex(K)@24; iTRAQ4plex(K)@26	99	2024.87	222.99	45.82	140.81	39.99	533.42	73.2	870.05	91	0.613	2.392	6.179	51	50	80

TRAEEDEEEEEAKEAE	4	546.2877	546.2523	0.1417	iTRAQ4plex@N-term;<br>iTRAQ4plex(K)@13	98.7	272.48	99.77	30.98	166.07	40.98	285.08	53.28	277.2	51.86	1.028	2.857	1.669	51	50	66
TRAEEDEEEEEAKEAEDGPME	4	678.5553	678.5483	0.0278	iTRAQ4plex@N-term;<br>iTRAQ4plex(K)@13	99	698.22	102.78	31.62	226.13	46.9	169.28	41.83	142.97	37.41	1.184	1.647	0.632	51	50	71
TRAEEDEEEEEAKEAEDGPMEE	4	710.8138	710.8090	0.0191	iTRAQ4plex@N-term;<br>iTRAQ4plex(K)@13	99	6758.27	220.84	45.93	290.74	54.95	675.56	80.92	351.48	59.57	1.922	3.059	1.209	51	50	72
RAEEDEEEEEAKEAEDGPMEESKPKPRSF	6	659.9934	659.9893	0.0244	iTRAQ4plex@N-term;<br>iTRAQ4plex(K)@12;<br>iTRAQ4plex(K)@23;<br>iTRAQ4plex(K)@25	99	1117.97	168.07	39.99	192.34	44.71	395.77	63.55	680.22	80.36	0.582	2.355	3.537	52	51	80
RAEEDEEEEEAKEAEDGPMEE	4	685.5495	685.5471	0.0097	iTRAQ4plex@N-term;<br>iTRAQ4plex(K)@12	99	4283.3	178.24	41.23	220.83	48.06	544.1	72.72	338.25	58.04	1.609	3.053	1.532	52	51	72
AEEDEEEEEAKEAEDGPMEESKPKPR	6	594.9570	594.9557	0.0073	iTRAQ4plex@N-term;<br>iTRAQ4plex(K)@11;<br>iTRAQ4plex(K)@22;<br>iTRAQ4plex(K)@24	99	4551.77	559.28	73.13	771.3	88.98	1463.14	123.43	3194.27	173.66	0.458	2.616	4.141	53	52	78
AEEDEEEEEAKEAEDGPMEESKPKPRSF	5	760.5712	760.5656	0.0285	iTRAQ4plex@N-term;<br>iTRAQ4plex(K)@11;<br>iTRAQ4plex(K)@22;<br>iTRAQ4plex(K)@24	99	88559.18	8907.22	291.15	10191.1	321.56	12824.4	367.29	26401.4	501.05	0.486	1.440	2.591	53	52	80
AEEDEEEEEAKEAEDGPMEESKPKPRS	5	731.1567	731.1519	0.0241	iTRAQ4plex@N-term;<br>iTRAQ4plex(K)@11;<br>iTRAQ4plex(K)@22;<br>iTRAQ4plex(K)@24	99	2465.6	496.64	68.91	681.02	82.21	571.41	77.83	972.3	96.11	0.588	1.151	1.428	53	52	79
AEEDEEEEEAKEAED	3	680.6312	680.6267	0.0136	iTRAQ4plex@N-term;<br>iTRAQ4plex(K)@11	99	1202.36	125.95	34.78	199.76	44.94	330.14	57.26	287.95	53	1.147	2.621	1.441	53	52	67
AEEDEEEEEAKEA	3	599.2715	599.2701	0.0039	iTRAQ4plex@N-term;<br>iTRAQ4plex(K)@11	99	740.48	104.69	31.62	137.51	38.07	387.7	61.39	271.4	51.76	1.429	3.703	1.974	53	52	65
AEEDEEEEEAKEAEDGPMEES	3	890.7123	890.7040	0.0249	iTRAQ4plex@N-term;<br>iTRAQ4plex(K)@11	99	6953.63	872.23	90.98	885.12	95.64	1518.66	122.25	1011.27	100.14	1.502	1.741	1.143	53	52	73
AEEDEEEEEAKEAEDGPMEE	3	861.7006	861.6933	0.0219	iTRAQ4plex@N-term;<br>iTRAQ4plex(K)@11	99	150313.9	18025.5	413.96	19710.5	453.77	43733.5	653.34	28694.1	534.75	1.524	2.426	1.456	53	52	72
AEEDEEEEEAKEAEDGPM	3	775.6702	775.6649	0.0159	iTRAQ4plex@N-term;<br>iTRAQ4plex(K)@11	99	29119.96	1453.85	117.63	1669.54	132.61	4317.58	204.76	2705.35	164.3	1.596	2.970	1.620	53	52	70
AEEDEEEEEAKEAEDGPME	3	818.6857	818.6791	0.0198	iTRAQ4plex@N-term;<br>iTRAQ4plex(K)@11	99	51730.09	5207.87	222.45	5596.42	236.21	3537.7	191.73	2110.13	145.51	1.677	0.679	0.377	53	52	71
AEEDEEEEEAKEAE	3	642.2900	642.2844	0.0168	iTRAQ4plex@N-term;<br>iTRAQ4plex(K)@11	99	8030.67	885.61	92.07	1264.25	115.61	3871.13	192.79	1712.64	132.5	2.260	4.371	1.355	53	52	66
EEDEEEEEAKEAEDGPMEE	3	838.0193	838.0143	0.0150	iTRAQ4plex@N-term;<br>iTRAQ4plex(K)@10	99	620.24	176.94	41.23	280.39	53.1	411.12	64.26	426.89	64.26	0.963	2.323	1.522	54	53	72
EEEEAKEAEDGPMEESKPKPRSF	5	645.9294	645.9272	0.0111	iTRAQ4plex@N-term;<br>iTRAQ4plex(K)@6;<br>iTRAQ4plex(K)@17;<br>iTRAQ4plex(K)@19	99	3487.92	290.88	52.81	436.95	66.92	860.66	94.27	1717.64	127.46	0.501	2.959	3.931	58	57	80
EEEEAKEAEDGPME	3	627.6194	627.6152	0.0126	iTRAQ4plex@N-term;<br>iTRAQ4plex(K)@6	98	383.81	72.93	26.45	112.12	33.91	235.43	47.95	150.89	38.72	1.560	3.228	1.346	58	57	71
EEEEAKEAEDGPMEE	3	670.6310	670.6294	0.0048	iTRAQ4plex@N-term;<br>iTRAQ4plex(K)@6	99	3380.44	109.5	32.4	163.35	45.38	1595.04	123.27	920.87	96.09	1.732	14.567	5.637	58	57	72
EEAKEAEDGPMEESKPKPRSF	5	594.3119	594.3101	0.0088	iTRAQ4plex@N-term;<br>iTRAQ4plex(K)@4;<br>iTRAQ4plex(K)@15;<br>iTRAQ4plex(K)@17	99	7702.38	331.53	56.65	650.89	80.98	1141.37	107.18	1431.41	117.16	0.797	3.443	2.199	60	59	80
EEAKEAEDGPMEE	3	584.6033	584.6010	0.0069	iTRAQ4plex@N-term;<br>iTRAQ4plex(K)@4	99	1899.77	126.68	34.64	115	36.05	533.01	71.75	395.16	62.36	1.349	4.208	3.436	60	59	72
EAKEAEDGPMEESKPKPRSF	5	568.5039	568.5016	0.0115	iTRAQ4plex@N-term;<br>iTRAQ4plex(K)@3;<br>iTRAQ4plex(K)@14;<br>iTRAQ4plex(K)@16	99	13768	1219.83	107.83	1482.2	126.95	5546.78	233.05	5559.26	232.03	0.998	4.547	3.751	61	60	80
KEAEDGPMEESKPKPRSF	5	528.4879	528.4857	0.0112	iTRAQ4plex@N-term;<br>iTRAQ4plex(K)@1;<br>iTRAQ4plex(K)@12;<br>iTRAQ4plex(K)@14	99	4297.12	973.87	96.32	1158.66	109.07	2039.87	142.39	1909.2	136.31	1.068	2.095	1.648	63	62	80
EAEDGPMEESKPKPRSF	4	592.3072	592.3060	0.0047	iTRAQ4plex@N-term;<br>iTRAQ4plex(K)@11;<br>iTRAQ4plex(K)@13	99	398.94	92.95	30	182.13	42.42	205.4	45.82	245.53	48.57	0.837	2.210	1.348	64	63	80

AEDGPMEESKPKPRSF	4	560.0480	560.0453	0.0105	iTRAQ4plex@N-term; iTRAQ4plex(K)@10; iTRAQ4plex(K)@12	99	564.79	158.23	38.72	149.16	39.49	290.74	53.66	267.16	50.98	1.088	1.837	1.791	65	64	80
EDGPMEESKPKPRSF	4	542.2877	542.2861	0.0066	iTRAQ4plex@N-term; iTRAQ4plex(K)@9; iTRAQ4plex(K)@11	99	3561.12	486.97	67.96	477.79	71.68	1429.61	118.47	1382.66	115.78	1.034	2.936	2.894	66	65	80
DGPMEESKPKPRSF	4	510.0276	510.0254	0.0086	iTRAQ4plex@N-term; iTRAQ4plex(K)@8; iTRAQ4plex(K)@10	99	22736.96	7540.56	268.12	9288.15	307.93	15119.6	387.86	13557.9	363.35	1.115	2.005	1.460	67	66	80
GPMEESKPKPRSF	4	481.2684	481.2687	-0.0012	iTRAQ4plex@N-term; iTRAQ4plex(K)@7; iTRAQ4plex(K)@9	99	4938.27	2106.93	141.4	2127.27	147.56	2929.11	170.97	2354.13	151.87	1.244	1.390	1.107	68	67	80
MPNLVPPKIPDGER	3	617.6872	617.6849	0.0070	iTRAQ4plex@N-term; iTRAQ4plex(K)@8	99	1507.71	291.69	52.62	301.11	56.82	905.79	93.9	690.15	82.33	1.312	3.105	2.292	81	80	94
MPNLVPPKIPDGERVDFDDIH	4	673.8616	673.8557	0.0238	iTRAQ4plex@N-term; iTRAQ4plex(K)@8	99	48922.52	7473.09	266.21	7322.62	273.55	9312.88	304.37	5924.08	242.95	1.572	1.246	0.809	81	80	101
LVPPKIPDGERVDFDDIH	4	588.3235	588.3216	0.0076	iTRAQ4plex@N-term; iTRAQ4plex(K)@5	99	7654.75	2043.53	139.23	2027.98	144.03	2709.51	163.5	1319.55	115.9	2.053	1.326	0.651	84	83	101
VPPKIPDGERVDFDDIHRKRMEKDL	6	597.8391	597.8345	0.0275	iTRAQ4plex@N-term; iTRAQ4plex(K)@4; iTRAQ4plex(K)@19; iTRAQ4plex(K)@23	98.2	16277.11	7824.28	273.13	9641.44	309.31	6603.83	266.53	11212.8	326.41	0.589	0.844	1.163	85	84	109
VPPKIPDGERVDFDDIHRKRME	6	514.4512	514.4498	0.0079	iTRAQ4plex@N-term; iTRAQ4plex(K)@4; iTRAQ4plex(K)@19	98.5	12714.19	5055.72	219.06	5149.47	229.05	6335.78	253.34	6682.38	254.16	0.948	1.253	1.298	85	84	106
VPPKIPDGERVDFDDIHRKR	5	565.1247	565.1218	0.0147	iTRAQ4plex@N-term; iTRAQ4plex(K)@4; iTRAQ4plex(K)@19	99	9631.63	4331.64	202.5	3888.25	199.72	4744.87	217.68	3233.72	178.95	1.467	1.095	0.832	85	84	104
VPPKIPDGERVDFDDIH	4	560.0532	560.0506	0.0106	iTRAQ4plex@N-term; iTRAQ4plex(K)@4	99	75156.03	18565.5	419.73	18690.7	436.48	23248.9	480.27	12683.5	357.67	1.833	1.252	0.679	85	84	101
VDFDDIHRKRMEKDLN	4	616.5870	616.5839	0.0122	iTRAQ4plex@N-term; iTRAQ4plex(K)@9; iTRAQ4plex(K)@13	98.9	4886.59	671.24	79.86	719.01	89.32	3089.27	172.57	2073.15	143.31	1.490	4.602	2.883	95	94	110
MEKDLNELQALIE	3	612.0038	612.0005	0.0098	iTRAQ4plex@N-term; iTRAQ4plex(K)@3	99	973.08	150.41	37.81	162.71	39.99	29.65	19.75	54.72	22.8	0.542	0.197	0.336	105	104	117
KDLNELQALIEAHFEN	3	724.7286	724.7228	0.0173	iTRAQ4plex@N-term; iTRAQ4plex(K)@1	99	507.05	31.62	17.32	31.32	17.89	35.12	20.25	168.31	39.62	0.209	1.111	5.374	107	106	122
KDLNELQALIEAHF	3	643.6989	643.6943	0.0138	iTRAQ4plex@N-term; iTRAQ4plex(K)@1	99	1025.08	196.52	43.12	171.31	41.23	8.75	13.78	23.23	14.83	0.377	0.045	0.136	107	106	120
KDLNELQALIEAH	3	594.6741	594.6715	0.0077	iTRAQ4plex@N-term; iTRAQ4plex(K)@1	99	1396.29	317.75	55.04	390.89	61.8	75.68	31.62	188.71	42.18	0.401	0.238	0.483	107	106	119
NELQALIEAHF	2	714.8889	714.8838	0.0102	iTRAQ4plex@N-term	99	5022.4	901.53	92.45	874.5	92.72	0	25.81	125.41	34.05	0.000	0.000	0.143	110	109	120
NELQALIEAHFEN	3	557.9557	557.9535	0.0067	iTRAQ4plex@N-term	99	2119.63	104.09	31.46	114.59	33.76	48.3	27.74	643.85	77.26	0.075	0.464	5.619	110	109	122
NELQALIEAH	2	641.3521	641.3496	0.0050	iTRAQ4plex@N-term	99	1464.12	280.42	51.66	322.87	56.21	41.84	25.49	192.55	42.42	0.217	0.149	0.596	110	109	119
NELQALIEAHFE	3	519.9394	519.9391	0.0007	iTRAQ4plex@N-term	98.1	380.77	114.26	32.86	92.89	30.49	16.09	14.83	42.48	20	0.379	0.141	0.457	110	109	121
NELQALIEAHFENRK	4	525.7922	525.7914	0.0029	iTRAQ4plex@N-term; iTRAQ4plex(K)@15	99	4257.92	950.89	96.37	2278.76	146.98	49.22	42.42	125.17	34.64	0.393	0.052	0.055	110	109	124
ELQALIEAHFE	2	722.3887	722.3837	0.0101	iTRAQ4plex@N-term	99	2638.85	416.37	62.84	407.81	63.32	0	16.83	169.73	39.62	0.000	0.000	0.416	111	110	121
ELQALIEAHFENRK	4	497.2813	497.2807	0.0023	iTRAQ4plex@N-term; iTRAQ4plex(K)@14	99	14173.12	5798.91	235.87	8876.82	292.07	0	114.51	367.53	58.64	0.000	0.000	0.041	111	110	124
ELQALIEAH	2	584.3286	584.3281	0.0010	iTRAQ4plex@N-term	99	13123.82	2188.36	144.24	2399.5	153.31	191.47	60.98	1408.72	114.54	0.136	0.087	0.587	111	110	119
ELQALIEAHFENRKKEEEELV	4	747.4169	747.4107	0.0247	iTRAQ4plex@N-term; iTRAQ4plex(K)@14; iTRAQ4plex(K)@15	99	446.07	121.31	34.2	215.2	45.38	6.86	13.78	9.17	9.49	0.748	0.057	0.043	111	110	131
QALIEAHFENRK	4	436.7467	436.7491	-0.0094	iTRAQ4plex@N-term; iTRAQ4plex(K)@12	98	3467.37	869	90.6	683.08	83.47	488.92	71.75	581.49	74.83	0.841	0.563	0.851	113	112	124
QALIEAHFEN	2	658.3440	658.3418	0.0044	iTRAQ4plex@N-term	99	5889.74	177.49	40.98	154.7	45.27	1664.79	127.22	1793.27	131.56	0.928	9.380	11.592	113	112	122
ALIEAHFENRKKEEEELVSLKDRIE	5	721.2123	721.2065	0.0288	iTRAQ4plex@N-term; iTRAQ4plex(K)@11; iTRAQ4plex(K)@12; iTRAQ4plex(K)@21	99	4055.8	976.04	96.47	1210.01	108.47	60.29	39.49	764.62	84.25	0.079	0.062	0.632	114	113	138
ALIEAHFENRKKEEEELVSLKDR	5	672.7847	672.7812	0.0177	iTRAQ4plex@N-term; iTRAQ4plex(K)@11; iTRAQ4plex(K)@12; iTRAQ4plex(K)@21	99	4720.24	897.09	92.67	1281	111.65	278.43	68.18	3356.24	176.52	0.083	0.310	2.620	114	113	136

ALIEAHFENRKKEEEELVSL	4	704.9005	704.8934	0.0284	iTRAQ4plex@N-term; iTRAQ4plex(K)@11; iTRAQ4plex(K)@12	99	1844.88	71.23	26.07	91.03	32.09	454.01	68.62	1156.37	104.29	0.393	6.374	12.703	114	113	133
ALIEAHFENRKKEEEELVS	5	541.5001	541.4994	0.0038	iTRAQ4plex@N-term; iTRAQ4plex(K)@11; iTRAQ4plex(K)@12	99	4.59E+03	1023.47	98.93	1410.69	118.05	989.5	103.43	1866.55	132.99	0.530	0.967	1.323	114	113	132
ALIEAHFENRKKEEEELV	4	654.8692	654.8644	0.0193	iTRAQ4plex@N-term; iTRAQ4plex(K)@11; iTRAQ4plex(K)@12	99	19394.16	3797.31	189.71	3605.42	193.05	5576.18	239.07	8879.23	290.77	0.628	1.468	2.463	114	113	131
ALIEAHFENRKKEEEEL	5	504.2808	504.2793	0.0076	iTRAQ4plex@N-term; iTRAQ4plex(K)@11; iTRAQ4plex(K)@12	99	7564.69	1284.72	110.57	1473.13	124.08	3330.39	182.89	4591.95	209.54	0.725	2.592	3.117	114	113	130
ALIEAHFENRKKEE	4	537.3061	537.3050	0.0045	iTRAQ4plex@N-term; iTRAQ4plex(K)@11; iTRAQ4plex(K)@12	99	45468.57	3359.27	178.69	3646.65	203.61	18604.8	428.82	24810.8	487.64	0.750	5.538	6.804	114	113	127
ALIEAHFENRKK	4	472.7841	472.7837	0.0017	iTRAQ4plex@N-term; iTRAQ4plex(K)@11; iTRAQ4plex(K)@12	99	9822.04	1116.53	102.94	1131.19	111.43	4217.33	204.49	5517.48	230.13	0.764	3.777	4.878	114	113	125
ALIEAHFENRKKEEEE	4	601.8303	601.8263	0.0162	iTRAQ4plex@N-term; iTRAQ4plex(K)@11; iTRAQ4plex(K)@12	99	6839.03	157.71	38.72	174.45	53.1	3576.78	186.79	4374.45	204.89	0.818	22.679	25.076	114	113	129
ALIEAHFENRK	3	539.3130	539.3101	0.0086	iTRAQ4plex@N-term; iTRAQ4plex(K)@11	98.1	28804.01	11722.3	333.48	11690.1	340.22	3089.46	191.86	3732.72	189.79	0.828	0.264	0.319	114	113	124
AHFENRKKEEEELVSLKDR	5	587.5330	587.5316	0.0069	iTRAQ4plex@N-term; iTRAQ4plex(K)@7; iTRAQ4plex(K)@8; iTRAQ4plex(K)@17	99	8352.32	1382.08	114.7	1600.09	126.71	1486.3	125.88	2982.83	168.02	0.498	1.075	1.864	118	117	136
AHFENRKKEEEELVSLKDRIE	5	635.9596	635.9570	0.0132	iTRAQ4plex@N-term; iTRAQ4plex(K)@7; iTRAQ4plex(K)@8; iTRAQ4plex(K)@17	99	6855.42	3741.85	188.44	3784.56	193.85	1415.86	127.73	2608.96	157.39	0.543	0.378	0.689	118	117	138
AHFENRKKEEEE	4	495.2642	495.2643	-0.0005	iTRAQ4plex@N-term; iTRAQ4plex(K)@7; iTRAQ4plex(K)@8	99	1928.45	177.01	40.98	177.14	51.47	2990.64	168.11	1334.6	116.91	2.241	16.895	7.534	118	117	129
FENRKKEEEELVSLKDRIE	5	594.3400	594.3378	0.0113	iTRAQ4plex@N-term; iTRAQ4plex(K)@5; iTRAQ4plex(K)@6; iTRAQ4plex(K)@15	99	8848.17	1879.12	133.4	1711.91	132.15	1775.51	136.03	2770.6	162.48	0.641	0.945	1.618	120	119	138
FENRKKEEEELVSLKDR	5	545.9138	545.9124	0.0070	iTRAQ4plex@N-term; iTRAQ4plex(K)@5; iTRAQ4plex(K)@6; iTRAQ4plex(K)@15	99	16611.93	1987.89	137.42	2112.73	151.11	6880.48	262.45	10253.4	312.61	0.671	3.461	4.853	120	119	136
FENRKKEEEELV	3	661.3749	661.3688	0.0182	iTRAQ4plex@N-term; iTRAQ4plex(K)@5; iTRAQ4plex(K)@6	99	2178.59	308.23	54.03	281.09	55.22	898.72	92.88	405.54	64.41	2.216	2.916	1.443	120	119	131
ENRKKEEEELVSLKDRIE	5	564.9261	564.9241	0.0101	iTRAQ4plex@N-term; iTRAQ4plex(K)@4; iTRAQ4plex(K)@5; iTRAQ4plex(K)@14	99	63805.47	10740.5	318.83	9496.02	308.89	4673.73	225.42	5280.88	225.7	0.885	0.435	0.556	121	120	138
ENRKKEEEELVSLKDR	5	516.4998	516.4987	0.0054	iTRAQ4plex@N-term; iTRAQ4plex(K)@4; iTRAQ4plex(K)@5; iTRAQ4plex(K)@14	99	88425.84	3655.1	185.98	3211.83	182.78	5212.03	228.48	5473.76	230.05	0.952	1.426	1.704	121	120	136
NRKKEEEELVSLKDR	5	490.6910	490.6902	0.0039	iTRAQ4plex@N-term; iTRAQ4plex(K)@3; iTRAQ4plex(K)@4; iTRAQ4plex(K)@13	99	16431.43	3118.9	171.85	2821.26	173.24	6908.89	261.04	6699.31	254.88	1.031	2.215	2.375	122	121	136
NRKKEEEELVSLKDRIE	5	539.1176	539.1155	0.0103	iTRAQ4plex@N-term; iTRAQ4plex(K)@3; iTRAQ4plex(K)@4; iTRAQ4plex(K)@13	99	10888.5	3316.57	177.12	2846.64	170.24	2453.11	158.85	2282.94	149.08	1.075	0.740	0.802	122	121	138
RKKEEEELVSLKD	4	545.5770	545.5750	0.0083	iTRAQ4plex@N-term; iTRAQ4plex(K)@2; iTRAQ4plex(K)@3; iTRAQ4plex(K)@12	97.5	1490.62	103.98	31.46	120.25	37.14	673.73	80.98	663.34	80.18	1.016	6.479	5.516	123	122	135

RKKEEEELVSLKDRIE	5	516.3077	516.3069	0.0035	iTRAQ4plex@N-term; iTRAQ4plex(K)@2; iTRAQ4plex(K)@3; iTRAQ4plex(K)@12	99	83561.24	3171.7	173.55	3323.81	211.27	39231.9	614.44	33038.9	568.91	1.187	12.369	9.940	123	122	138
RKKEEEELVSLKDR	5	467.8816	467.8817	-0.0002	iTRAQ4plex@N-term; iTRAQ4plex(K)@2; iTRAQ4plex(K)@3; iTRAQ4plex(K)@12	99	122107.5	924.2	93.21	521.53	125.48	33703.1	568.19	28008	523.13	1.203	36.467	53.703	123	122	136
KEEEELVSLKDRIE	4	538.0616	538.0574	0.0169	iTRAQ4plex@N-term; iTRAQ4plex(K)@1; iTRAQ4plex(K)@10	99	117064.1	52638.9	707.1	55519.1	738.42	6181.1	303.24	6809.23	257.33	0.908	0.117	0.123	125	124	138
KEEEELVSLKDR	3	636.3703	636.3652	0.0154	iTRAQ4plex@N-term; iTRAQ4plex(K)@1; iTRAQ4plex(K)@10	99	37864.92	12769.2	347.89	12172.9	348.55	5253.99	240.34	5403.44	228.88	0.972	0.411	0.444	125	124	136
KEEEELVSLKDRIER	4	577.0847	577.0826	0.0083	iTRAQ4plex@N-term; iTRAQ4plex(K)@1; iTRAQ4plex(K)@10	99	661.22	304.61	53.84	350.17	58.64	91.09	32.86	90.47	29.66	1.007	0.299	0.258	125	124	139
KEEEELVSLKD	3	584.3331	584.3315	0.0050	iTRAQ4plex@N-term; iTRAQ4plex(K)@1; iTRAQ4plex(K)@10	98.5	4657.49	3213.66	174.76	3471.07	184.74	666.34	91.69	612.55	77.38	1.088	0.207	0.176	125	124	135
EEEELVSLKDRIE	3	626.3433	626.3417	0.0048	iTRAQ4plex@N-term; iTRAQ4plex(K)@9	99	6283.56	856.91	90.1	796.84	91.37	1574.69	125.28	1716.67	128.72	0.917	1.838	2.154	126	125	138
EEEELVSLKDR	3	545.6334	545.6328	0.0018	iTRAQ4plex@N-term; iTRAQ4plex(K)@9	99	13742.55	1480.09	118.43	1397.14	127.69	8187.63	282.55	8486.31	286.4	0.965	5.532	6.074	126	125	136
EELVSLKDRIE	3	540.3158	540.3133	0.0074	iTRAQ4plex@N-term; iTRAQ4plex(K)@7	99	53994.85	12597.3	345.6	12179.8	355.55	21454.6	460.13	16171.3	399.49	1.327	1.703	1.328	128	127	138
LVSLKDRIERRRAERAE	4	597.1085	597.1064	0.0082	iTRAQ4plex@N-term; iTRAQ4plex(K)@5	99	8144.67	233.79	47.22	290.54	56.65	1218.47	111.43	2485.63	153.35	0.490	5.212	8.555	130	129	146
SLKDRIERRRAERAE	3	725.0921	725.0887	0.0102	iTRAQ4plex@N-term; iTRAQ4plex(K)@3	99	2615.9	97.34	30.49	128.74	35.49	45.32	22.8	68.06	25.49	0.666	0.466	0.529	132	131	146
QQRIRNEREKERQNRLAEER	5	586.1294	586.1265	0.0145	iTRAQ4plex@N-term; iTRAQ4plex(K)@10	99	12731.77	2006.26	138.07	2156.55	146.27	996.57	104.63	1234.93	108.93	0.807	0.497	0.573	147	146	166
QQRIRNEREKERQNRLA	4	628.8620	628.8597	0.0091	iTRAQ4plex@N-term; iTRAQ4plex(K)@10	99	30608	4051.84	196.03	3992.15	200.59	3290.37	183.58	2476.49	156.17	1.329	0.812	0.620	147	146	163
QQRIRNEREKERQNRLAEE	5	554.9083	554.9063	0.0102	iTRAQ4plex@N-term; iTRAQ4plex(K)@10	99	14550.89	987.24	96.73	943.2	97.97	1014.62	101.08	742.45	85.54	1.367	1.028	0.787	147	146	165
RIRNEREKERQNRLA	4	564.8325	564.8304	0.0083	iTRAQ4plex@N-term; iTRAQ4plex(K)@8	97.8	7680.35	767.48	85.25	699.07	84.55	800.26	89.37	472.22	68.77	1.695	1.043	0.675	149	148	163
NEREKERQNRLAEE	4	523.0300	523.0302	-0.0006	iTRAQ4plex@N-term; iTRAQ4plex(K)@5	98.7	4335.77	1481.9	118.47	1364.73	118.3	1736.75	131.02	821.49	91.53	2.114	1.172	0.602	152	151	165
RLAEERARREE	3	520.2885	520.2892	-0.0020	iTRAQ4plex@N-term	97.4	396.59	50.2	21.91	68.5	26.45	125.42	34.92	54.4	23.66	2.306	2.498	0.794	161	160	171
EERARREEEENRRKAEDEARKKKALSNM	7	597.9077	597.9024	0.0371	iTRAQ4plex@N-term; iTRAQ4plex(K)@14; iTRAQ4plex(K)@21; iTRAQ4plex(K)@22; iTRAQ4plex(K)@23	99	17684.76	6076.24	240.03	5906.64	244.55	5611.98	237.89	3315.7	182.2	1.693	0.924	0.561	164	163	191
EERARREEEENRRKAEDEARKKKALS	6	656.5453	656.5377	0.0455	iTRAQ4plex@N-term; iTRAQ4plex(K)@14; iTRAQ4plex(K)@21; iTRAQ4plex(K)@22; iTRAQ4plex(K)@23	99	5344.19	2063.67	139.73	1793.64	135.44	1888.18	137.53	1068.67	103.57	1.767	0.915	0.596	164	163	189
ARREEEENRRKAEDE	4	552.0360	552.0357	0.0012	iTRAQ4plex@N-term; iTRAQ4plex(K)@11	99	1026.69	235.84	47.22	194.56	44.27	81.81	29.83	51.02	22.58	1.603	0.347	0.262	167	166	181
ARREEEENRRKAEDEARKKKALSNM	6	628.3595	628.3539	0.0337	iTRAQ4plex@N-term; iTRAQ4plex(K)@11; iTRAQ4plex(K)@18; iTRAQ4plex(K)@19; iTRAQ4plex(K)@20	99	31901.04	5235.48	222.54	4509.07	213.53	2954.13	174.82	1610.99	127.54	1.834	0.564	0.357	167	166	191
ARREEEENRRKAE	4	491.0177	491.0183	-0.0025	iTRAQ4plex@N-term; iTRAQ4plex(K)@11	98.1	1415.64	740.61	83.71	648.23	80.55	228.89	50.39	118.18	34.64	1.937	0.309	0.182	167	166	179
EENRRKAEDEARKKKALSNM	5	625.5623	625.5583	0.0201	iTRAQ4plex@N-term; iTRAQ4plex(K)@6; iTRAQ4plex(K)@13; iTRAQ4plex(K)@14; iTRAQ4plex(K)@15	99	43464.21	6023.98	238.65	5022.47	225.91	3696.48	195.6	2954.07	170.25	1.251	0.614	0.588	172	171	191

EENRRKAEDEARKKKALS	5	576.5446	576.5416	0.0150	iTRAQ4plex@N-term; iTRAQ4plex(K)@6; iTRAQ4plex(K)@13; iTRAQ4plex(K)@14; iTRAQ4plex(K)@15	98.7	5250.57	3131.44	171.76	2089.57	146.95	1526.36	125.08	784.31	89.15	1.946	0.487	0.375	172	171	189
NMMHFGGYIQKQAQ	3	647.6649	647.6620	0.0089	iTRAQ4plex@N-term; iTRAQ4plex(K)@11	99	1342.3	317.5	55.04	402.38	62.76	120.13	38.07	270.87	50.59	0.443	0.378	0.673	190	189	203
NMMHFGGYIQKQA	3	604.9783	604.9758	0.0075	iTRAQ4plex@N-term; iTRAQ4plex(K)@11	99	606.89	125.88	34.78	202.12	44.6	156.19	41.1	327.8	55.67	0.476	1.241	1.622	190	189	202
NMMHFGGYIQK	3	538.6112	538.6105	0.0020	iTRAQ4plex@N-term; iTRAQ4plex(K)@11	99	2636.02	677.42	80.3	783.07	88.42	614.81	80.36	817.65	88.47	0.752	0.908	1.044	190	189	200
NMMHFGGYIQ	2	671.3157	671.3137	0.0040	iTRAQ4plex@N-term	99	1220.73	191.21	42.42	118.76	35.35	131.45	36.74	162.54	39.49	0.809	0.687	1.369	190	189	199
NMMHFGGYIQKQAQTER	4	582.5488	582.5461	0.0108	iTRAQ4plex@N-term; iTRAQ4plex(K)@11	97.5	646.29	276.21	51.18	270.2	51.95	138.16	38.72	166.39	39.99	0.830	0.500	0.616	190	189	206
MMHFGGYIQK	3	500.5972	500.5962	0.0029	iTRAQ4plex@N-term; iTRAQ4plex(K)@10	99	485.05	199.99	43.58	209.25	45.82	161.34	41.23	220.19	45.93	0.733	0.807	1.052	191	190	200
MHFGGYIQKQAQTERK	5	471.6621	471.6611	0.0051	iTRAQ4plex@N-term; iTRAQ4plex(K)@9; iTRAQ4plex(K)@16	99	40508.85	18502.2	421.23	28028.4	519.17	0	254.9	6785.51	250.68	0.000	0.000	0.242	192	191	207
MHFGGYIQKQAQTER	4	521.2767	521.2753	0.0056	iTRAQ4plex@N-term; iTRAQ4plex(K)@9	99	27889.9	13374.1	357.44	17759.1	414.48	65.95	106.57	1345.03	112.32	0.049	0.005	0.076	192	191	206
MHFGGYIQKQAQTE	3	642.6674	642.6642	0.0095	iTRAQ4plex@N-term; iTRAQ4plex(K)@9	99	1883.29	632.32	77.51	678.65	82.02	286.49	57.61	700.32	81.23	0.409	0.453	1.032	192	191	205
MHFGGYIQKQAQ	3	565.9691	565.9675	0.0049	iTRAQ4plex@N-term; iTRAQ4plex(K)@9	99	100943.2	28427.8	519.21	27640.2	528.18	22325.7	491.02	47093.6	668.45	0.474	0.785	1.704	192	191	203
MHFGGYIQKQAQT	3	599.6514	599.6500	0.0041	iTRAQ4plex@N-term; iTRAQ4plex(K)@9	99	1890.4	953.64	95.22	1054.11	101.97	339.38	63.16	611.83	76.21	0.555	0.356	0.580	192	191	204
MHFGGYIQK	3	456.9139	456.9161	-0.0065	iTRAQ4plex@N-term; iTRAQ4plex(K)@9	99	101815.9	45601.8	657.85	46041.5	678.79	28985.5	554.53	32679.3	562.58	0.887	0.636	0.710	192	191	200
MHFGGYIQKQ	3	499.6017	499.6023	-0.0016	iTRAQ4plex@N-term; iTRAQ4plex(K)@9	99	8810.56	4478.72	205.93	4064.99	202.18	2341.36	158.12	2525.75	156.36	0.927	0.523	0.621	192	191	201
HFGGYIQKQAQTERK	5	445.4520	445.4530	-0.0050	iTRAQ4plex@N-term; iTRAQ4plex(K)@8; iTRAQ4plex(K)@15	99	7176.65	1246.62	109.25	1793.21	132.31	639.52	90.49	3130.33	170.98	0.204	0.513	1.746	193	192	207
HFGGYIQKQAQTER	4	488.5160	488.5152	0.0033	iTRAQ4plex@N-term; iTRAQ4plex(K)@8	99	2810.78	527.78	71.12	783.53	87.22	204.77	50.49	521.7	70.14	0.393	0.388	0.666	193	192	206
HFGGYIQKQAQ	3	522.2875	522.2874	0.0005	iTRAQ4plex@N-term; iTRAQ4plex(K)@8	99	21667.15	372.02	59.41	370.04	61.39	441.18	66.32	272.37	52.15	1.620	1.186	0.736	193	192	203
FGGYIQKQAQTERK	4	522.3005	522.2997	0.0032	iTRAQ4plex@N-term; iTRAQ4plex(K)@7; iTRAQ4plex(K)@14	99	3981.79	1726.7	127.89	1591.03	126.12	611.23	86.47	2267.09	145.65	0.270	0.354	1.425	194	193	207
FGGYIQKQAQTER	3	605.3334	605.3315	0.0057	iTRAQ4plex@N-term; iTRAQ4plex(K)@7	99	1499.53	880.15	91.53	1017.48	99.79	148.97	46.36	465.2	66.1	0.320	0.169	0.457	194	193	206
FGGYIQKQAQ	2	714.3997	714.3979	0.0037	iTRAQ4plex@N-term; iTRAQ4plex(K)@7	99	699.33	185.24	41.83	145.63	39.49	339.75	58.39	463.92	66.63	0.732	1.834	3.186	194	193	203
GGYIQKQAQTERK	4	485.5324	485.5326	-0.0007	iTRAQ4plex@N-term; iTRAQ4plex(K)@6; iTRAQ4plex(K)@13	99	11648.24	7754.45	271.16	7556.03	274.39	3081.21	188.63	6833.52	254.05	0.451	0.397	0.904	195	194	207
GGYIQKQAQTER	3	556.3112	556.3087	0.0076	iTRAQ4plex@N-term; iTRAQ4plex(K)@6	99	4315.25	2700.7	159.91	2446.15	156.15	688.75	90.93	1205.26	107.04	0.571	0.255	0.493	195	194	206
GGYIQKQAQTE	3	504.2747	504.2750	-0.0009	iTRAQ4plex@N-term; iTRAQ4plex(K)@6	99	468.66	317.22	54.86	314.27	56.29	260.15	52.43	409.57	62.44	0.635	0.820	1.303	195	194	205
GGYIQKQAQ	3	427.5771	427.5782	-0.0034	iTRAQ4plex@N-term; iTRAQ4plex(K)@6	99	13130.57	3309.68	177.01	2952.92	177.54	7523.57	271.68	6482.17	251.55	1.161	2.273	2.195	195	194	203
GYIQKQAQTER	3	537.3016	537.3015	0.0002	iTRAQ4plex@N-term; iTRAQ4plex(K)@5	99	1365.94	503.42	69.2	566.34	75.16	388.41	65.41	909.73	92.62	0.427	0.772	1.606	196	195	206
GYIQKQAQTERK	4	471.2771	471.2772	-0.0005	iTRAQ4plex@N-term; iTRAQ4plex(K)@5; iTRAQ4plex(K)@12	98.6	5386.18	4506.47	206.8	4572.06	213.44	2244.2	157.77	3782.8	189.63	0.593	0.498	0.827	196	195	207
KQAQTERKSGKRQTE	5	471.0765	471.0779	-0.0067	iTRAQ4plex@N-term; iTRAQ4plex(K)@1; iTRAQ4plex(K)@8; iTRAQ4plex(K)@11	99	29325.24	18581.1	419.75	18094.8	435.31	37526.5	608.02	31382	554.23	1.196	2.020	1.734	200	199	214
KQAQTERKSGKRQ	5	425.0575	425.0598	-0.0114	iTRAQ4plex@N-term; iTRAQ4plex(K)@1; iTRAQ4plex(K)@8; iTRAQ4plex(K)@11	99	6274.41	6321.52	244.85	6217.06	248.24	1711.12	141.26	1353.81	115.51	1.264	0.271	0.218	200	199	212

QAQTERKSGKRQTE	4	520.5459	520.5462	-0.0013	iTRAQ4plex@N-term; iTRAQ4plex(K)@7; iTRAQ4plex(K)@10	98.2	8637.16	3756.78	188.86	3894.51	198.42	3947	200.1	3309.58	179.9	1.193	1.051	0.850	201	200	214
AQTERKSGKRQTE	4	488.5305	488.5316	-0.0044	iTRAQ4plex@N-term; iTRAQ4plex(K)@6; iTRAQ4plex(K)@9	99	3453.41	1600.08	123.19	1576.97	125	422.47	70.49	378.25	60.82	1.117	0.264	0.240	202	201	214
REKKKKILAER	4	530.6071	530.6056	0.0060	iTRAQ4plex@N-term; iTRAQ4plex(K)@3; iTRAQ4plex(K)@4; iTRAQ4plex(K)@5; iTRAQ4plex(K)@6	99	21392.55	3554.44	183.36	3042.6	174.62	1030.29	108.24	1063.14	101.53	0.969	0.290	0.349	215	214	225
AERRKVLAIDHLNEDQLRE	5	519.4965	519.4941	0.0123	iTRAQ4plex@N-term; iTRAQ4plex(K)@5	99	2478.49	265.04	50.09	237.36	48.98	163.75	42.42	371.91	59.24	0.440	0.618	1.567	223	222	241
AERRKVLAIDHLNED	4	517.5455	517.5442	0.0052	iTRAQ4plex@N-term; iTRAQ4plex(K)@5	99	1805	280.98	51.66	298.39	54.49	158.11	41.71	265.93	50.29	0.595	0.563	0.891	223	222	237
AERRKVLAIDHLNEDQLR	5	493.6863	493.6855	0.0038	iTRAQ4plex@N-term; iTRAQ4plex(K)@5	99	2731.58	390.69	61.06	500.22	70.7	484.99	71.05	701.85	81.84	0.691	1.241	1.403	223	222	240
ERRKVLAIDHLNEDQLR	5	479.4785	479.4781	0.0018	iTRAQ4plex@N-term; iTRAQ4plex(K)@4	99	3702.06	691.66	80.98	673.12	82.81	757.87	88.64	1166.19	105.39	0.650	1.096	1.733	224	223	240
RKVLAIDHLNEDQLREKAKELW	6	547.6580	547.6556	0.0142	iTRAQ4plex@N-term; iTRAQ4plex(K)@2; iTRAQ4plex(K)@17; iTRAQ4plex(K)@19	98.5	3169.36	1278.28	110.53	1722.45	129.25	175.16	53	503.69	68.92	0.348	0.137	0.292	226	225	247
RKVLAIDHLNED	4	428.4990	428.4990	0.0001	iTRAQ4plex@N-term; iTRAQ4plex(K)@2	98	2406.5	651	78.41	511.78	71.68	103.36	36.05	113.42	33.16	0.911	0.159	0.222	226	225	237
RKVLAIDHLNEDQLRE	4	560.0710	560.0706	0.0017	iTRAQ4plex@N-term; iTRAQ4plex(K)@2	99	3048.33	736.61	83.59	733.94	86.01	616.49	79.8	619.86	77.52	0.995	0.837	0.845	226	225	241
RKVLAIDHLNEDQLR	5	422.4496	422.4494	0.0010	iTRAQ4plex@N-term; iTRAQ4plex(K)@2	99	9628.12	789.27	86.36	638.81	80.36	318.19	58.47	266.17	51.09	1.195	0.403	0.417	226	225	240
KVLAIDHLNEDQLR	4	488.7858	488.7846	0.0046	iTRAQ4plex@N-term; iTRAQ4plex(K)@1	99	1772.86	588.97	74.55	442.71	68.03	634.13	79.74	602.34	76.47	1.053	1.077	1.361	227	226	240
AIDHLNEDQLREKAKELW	5	528.8951	528.8952	-0.0006	iTRAQ4plex@N-term; iTRAQ4plex(K)@13; iTRAQ4plex(K)@15	99	1819.72	486.62	68.11	596.74	76.87	366.52	62.68	498.87	69.13	0.735	0.753	0.836	230	229	247
AIDHLNEDQLRE	3	532.9423	532.9415	0.0026	iTRAQ4plex@N-term	99	2596.32	335.87	56.74	499.3	70.27	430.52	66.55	414.48	63.48	1.039	1.282	0.830	230	229	241
AIDHLNEDQLR	3	489.9268	489.9272	-0.0014	iTRAQ4plex@N-term	99	5857.02	1134.62	103.67	1047.49	104.48	1897.55	136.58	1293.72	113.17	1.467	1.672	1.235	230	229	240
IDHLNEDQLREKAKELW	4	643.1116	643.1079	0.0146	iTRAQ4plex@N-term; iTRAQ4plex(K)@12; iTRAQ4plex(K)@14	99	122338.1	32743.1	558.08	36790.6	607.32	31483.9	579.7	59942	755.87	0.525	0.962	1.629	231	230	247
IDHLNEDQLRE	3	509.2628	509.2624	0.0011	iTRAQ4plex@N-term	99	117630.5	3307.6	176.81	2713.16	170.59	6883.5	259.25	5103.38	224.18	1.349	2.081	1.881	231	230	241
IDHLNEDQLREKA	4	468.0063	468.0072	-0.0035	iTRAQ4plex@N-term; iTRAQ4plex(K)@12	98.1	25598.88	631.1	77.45	686.02	84.13	1222.15	109.76	901.86	94.29	1.355	1.937	1.315	231	230	243
IDHLNEDQLREKAKE	4	568.3185	568.3171	0.0056	iTRAQ4plex@N-term; iTRAQ4plex(K)@12; iTRAQ4plex(K)@14	98.7	659.89	352.55	57.7	274.44	53.75	484.83	69.13	348.65	58.64	1.391	1.375	1.270	231	230	245
IDHLNEDQLR	2	698.8740	698.8687	0.0107	iTRAQ4plex@N-term	99	16758.9	1392.16	114.96	1426.46	125.96	6156.51	243.13	3544.15	188.51	1.737	4.422	2.485	231	230	240
IDHLNEDQLREKAK	5	429.0444	429.0466	-0.0110	iTRAQ4plex@N-term; iTRAQ4plex(K)@12; iTRAQ4plex(K)@14	99	9920.18	2183.01	143.71	1900.24	144.89	7108.04	261.5	4036.24	201.31	1.761	3.256	2.124	231	230	244
HLNEDQLREKAKELW	4	586.0827	586.0801	0.0102	iTRAQ4plex@N-term; iTRAQ4plex(K)@10; iTRAQ4plex(K)@12	98.4	23053.79	6665.51	252.13	8325.26	286.21	3748.38	202.6	4074.81	198.51	0.920	0.562	0.489	233	232	247
HLNEDQLR	2	584.8118	584.8132	-0.0027	iTRAQ4plex@N-term	99	593.62	283.92	52.05	362.32	60.16	351.15	59.74	292.12	53.47	1.202	1.237	0.806	233	232	240
LNEDQLREKAKELW	4	551.8160	551.8154	0.0022	iTRAQ4plex@N-term; iTRAQ4plex(K)@9; iTRAQ4plex(K)@11	97.6	26519.26	9872.12	306.42	11037.9	331.36	6855.32	269.18	6896.06	258.55	0.994	0.694	0.625	234	233	247
EDQLREKAKELW	3	659.7123	659.7092	0.0094	iTRAQ4plex@N-term; iTRAQ4plex(K)@7; iTRAQ4plex(K)@9	99	22120.42	9493.87	300.51	10640.9	323.54	2884.91	184.12	2762.1	164.04	1.044	0.304	0.260	236	235	247
QSIYNLEAEKFDLQE	3	705.7053	705.6998	0.0166	iTRAQ4plex@N-term; iTRAQ4plex(K)@10	99	12885.1	4241.93	204.23	11553.8	330.3	0	112.31	939.23	93.53	0.000	0.000	0.081	248	247	262
AEKFDLQEKFKQQKYEINVLR	6	563.3301	563.3295	0.0037	iTRAQ4plex@N-term; iTRAQ4plex(K)@3; iTRAQ4plex(K)@9; iTRAQ4plex(K)@11; iTRAQ4plex(K)@14	98.7	3142.72	536.4	75.69	3732.9	186.57	132.55	60.74	910.32	92.18	0.146	0.247	0.244	255	254	275

AEKFDLQEKFKQQKYE	5	556.7198	556.7178	0.0100	iTRAQ4plex@N-term; iTRAQ4plex(K)@3; iTRAQ4plex(K)@9; iTRAQ4plex(K)@11; iTRAQ4plex(K)@14	99	15677.12	3397.89	180.45	5015.96	222.72	3975.62	211.2	12523.4	342.87	0.317	1.170	2.497	255	254	270
AEKFDLQEKFKQQKY	4	663.3879	663.3848	0.0125	iTRAQ4plex@N-term; iTRAQ4plex(K)@3; iTRAQ4plex(K)@9; iTRAQ4plex(K)@11; iTRAQ4plex(K)@14	99	5566.45	923.58	94.48	1750.51	130.06	664.62	88.76	1850.89	131.94	0.359	0.720	1.057	255	254	269
AEKFDLQEKFKQQK	4	622.6217	622.6189	0.0111	iTRAQ4plex@N-term; iTRAQ4plex(K)@3; iTRAQ4plex(K)@9; iTRAQ4plex(K)@11; iTRAQ4plex(K)@14	99	65836.44	16040.9	392.02	23474.3	485.11	28810.5	551.88	62465.8	768.22	0.461	1.796	2.661	255	254	268
AEKFDLQEKFKQQ	4	554.5710	554.5696	0.0053	iTRAQ4plex@N-term; iTRAQ4plex(K)@3; iTRAQ4plex(K)@9; iTRAQ4plex(K)@11	99	3350.39	1316.29	111.83	1409.29	119.52	1586.9	129.05	2954.8	167.42	0.537	1.206	2.097	255	254	267
AEKFDLQEKFK	4	490.5421	490.5404	0.0069	iTRAQ4plex@N-term; iTRAQ4plex(K)@3; iTRAQ4plex(K)@9; iTRAQ4plex(K)@11	99	162509.5	27234	507.78	24587.4	495.58	8590.58	311.42	8302.84	284.84	1.035	0.315	0.338	255	254	265
AEKFDLQEKF	3	562.9880	562.9857	0.0067	iTRAQ4plex@N-term; iTRAQ4plex(K)@3; iTRAQ4plex(K)@9	99	85388.65	1413.82	115.78	1378.77	122.95	5040.61	221.96	4762.4	215.18	1.058	3.565	3.454	255	254	264
FDLQEKFKQQK	4	504.5506	504.5497	0.0034	iTRAQ4plex@N-term; iTRAQ4plex(K)@6; iTRAQ4plex(K)@8; iTRAQ4plex(K)@11	98.8	19653.49	6328.11	244.87	5961.49	256.05	22035.1	464.17	21131.8	453.13	1.043	3.482	3.545	258	257	268
QEKFKQQKYEINVLR	5	506.3024	506.3027	-0.0013	iTRAQ4plex@N-term; iTRAQ4plex(K)@3; iTRAQ4plex(K)@5; iTRAQ4plex(K)@8	99	3217.42	666.44	79.68	790.34	90.93	1835.21	136.62	3142.12	172.72	0.584	2.754	3.976	261	260	275
EKFKQQKYEINVLR	5	480.6904	480.6909	-0.0028	iTRAQ4plex@N-term; iTRAQ4plex(K)@2; iTRAQ4plex(K)@4; iTRAQ4plex(K)@7	99	4419.8	1455.43	117.67	1646.33	129.83	2553.53	161.62	3961.02	194.22	0.645	1.754	2.406	262	261	275
KFKQQKYEINVLR	4	568.3528	568.3512	0.0063	iTRAQ4plex@N-term; iTRAQ4plex(K)@1; iTRAQ4plex(K)@3; iTRAQ4plex(K)@6	99	53105.56	17747.8	411.3	21697.8	463.21	12322.1	366.03	19149.3	427.42	0.643	0.694	0.883	263	262	275
KFKQQKYEINVL	3	705.4359	705.4322	0.0112	iTRAQ4plex@N-term; iTRAQ4plex(K)@1; iTRAQ4plex(K)@3; iTRAQ4plex(K)@6	99	2285.43	510.56	69.63	532.18	73.13	434.19	67.67	649.76	78.73	0.668	0.850	1.221	263	262	274
FKQQKYEINVLR	4	500.3023	500.3020	0.0014	iTRAQ4plex@N-term; iTRAQ4plex(K)@2; iTRAQ4plex(K)@5	98.2	1290.51	660.79	79.11	602.53	77.83	351.9	61.06	319.74	55.86	1.101	0.533	0.531	264	263	275
KQQKYEINVLR	4	463.5328	463.5349	-0.0082	iTRAQ4plex@N-term; iTRAQ4plex(K)@1; iTRAQ4plex(K)@4	99	13572.72	2059.46	140.09	2489.28	162.06	6644.8	257.35	8542.52	286.09	0.778	3.226	3.432	265	264	275
QQKYEINVLR	3	526.9798	526.9784	0.0043	iTRAQ4plex@N-term; iTRAQ4plex(K)@3	99	94941.23	5309.37	224.49	5417.13	231.33	1262.33	124.68	1867.01	133.81	0.676	0.238	0.345	266	265	275
QQKYEINVLRN	3	564.9953	564.9927	0.0078	iTRAQ4plex@N-term; iTRAQ4plex(K)@3	99	4782.05	2989.16	168.41	2998.2	171.85	329.95	70.35	406.26	62.68	0.812	0.110	0.136	266	265	276
YEINVLRNRINDNQ	3	635.6777	635.6735	0.0126	iTRAQ4plex@N-term	99	39575.43	14281.6	369.26	18531.8	424.73	2952.02	203.74	8952.15	290.3	0.330	0.207	0.483	269	268	282
YEINVLRNRINDNQK	4	545.0568	545.0562	0.0026	iTRAQ4plex@N-term; iTRAQ4plex(K)@15	99	13052.06	1558.93	121.8	1784.51	132.19	274.46	61.96	683.99	80.37	0.401	0.176	0.383	269	268	283
YEINVLRNRINDNQKVS	4	591.5833	591.5813	0.0081	iTRAQ4plex@N-term; iTRAQ4plex(K)@15	98.6	6122.79	960.63	97.45	2879.58	164.84	108.73	52.15	209.04	44.83	0.520	0.113	0.073	269	268	285
YEINVLRN	2	582.8285	582.8283	0.0004	iTRAQ4plex@N-term	99	19097.68	3013.5	169.12	3073.07	174.39	882.07	102.21	1296.91	111.41	0.680	0.293	0.422	269	268	276
RNRINDNQKVSKTRGKAK	5	567.5488	567.5491	-0.0014	iTRAQ4plex@N-term; iTRAQ4plex(K)@9; iTRAQ4plex(K)@12; iTRAQ4plex(K)@16; iTRAQ4plex(K)@18	99	5439.34	932.55	94.85	1683.78	130.63	3076.85	177.77	5546.87	229.4	0.555	3.299	3.294	275	274	292

RNRINDNQKVSKTRGK	5	498.9013	498.9023	-0.0048	iTRAQ4plex@N-term; iTRAQ4plex(K)@9; iTRAQ4plex(K)@12; iTRAQ4plex(K)@16	98.9	1457.28	332.95	56.47	484.64	69.78	633.94	80.86	1011.16	98.13	0.627	1.904	2.086	275	274	290
RNRINDNQKVSK	4	476.7828	476.7842	-0.0056	iTRAQ4plex@N-term; iTRAQ4plex(K)@9; iTRAQ4plex(K)@12	99	2545.92	742.33	84.19	966.34	98.83	1330.71	115.7	1345.98	114.19	0.989	1.793	1.393	275	274	286
NRINDNQKVSKTRGK	5	467.6804	467.6820	-0.0083	iTRAQ4plex@N-term; iTRAQ4plex(K)@8; iTRAQ4plex(K)@11; iTRAQ4plex(K)@15	99	11595.4	2747.27	162.4	4279.82	207.86	6618.56	260.81	11085	324.81	0.597	2.409	2.590	276	275	290
NRINDNQKVSKTRGKAK	5	536.3295	536.3289	0.0032	iTRAQ4plex@N-term; iTRAQ4plex(K)@8; iTRAQ4plex(K)@11; iTRAQ4plex(K)@15; iTRAQ4plex(K)@17	99	52590.34	9906.35	308.47	15753.7	401.46	31661.5	565.94	47028.9	671.26	0.673	3.196	2.985	276	275	292
NRINDNQKVSKTRGKAKVTG	5	587.7584	587.7564	0.0102	iTRAQ4plex@N-term; iTRAQ4plex(K)@8; iTRAQ4plex(K)@11; iTRAQ4plex(K)@15; iTRAQ4plex(K)@17	99	122105.4	14006.1	366.28	20423	466.6	66965.1	816.81	92213.3	944.56	0.726	4.781	4.515	276	275	295
NRINDNQKVSKTRGKAKVT	5	576.3544	576.3521	0.0114	iTRAQ4plex@N-term; iTRAQ4plex(K)@8; iTRAQ4plex(K)@11; iTRAQ4plex(K)@15; iTRAQ4plex(K)@17	99	73727.59	2227.43	146.06	3237.1	185.63	10489.9	322.44	13042.4	354.07	0.804	4.709	4.029	276	275	294
NRINDNQKVSK	4	437.7578	437.7589	-0.0045	iTRAQ4plex@N-term; iTRAQ4plex(K)@8; iTRAQ4plex(K)@11	99	8854.52	5790.24	234.61	6340.52	254.05	8397.63	292.05	10150.2	312.38	0.827	1.450	1.601	276	275	286
NRINDNQKVSKTR	5	401.8368	401.8383	-0.0078	iTRAQ4plex@N-term; iTRAQ4plex(K)@8; iTRAQ4plex(K)@11	97.2	25316	2025.18	139.19	2831.54	170.74	5892.92	241.96	6209.79	245.08	0.949	2.910	2.193	276	275	288
NRINDNQKVS	3	492.6094	492.6105	-0.0032	iTRAQ4plex@N-term; iTRAQ4plex(K)@8	99	275051.3	2499.51	154.22	2848.53	169.3	2949.37	172.14	1856.96	136	1.588	1.180	0.652	276	275	285
RINDNQKVSKTRGKAKVTG	5	564.9496	564.9478	0.0089	iTRAQ4plex@N-term; iTRAQ4plex(K)@7; iTRAQ4plex(K)@10; iTRAQ4plex(K)@14; iTRAQ4plex(K)@16	99	4379.85	1285.25	110.84	1750.57	131.78	1450.9	123.11	1869.8	133.94	0.776	1.129	1.068	277	276	295
RINDNQKVS	3	454.5946	454.5962	-0.0047	iTRAQ4plex@N-term; iTRAQ4plex(K)@7	99	15908.67	2988.76	168.17	2619.38	161.93	948.42	102.45	524.96	72.79	1.807	0.317	0.200	277	276	285
DNQKVSKTRGKAK	5	436.8748	436.8747	0.0006	iTRAQ4plex@N-term; iTRAQ4plex(K)@4; iTRAQ4plex(K)@7; iTRAQ4plex(K)@11; iTRAQ4plex(K)@13	99	658.48	434.78	64.41	554.9	74.76	681.06	83.11	743.9	84.72	0.916	1.566	1.341	280	279	292

Peptides were extracted from the distinct peptide list generated with ProteinPilot software by the following conditions; Accessions, P45389 TNNT2_human; "Best conf (Peptide)" > 96.8 (FDR < 1%); permitted modifications, iTRAQ at N-terminus and/or Lys, oxidation at Met, methylthio at Cys, PyroGlu at N-terminus; omit decoy sequence; quant annotation, Auto or Auto--shared MS/MS. The cleavage sites are indicated by the number of amino acid position N-terminus to the cleavage site.

Table S2 List of catalytic subunit of calpain-1 peptide generated by autolysis.

Sequence	Theor z	Obs m/z	Theor m/z	dMass	Modifications	Best Conf (Peptide)	Intensity (Peptide)	iTRAQ114 C2 40min Area 114	Err 114	iTRAQ115 C2 20min Area 115	Err 115	iTRAQ116 C1 40min Area 116	Err 116	iTRAQ117 C1 20min Area 117	Err 117	Start Position	cleavage sites N site	C site
EGFEDFTGGVT	2	651.8034	651.802124	0.00251227	iTRAQ4plex@N-term	97.4	232.75	52.01	22.36	83.98	28.81	77.74	28.28	83.92	28.46	212	211	222
GAKQVNYQGQM	3	504.6011	504.6008	0.00090373	iTRAQ4plex@N-term; iTRAQ4plex(K)@3	99	1524.83	103.65	31.62	184.83	44.15	583.33	76.21	786.17	86.71	278	277	288
GAKQVNYQGQMVN	3	575.64	575.63794	0.0062921	iTRAQ4plex@N-term; iTRAQ4plex(K)@3	99	1268.6	74.28	26.64	99.29	33.31	481.01	68.69	546.05	72.52	278	277	290
SSEWNGVDPYQRDQLR	3	698.679	698.675537	1.04E-02	iTRAQ4plex@N-term	99	2246.22	30.62	17.32	77.7	31.62	856.57	90.93	777.97	87	311	310	326
TLYEGTWRRGSTAGGCR	4	516.0027	516.002319	0.00143959	iTRAQ4plex@N-term; Methylthio(C)@16	99	30560.12	200.53	43.47	135.62	40.12	763.53	86.99	1087.78	101.92	369	368	385
YEGTWRRGSTAGGCR	4	462.4691	462.469391	-0.0012174	iTRAQ4plex@N-term; Methylthio(C)@14	99	1870.15	84.09	28.28	93.66	33.16	594.02	76.47	768.03	85.78	371	370	385
EGTWRRGSTAGGCR	4	421.7019	421.703552	-0.0066144	iTRAQ4plex@N-term; Methylthio(C)@13	98.5	9215.85	179.15	41.23	177.53	53.94	3779.2	190.5	3194.22	176.72	372	371	385
GCRNYPATF	2	609.7801	609.779419	0.00129446	iTRAQ4plex@N-term; Methylthio(C)@2	99	3651.48	31.78	17.32	23.45	20	530.84	71.68	553.32	73.14	383	382	391
KIRLEETDDPEDDYGGRESG	4	643.0651	643.06189	0.0130544	iTRAQ4plex@N-term; iTRAQ4plex(K)@1	99	18530.33	84.76	28.28	63.05	48.47	5647.78	230.55	2369.25	156.26	398	397	417
KIRLEETDDPEDDYGGRESGCS	4	702.0734	702.069092	0.0173239	iTRAQ4plex@N-term; iTRAQ4plex(K)@1; Methylthio(C)@21	99	8250.04	47.15	21.21	59.42	36.74	2547.83	154.74	934.88	98.88	398	397	419
KIRLEETDDPEDDYGGRESGCSF	4	738.8401	738.836182	0.0155603	iTRAQ4plex@N-term; iTRAQ4plex(K)@1; Methylthio(C)@21	99	778.08	45.52	20.73	35.54	20	146.26	37.68	119.41	34.2	398	397	420
KIRLEETDDPEDDYGGRESGCSFVL	4	791.8809	791.874329	2.64E-02	iTRAQ4plex@N-term; iTRAQ4plex(K)@1; Methylthio(C)@21	99	4486.98	10.55	10	9.67	16.12	534.97	71.47	403.06	62.92	398	397	422
KIRLEETDDPEDDYGGRESGCSFVLA	4	809.6402	809.633606	2.64E-02	iTRAQ4plex@N-term; iTRAQ4plex(K)@1; Methylthio(C)@21	98	2384.37	29.34	16.73	37.25	20.73	223.54	46.47	182.76	42.3	398	397	423
ETDDPEDDYGGRESGCSFVL	3	794.3335	794.327026	0.0193533	iTRAQ4plex@N-term; Methylthio(C)@16	99	508.9	7.34	8.37	9.08	10.95	104.36	31.46	51.2	22.8	403	402	422
EVPPELVGQPVH	3	482.2682	482.268738	-0.0016278	iTRAQ4plex@N-term	99	15905.55	127.8	34.64	63.02	44.83	4397	204.96	3386.03	182.45	448	447	459
EVPPELVGQPVHLK	3	610.6983	610.69574	0.00762692	iTRAQ4plex@N-term; iTRAQ4plex(K)@14	99	10107.98	234.5	47	157.81	53.66	4165.15	199.97	3553.67	186.32	448	447	461
EVPPELVGQPVHLKRDF	4	562.8215	562.822754	-0.0051386	iTRAQ4plex@N-term; iTRAQ4plex(K)@14	99	1064.42	365.03	58.81	344.82	59.07	296.91	55.31	290.45	53.1	448	447	464
EVPPELVGQPVHLKRDFFLA	4	645.6234	645.620178	1.30E-02	iTRAQ4plex@N-term; iTRAQ4plex(K)@14	98.6	429.57	13.58	11.4	19.2	14.83	112.69	33.01	94.21	30.33	448	447	467
LVGQPVHLK	3	426.9413	426.942719	-0.0042108	iTRAQ4plex@N-term; iTRAQ4plex(K)@9	99	3429.74	92.63	30	196.84	54.03	3410.04	180.64	2548.08	158.28	453	452	461
NASRARSEQFINLR	4	452.2516	452.25174	-0.0005704	iTRAQ4plex@N-term	99	21674.6	273.11	50.49	64.23	57.18	8178.66	279.44	6245.92	247.75	468	467	481
ASRARSEQFINLR	4	423.7404	423.740997	-2.44E-03	iTRAQ4plex@N-term	99	27558.99	248.78	48.06	0	45.08	6215.63	243.03	4115.01	201.95	469	468	481
RARSEQFINLR	3	511.9632	511.962524	2.02E-03	iTRAQ4plex@N-term	97.2	6096.15	477.49	67.59	671.12	82.45	1054.06	102.11	763.09	86.77	471	470	481
ARSEQFINLR	3	459.9256	459.928833	-0.0096664	iTRAQ4plex@N-term	97.6	640.6	283.65	52.05	372.93	60.65	229.75	48.98	156.39	39.36	472	471	481
SEQFINLREVS	2	733.394	733.391968	0.0039689	iTRAQ4plex@N-term	99	595.76	33.46	17.89	45.54	22.36	193.6	43.47	187.54	42.66	474	473	484
EVSTRFRLPPGEYVVVPST	3	759.7563	759.750183	0.0183638	iTRAQ4plex@N-term	99	10106.15	21.4	14.14	6.06	13.78	388.79	61.55	484.49	68.18	482	481	500
EVSTRFRLPPGEYVVVPSTF	3	808.7794	808.772949	1.93E-02	iTRAQ4plex@N-term	99	1682.09	3.08	5.48	7.13	9.49	72.61	26.45	62.58	24.69	482	481	501
EVSTRFRLPPGEYVVVPSTFEPNK	4	759.9219	759.915344	0.0260036	iTRAQ4plex@N-term; iTRAQ4plex(K)@24	99	547.65	19.27	13.41	4.84	8.94	76.11	27.38	109.01	32.24	482	481	505
RLPPGEYVVVPST	3	519.9634	519.96344	-1.20E-04	iTRAQ4plex@N-term	99	7710.44	281.29	51.57	232.42	48.57	154.77	40.74	235.63	47.43	488	487	500
RLPPGEYVVVPSTF	3	568.9886	568.986206	7.09E-03	iTRAQ4plex@N-term	99	3805.61	42.01	20	48.93	23.23	207.08	44.71	145.93	37.94	488	487	501
RLPPGEYVVVPSTFEPNK	4	580.077	580.075317	6.85E-03	iTRAQ4plex@N-term; iTRAQ4plex(K)@18	99	1843.37	99.97	30.82	106.44	33.91	339.12	58.9	687.4	80.61	488	487	505
VVVPSTFEPNK	3	502.2921	502.291962	4.50E-04	iTRAQ4plex@N-term; iTRAQ4plex(K)@11	98.9	1347.54	233.58	46.9	148.88	43.7	1303.63	111.38	691.2	83.48	495	494	505
EGDFVLRFF	2	637.3411	637.338501	0.00524028	iTRAQ4plex@N-term	99	13798.62	10.5	8.94	0	93.22	6020.09	238.67	3508.99	187.44	506	505	514
EGDFVLRFFSEKKA	4	527.05	527.049316	0.00260116	iTRAQ4plex@N-term; iTRAQ4plex(K)@12; iTRAQ4plex(K)@1	99	2435.59	10.47	10	13.44	26.45	1832.18	133.17	2007.2	139.08	506	505	519
SEKKAGTQELDDQVQ	4	527.7872	527.785339	0.00733037	iTRAQ4plex@N-term; iTRAQ4plex(K)@3; iTRAQ4plex(K)@4	99	704.6	250.07	48.57	183.1	44.83	552.62	74.02	665.76	79.99	515	514	529
SEKKAGTQELDDQVQAILPDE	4	687.3704	687.367249	1.28E-02	iTRAQ4plex@N-term; iTRAQ4plex(K)@3; iTRAQ4plex(K)@4	99	484.33	52.59	22.36	57.07	24.29	102.58	32.24	147.76	37.54	515	514	535
GTQELDDQVQ	2	638.8145	638.810486	0.00801637	iTRAQ4plex@N-term	99	534.48	35.04	18.16	22.34	15.81	75.3	27.01	52.56	22.8	520	519	529
GTQELDDQVQAILPDE	3	638.988	638.985291	0.00809806	iTRAQ4plex@N-term	99	597.43	86.85	28.63	65.21	26.07	84.44	29.66	153.85	38.21	520	519	535
QVLSEEEIDENF	2	798.387	798.38147	0.0110631	iTRAQ4plex@N-term	99	1615.54	9.6	9.49	3.96	10	182.79	42.54	296.55	53.1	536	535	547
RQLAGEDMEISVR	3	549.9589	549.958313	0.00175214	iTRAQ4plex@N-term	99	324.41	88.2	28.98	103.13	32.24	116.17	34.35	117.68	33.76	552	551	564
RQLAGEDMEISVRELR	4	512.2777	512.277527	7.77E-04	iTRAQ4plex@N-term	99	4537.53	142.59	36.74	126.82	37.41	455.54	68.03	867.3	90.6	552	551	567
SIILKALE	2	587.8889	587.886108	0.00563513	iTRAQ4plex@N-term; iTRAQ4plex(K)@5	97.2	455.14	3.98	6.32	15.03	14.14	190.78	42.89	171.65	40.86	236	235	243

Peptides were extracted from the distinct peptide list generated with ProteinPilot software by the following conditions; Accessions, P35750_CAN1_PIG; "Best conf (Peptide)" > 96.8 (FDR < 1%); permitted modifications; iTRAQ at N-terminus and/or Lys, oxidation at Met, methylthio at Cys, PyroGlu at N-termimus; ommit decoy sequence; quant annotation, Auto or Auto--shared MS/MS. The cleavage sites are indicated by the number of amino acid position N-terminus to the cleavage site.

Table S3 List of catalytic subunit of calpain-2 peptide generated by autolysis.

								iTRAQ114		iTRAQ115		iTRAQ116		iTRAQ117			cleavage sites	
Sequence	Theor z	Obs m/z	Theor m/z	dMass	Modifications	Best Conf (Peptide)	Intensity (Peptide)	C2 40min Area 114	Err 114	C2 20min Area 115	Err 115	C1 40min Area 116	Err 116	C1 20min Area 117	Err 117	Start Position	N site	C site
AGIAAKLAKDREAAEGLG	3	725.0975	725.0937	0.0114	iTRAQ4plex@N-term; iTRAQ4plex(K)@6; iTRAQ4plex(K)@9	99	575.54	301.86	53.66	378.8	60.65	14.81	20	112.46	32.4	2	1	19
QDPSFPAIPSALGFKEL	3	702.3951	702.3889	0.0186	iTRAQ4plex@N-term; iTRAQ4plex(K)@15	99	404.53	155.24	38.34	138.09	37.01	12.55	14.14	8.98	9.49	47	46	63
QDPSFPAIPSALGFKELGPYS	3	837.1227	837.1121	0.0318	iTRAQ4plex@N-term; iTRAQ4plex(K)@15	99	590.92	156.89	38.59	161.98	39.87	7	13.04	28.74	16.43	47	46	67
ADPQFIIGGATRTDICQ	3	666.0008	665.9965	0.0130	iTRAQ4plex@N-term; Methylthio(C)@16	99	479.19	130.63	35.07	82.35	28.98	8.35	11.83	35.21	18.16	83	82	99
NEEILARVVPLN	3	504.2960	504.2952	0.0025	iTRAQ4plex@N-term	99	367.05	131.48	35.35	142.17	37.41	32.83	20	36.11	18.71	117	116	128
EEILARVVPLN	3	466.2812	466.2809	0.0010	iTRAQ4plex@N-term	99	345.14	255.22	49.08	193.43	44.15	45.95	23.45	30.01	17.32	118	117	128
CPSWNTIDPEE	2	740.8185	740.8139	0.0092	iTRAQ4plex@N-term; Methylthio(C)@1	97.5	401.17	108.35	32.09	116.9	33.91	22.48	17.03	36.73	18.71	301	300	311
CPSWNTIDPEERERLT	3	712.6746	712.6705	0.0123	iTRAQ4plex@N-term; Methylthio(C)@1	97.7	1034.12	410.21	62.28	343.44	58.47	26.13	21.21	19.99	14.14	301	300	316
KMDGNWRRGSTAGGCR	4	522.2595	522.2578	0.0067	iTRAQ4plex@N-term; iTRAQ4plex(K)@1; Methylthio(C)@15	99	7969.86	3269.49	176.04	3129.68	175.59	76.3	50.69	117.43	33.91	360	359	375
MDGNWRRGSTAGGCR	4	454.2093	454.2086	0.0028	iTRAQ4plex@N-term; Methylthio(C)@14	99	32283.41	3624.3	185.5	3742.6	191.6	122.17	57.7	81.96	29.15	361	360	375
STAGGCRNYPNTF	3	526.5707	526.5701	0.0018	iTRAQ4plex@N-term; Methylthio(C)@6	99	2034.24	1043.17	99.43	989.01	98.88	113.4	41.23	193.76	43.01	369	368	381
GCRNYPNTF	2	631.2857	631.2823	0.0067	iTRAQ4plex@N-term; Methylthio(C)@2	97.7	4413.26	668.97	79.74	726.75	84.55	151.1	43.58	208.61	44.72	373	372	381
WMNPQYLIKLE	3	574.9904	574.9879	0.0076	iTRAQ4plex@N-term; iTRAQ4plex(K)@9	99	1269.93	662.2	79.42	789.41	87.62	9.37	23.45	20.33	14.14	382	381	392
LIKLEEEDEDEEDGESGCTF	3	874.3916	874.3835	0.0242	iTRAQ4plex@N-term; iTRAQ4plex(K)@3; Methylthio(C)@18	99	2589.97	370.16	59.41	456.2	66.55	2.45	17.03	11.29	10.49	388	387	407
EEDEDEEDGESGCTF	2	940.8442	940.8257	0.0368	iTRAQ4plex@N-term; Methylthio(C)@13	99	370.6	51.14	22.13	74.37	26.83	10.85	12.25	17.78	13.04	393	392	407
EEDEDEEDGESGCTFLVG	2	1075.4250	1075.4127	0.0254	iTRAQ4plex@N-term; Methylthio(C)@13	99	1027.24	44.5	20.73	82.91	28.1	0	5.41	6.67	7.74	393	392	410
HTIGFGIYEVPEELSGQT	3	707.6977	707.6922	0.0165	iTRAQ4plex@N-term	99	600.76	88.73	28.98	78.16	27.92	20.44	15.49	14.07	11.83	427	426	444
FGIYEVPEELSGQT	2	856.9360	856.9286	0.0147	iTRAQ4plex@N-term	99	1836.88	180.54	41.47	214.11	45.6	0	11.19	4.55	6.32	431	430	444
FGIYEVPEELSGQTNIH	3	693.0216	693.0168	0.0144	iTRAQ4plex@N-term	99	486.23	108.43	32.09	113.77	33.46	18.89	15.81	23.9	15.16	431	430	447
EVPEELSGQT	2	616.8135	616.8100	0.0070	iTRAQ4plex@N-term	99	19496.37	3703.43	187.67	4119.18	200.92	565.48	88.42	535.5	72.38	435	434	444
EVPEELSGQTN	2	673.8362	673.8314	0.0095	iTRAQ4plex@N-term	99	474.49	87.93	28.98	115.92	33.61	26.59	17.89	19.13	13.78	435	434	445
EVPEELSGQTNIH	3	532.9391	532.9377	0.0041	iTRAQ4plex@N-term	99	14240.96	2918.34	166.68	3377.17	181.41	78.8	53	330.62	55.85	435	434	447
EVPEELSGQTNIHL	3	570.6332	570.6324	0.0023	iTRAQ4plex@N-term	97.7	678.18	224.85	46.36	304.32	54.4	59.8	27.38	56.38	23.45	435	434	448
EVPEELSGQTNIHLS	3	599.6448	599.6431	0.0051	iTRAQ4plex@N-term	99	3614.22	954.95	95.17	942.5	96.53	184.64	48.26	192.54	43.24	435	434	449
ELSGQTNIH	2	571.8015	571.7997	0.0035	iTRAQ4plex@N-term	99	474.99	124.43	34.49	169.47	40.98	135.6	37.68	174.21	40.86	439	438	447
RARERSDTFINLR	3	593.3361	593.3349	0.0036	iTRAQ4plex@N-term	99	13938.38	1591.17	122.9	1640.91	127.06	212.1	54.67	198.85	44.16	457	456	469
SDTFINLR	2	555.3087	555.3072	0.0030	iTRAQ4plex@N-term	98.9	1243.84	626.68	77.13	637.37	79.36	150.27	43.01	230.2	46.9	462	461	469
EVLNRFKLPPGEYILVPST	4	615.8612	615.8581	0.0126	iTRAQ4plex@N-term; iTRAQ4plex(K)@7	99	4518.47	543.41	71.82	558.86	74.02	3.57	18.97	7.9	8.94	470	469	488
RFKLPPGEYILVPST	3	669.0674	669.0623	0.0152	iTRAQ4plex@N-term; iTRAQ4plex(K)@3	99	5457.06	501.21	68.91	469	68.11	50.85	27.38	18.65	14.14	474	473	488
RFKLPPGEYILVPSTF	3	718.0912	718.0851	0.0181	iTRAQ4plex@N-term; iTRAQ4plex(K)@3	99	1586.83	94.21	30	123.5	34.64	14.65	14.49	8.92	9.49	474	473	489
KLPPGEYILVPST	3	568.0066	568.0058	0.0023	iTRAQ4plex@N-term; iTRAQ4plex(K)@1	99	12366.31	2962.89	167.73	3063.41	173.35	116.35	54.03	175.26	41.23	476	475	488
KLPPGEYILVPSTF	3	617.0317	617.0286	0.0091	iTRAQ4plex@N-term; iTRAQ4plex(K)@1	99	5708.46	1358.57	113.65	1493.9	120.77	0	29.42	46.19	20.97	476	475	489
KLPPGEYILVPSTFEPNK	4	616.1112	616.1071	0.0164	iTRAQ4plex@N-term; iTRAQ4plex(K)@1; iTRAQ4plex(K)@18	99	506.54	97.64	30.65	164.68	39.74	7.29	13.04	21.1	14.14	476	475	493
FEPNKDGDFCIRVF	3	674.3385	674.3336	0.0147	iTRAQ4plex@N-term; iTRAQ4plex(K)@5; Methylthio(C)@10	99	3682.38	1039.45	99.28	1018.11	99.99	0	38.91	12.52	10.49	489	488	502
EPNKDGDFCIRVF	3	625.3151	625.3108	0.0129	iTRAQ4plex@N-term; iTRAQ4plex(K)@4; Methylthio(C)@9	99	1452.65	926.6	93.9	1059.99	101.62	0	24.55	9.76	10	490	489	502
DGDFCIRVFSEK	3	583.9602	583.9562	0.0118	iTRAQ4plex@N-term; Methylthio(C)@5; iTRAQ4plex(K)@12	98.5	282.06	189.53	42.54	245.12	48.78	18.38	17.89	22.68	14.83	494	493	505
GAEEVESNGSLQK	3	545.9539	545.9525	0.0044	iTRAQ4plex@N-term; iTRAQ4plex(K)@13	99	1137.74	477.53	67.44	568.81	74.49	55.47	30	148.35	37.41	268	267	280
AEEVESNGSLQK	3	526.9476	526.9453	0.0068	iTRAQ4plex@N-term; iTRAQ4plex(K)@12	99	436.61	645.2	78.22	630.79	79.17	218.04	49.49	172.94	41.23	269	268	280

Peptides were extracted from the distinct peptide list generated with ProteinPilot software by the following conditions; Accessions, P17655_CAN2_HUMAN+ "Best conf (Peptide)" > 96.8 (FDR < 1%); permitted modifications, iTRAQ at N-terminus and/or Lys, oxidation at Met, methylthio at Cys, PyroGlu at N-termimus; omit decoy sequence; quant annotation, Auto or Auto--shared MS/MS. The cleavage sites are indicated by the number of amino acid position N-terminus to the cleavage site.

Table S4 List of catalytic subunit of calpain-2 peptide generated by calpain-1.

Sequence	Theor z	Obs m/z	Theor m/z	dMass	Modifications	Best Conf (Peptide)	Intensity (Peptide)	Start Position	cleavage sites N site	cleavage sites C site
AGIAAKLAKDRE	3	414.9066	414.910339	-0.0112571		99	2464.38	2	1	13
AGIAAKLAKDREA	2	657.3751	657.380432	-0.0106074		99	214.29	2	1	14
AGIAAKLAKDREAA	3	462.2634	462.268402	-0.0150501		97.1	162.14	2	1	15
QDYEALRNECLE	2	733.3085	733.316834	-1.67E-02	Gln->pyro-Glu@N-term	99	141.54	30	29	41
ALRNECLE	2	474.2309	474.234375	-0.0069766		96.6	642.75	34	33	41
ADPQFIIGGATR	2	623.3245	623.332947	-0.0168406		99	197.22	83	82	94
SVTGAEEVESNGSLQK	2	817.8907	817.897034	-0.0126896		99	179.59	265	264	280
GAEEVESNGSLQK	2	674.316	674.322998	-0.0139829		99	1737.13	268	267	280
GAEEVESNGSLQKLI	2	787.4117	787.407044	0.00929541		94.6	90.65504	268	267	282
AEEVESNGSLQK	2	645.8053	645.812256	-0.0138815		99	509.35	269	268	280
SDTYKKWKLTK	3	466.5935	466.597748	-1.28E-02		98.5	185.01	350	349	360
EVPEELSGQTNIH	2	726.8458	726.851868	-1.21E-02		99	332.2	435	434	447
RARERSDTFINLR	4	409.2228	409.227478	-0.0187056		99	622.3	457	456	469
SEKKADYQAVDDEIE	3	580.5996	580.603882	-0.0127404		99	358.77	503	502	517

Peptides were extracted from the distinct peptide list generated with ProteinPilot software by the following conditions; Accessions, P17655_CAN2_HUMAN; "Best conf (Peptide)" > 93.7 (FDR < 1%); permitted modifications, oxidation at Met, methylthio at Cys, PyroGlu at N-termimus; omit decoy sequence. The cleavage sites are indicated by the number of amino acid position N-terminus to the cleavage site.

Table S5 List of catalytic subunit of calpain-2 peptide generated by calpain-2.

C2-CS/ CAPN2 cleavage sites

Sequence	Theor z	Obs m/z	Theor m/z	dMass	Modifications	Best Conf (Peptide)	Intensity (Peptide)	Start Position	N site	C site
AGIAAKLAKDRE	3	414.9066	414.910339	-0.0112571		99	8548.28	2	1	13
AGIAAKLAKDREA	2	657.377	657.380432	-0.0068232		99	475.89	2	1	14
AGIAAKLAKDREAAEG	3	524.2845	524.289795	-1.58E-02		99	395.14	2	1	17
AGIAAKLAKDREAAEGLG	3	580.9879	580.991638	-0.0110812		99	2455.7	2	1	19
LAKDREAAEGLG	2	615.3237	615.327881	-0.0082568		99	385.88	8	7	19
QDYEALRNECLE	2	733.3146	733.316834	-0.0045113	Gln->pyro-Glu@N-term	99	2231.57	30	29	41
QDYEALRNECLE	2	741.8257	741.830078	-0.0088436		99	158.8104	30	29	41
YEALRNECLE	2	620.2841	620.287354	-6.45E-03		98.4	1480.94	32	31	41
LGFKELGPYS	2	555.7902	555.795105	-0.0098196		97.5	145.09	58	57	67
ADPQFIIGGATR	2	623.3278	623.332947	-0.0102488		99	945.59	83	82	94
NEEILARVVPLN*	2	683.8857	683.888062	-0.0048049		99	348.93	117	116	129
EEILARVVPLN*	2	626.8638	626.866638	-0.0057007		99	347.75	118	117	129
ADSEAITFQK	2	555.2729	555.277283	-0.0088303		99	194.49	248	247	257
ADSEAITFQKLV	2	661.3483	661.353516	-0.0104293		99	5.191046	248	247	259
QKLVKGHAY	2	513.7865	513.790161	-7.37E-03	Gln->pyro-Glu@N-term	99	153.29	256	255	264
QKLVKGHAYSVT	2	657.3591	657.364258	-0.0103491	Gln->pyro-Glu@N-term	99	685.6	256	255	267
QKLVKGHAYSVTG	3	457.5812	457.585754	-0.0136071	Gln->pyro-Glu@N-term	97.2	1019.28	256	255	268
LVKGHAYSVT	2	537.7962	537.80072	-0.0090901		99	988.86	258	257	267
KGHAYSVTGAEEVESNG	3	578.9306	578.935852	-0.0156771		99	488.12	260	259	276
KGHAYSVTGAEEVESNGSLQK	4	548.5161	548.521118	-1.99E-02		99	397.47	260	259	280
GHAYSVTGAEEVE	2	674.7999	674.804443	-0.0089736		99	777.84	261	260	273
GHAYSVTGAEEVESNG	2	803.8486	803.8526	-0.0081031		99	733.41	261	260	276
GHAYSVTGAEEVESNGSLQK	3	688.3229	688.327393	-0.0135282		99	993.59	261	260	280
YSVTGAEEVESNG	2	671.2895	671.293884	-0.0087778		99	398.87	264	263	276

YSVTGAEEVESNGSLQK	2	899.4255	899.428711	-0.0064381		99	673.75	264	263	280
GAEEVESNGSLQ	2	610.2709	610.275513	-0.0092298		99	291.49	268	267	279
GAEEVESNGSLQK	2	674.3188	674.322998	-0.0083676		99	7915.38	268	267	280
AEEVESNGSLQK	2	645.8059	645.812256	-0.0126608		99	2992.52	269	268	280
AEEVESNGSLQKLI	2	758.8945	758.896301	-0.0036647		99	32.00719	269	268	282
NTIDPEERERLT	2	736.8654	736.870606	-0.0104033		95.5	796.46	305	304	316
SDTYKKWKLTK	3	466.5936	466.597748	-0.0124879		99	4211.74	350	349	360
MDGNWRRGSTAGGCR	4	406.6821	406.686127	-1.61E-02		98.7	3865.3	361	360	375
GSTAGGCRNYPNTF	2	722.8145	722.817322	-0.0056364		99	447.72	368	367	381
STAGGCRNYPNTF	2	694.3031	694.30658	-0.0069998		99	7062.97	369	368	381
TAGGCRNYPNTF	2	650.7864	650.790588	-0.0084187		97.8	780.71	370	369	381
AGGCRNYPNTF	2	600.2626	600.266724	-0.0083476		99	1353.15	371	370	381
GCRNYPNTF	2	536.2333	536.237488	-0.0083639		97	7899.22	373	372	381
NYPNTFWMNPQY	2	795.8376	795.837708	-0.000296	Oxidation(M)@8	98.3	183.89	376	375	387
WMNPQYLIKLE	2	725.869	725.873596	-0.0091382	Oxidation(M)@2	98.5	119.07	382	381	392
EVPEELSGQTNIH	2	726.8472	726.851868	-0.0093321		99	3385.67	435	434	447
EVPEELSGQTNIHLS	2	826.9072	826.909912	-5.43E-03		99	569.44	435	434	449
EVPEELSGQTNIHLSK	3	594.3038	594.307373	-1.08E-02		99	718.55	435	434	450
VPEELSGQTNIH	2	662.327	662.330627	-0.0071443		99	125.05	436	435	447
ELSGQTNIHLS	2	599.8017	599.806763	-0.010125		96.3	584.28	439	438	449
NIHLSKNFFLTN	2	724.3888	724.388245	0.00108632		99	198.06	445	444	456
RARERSDTFINLR	4	409.2236	409.227478	-0.0155317		99	29740.79	457	456	469
ARERSDTFINLR	3	493.2626	493.267181	-0.0137421		98.6	708.2	458	457	469
RERSDTFINLR	3	469.5834	469.588135	-0.014226		99	746.42	459	458	469
KLPPGEYILVPST	2	707.4008	707.403015	-0.0044346		98.3	1799.97	476	475	488
KLPPGEYILVPSTFEPNK	3	677.0349	677.03833	-0.0102718		99	810.62	476	475	493
EYILVPSTFEPNK	2	768.8992	768.900879	-0.0033734		99	339.14	481	480	493
DGDFCIRVF	2	536.2465	536.250061	-0.0070267		99	1231.63	494	493	502
SEKKADYQAVDDEIE	3	580.6001	580.603882	-0.0112755		95.7	625.41	503	502	517

EDAEISAFELQ	2	626.2889	626.290588	-3.46E-03		99	200.68	544	543	554
REIDVDRSGTMNS	3	499.2271	499.23169	-0.0137635	Oxidation(M)@11	97.7	708.66	612	611	624
REIDVDRSGTMNSY	3	553.5823	553.586121	-0.0116013	Oxidation(M)@11	99	347.3	612	611	625
NSYEMRKALE	2	620.7983	620.803162	-0.0097671		99	151.87	623	622	632

Peptides were extracted from the distinct peptide list generated with ProteinPilot software by the following conditions; Accessions, P17655_CAN2_HUMAN; "Best conf (Peptide)" > 94.1 (FDR < 1%); permitted modifications, oxidation at Met, methylthio at Cys, PyroGlu at N-termimus; omit decoy sequence. The cleavage sites were described with number of amino acid position of N-terminus of the cleavage site. Bold letter, product of CuT cleavage. *, Peptides produced by cleavage at least one of the CuT sites.

Table S6 List of peptide of calpain small subunit generated by C2.

								Intensity of iTRAQ						cleavage sites	
Sequence	Theor z	Obs m/z	Theor m/z	dMass	Modifications	Best Conf (Peptide)	Intensity (Peptide)	C2 Area 114	Err 114	C1:CS+C2 Area 115	Err 115	(C1:CS/C2) 115/114	Start Position	N site	C site
GNVLGGLISG	2	515.8048	515.804321	0.0010	iTRAQ4plex@N-term	99	4736.72	437.34	64.33	383.18	61.8	0.8762	26	25	35
GNVLGGLIS	2	487.294	487.293579	0.0009	iTRAQ4plex@N-term	93.6	5535.94	92.84	30	186.99	42.42	2.0141	26	25	34
GNVLGGLISGAGGG	2	636.8575	636.855042	0.0049	iTRAQ4plex@N-term	99	4.49E+02	83.72	28.28	110.88	33.16	1.3244	26	25	39
GNVLGGLISGAGGGGGG	2	722.3922	722.387268	0.0099	iTRAQ4plex@N-term	99	457.2	51.18	22.36	122.41	34.64	2.3918	26	25	42
GNVLGGLISGAGGGGGGGGGGGGGGGGGGGTAMRI	3	1000.842	1000.83105	0.0335	iTRAQ4plex@N-term; Oxidation(M)@34	99	4045.49	290.47	52.15	106.26	33.91	0.3658	26	25	63
GNVLGGLISGAGGGG	2	665.3687	665.365784	0.0059	iTRAQ4plex@N-term	99	368.01	52.02	22.36	83.16	28.28	1.5986	26	25	40
GNVLGGLISGAGGGGGGGGGGGGGGGGGGGTAM	3	854.4099	854.400818	0.0273	iTRAQ4plex@N-term; Oxidation(M)@34	99	445.47	52.03	22.36	82.81	28.28	1.5916	26	25	59
GNVLGGLISGAGGGGG	2	693.8796	693.876526	0.0061	iTRAQ4plex@N-term	99	430.89	73.83	26.45	70.74	26.45	0.9581	26	25	41
GLISGAGGGGGGGGGGGGGGGGGGGTAMRILG	3	854.0919	854.084961	0.0209	iTRAQ4plex@N-term; Oxidation(M)@29	99	2101.57	113.45	33.16	229.81	46.9	2.0257	31	30	63
GLISGAGGGGGGGGGGGGGGGGGGGTAMRILG	3	848.7609	848.753296	0.0229	iTRAQ4plex@N-term	99	2828.63	108.27	32.4	220.34	45.82	2.0351	31	30	63
GLISGAGGGGGGGGGGGGGGGGGGGTAMRILGGVIS	3	972.8314	972.820251	0.0335	iTRAQ4plex@N-term; Oxidation(M)@29	99	2181.53	165.2	39.36	75.75	28.28	0.4585	31	30	67
GAGGGGGGGGGGGGGGGGGGGTAMRILGGVIS	3	844.0885	844.081421	0.0212	iTRAQ4plex@N-term	98.3	431.94	68.56	25.49	65.65	25.49	1.3285	35	34	67
RILGGVISAISE	2	679.9129	679.909851	0.0062	iTRAQ4plex@N-term	99	4494.52	71.87	26.45	161.37	39.36	2.2453	58	57	69
GGVISAISEAAA	2	595.3326	595.330872	0.0034	iTRAQ4plex@N-term	99	6077.85	207.84	44.71	344.64	57.61	1.6582	61	60	72
GGVISAISE	2	488.7753	488.775208	0.0002	iTRAQ4plex@N-term	99	3342.44	324.02	55.67	447.74	66.55	1.3818	61	60	69
GGVISAISEAAAQ	2	659.364	659.360168	0.0077	iTRAQ4plex@N-term	99	3293.22	115.87	33.16	117.21	34.78	1.0116	61	60	73
GGVISAISEAA	2	559.8141	559.812317	0.0035	iTRAQ4plex@N-term	96.7	1097.93	61.82	24.49	128.39	35.35	1.1608	61	60	71
GVISAISEAAA	2	566.8226	566.820129	0.0049	iTRAQ4plex@N-term	99	46522.62	1889.21	134.78	3097.11	173.84	1.6394	62	61	72
GVISAISEAAAQ	2	630.8527	630.849426	0.0066	iTRAQ4plex@N-term	99	38860.03	746.97	84.31	851.75	94.22	1.1403	62	61	73
GVISAISEAAAQY	2	712.3846	712.381104	0.0070	iTRAQ4plex@N-term	99	15034.59	247.36	48.47	258.8	51.57	1.0462	62	61	74
GVISAISEAAAQYNPEPPPPRTH	4	637.3359	637.333435	0.0097	iTRAQ4plex@N-term	99	4200.14	217.92	45.38	176.52	42.42	0.8100	62	61	84
GVISAISEAA	2	531.3023	531.301575	0.0014	iTRAQ4plex@N-term	99	12039.59	387.17	60.98	616.21	78.41	1.5916	62	61	71
SAISEAAAQYNPEPPPPRTH	4	570.0421	570.039978	0.0086	iTRAQ4plex@N-term	99	6206.82	600.3	75.75	816.5	89.26	1.3602	65	64	84
SAISEAAAQY	2	577.7958	577.794128	0.0033	iTRAQ4plex@N-term	99	2181.22	218.14	45.93	416.45	63.47	1.9091	65	64	74
SAISEAAAQYNPEPPPPRTHYSNIE	4	721.6119	721.606201	0.0227	iTRAQ4plex@N-term	99	2592.05	178.28	41.23	217.76	46.36	1.2214	65	64	89
SAISEAAAQYNP	2	683.3455	683.34198	0.0071	iTRAQ4plex@N-term	99	215.58	62.83	24.49	81.06	28.28	1.2901	65	64	76
SAISEAAAQYNPEPPPPR	3	680.3525	680.348633	0.0115	iTRAQ4plex@N-term	99	724.08	84.43	28.28	77.92	28.28	0.9229	65	64	82
AISEAAAQYNPEPPPPRTHYSNIE	4	699.8536	699.848206	0.0215	iTRAQ4plex@N-term	99	28308.37	1741.62	128.12	1200.24	112.59	0.6892	66	65	89
AISEAAAQYNPEPPPPRTHY	3	785.067	785.061279	0.0172	iTRAQ4plex@N-term	99	6567.16	403.38	62.2	613.58	77.19	1.5211	66	65	85
AISEAAAQYNPEPPPPR	3	651.3402	651.338013	0.0067	iTRAQ4plex@N-term	99	8048.93	392	60.98	389.42	64.26	0.9934	66	65	82
AISEAAAQYN	2	591.3002	591.299561	0.0012	iTRAQ4plex@N-term	99	431.14	158.07	38.72	156.88	39.99	0.9925	66	65	75
AISEAAAQYNPEPPPPRT	3	685.0244	685.020569	0.0116	iTRAQ4plex@N-term	99	851.74	78.63	27.38	97.18	31.62	1.2359	66	65	83
AISEAAAQYNPEPPPPRTH	4	548.2839	548.281982	0.0077	iTRAQ4plex@N-term	99	52430.34	3588.3	185.18	4824.9	218.6	1.3446	66	65	84
AISEAAAQYNP	2	639.8285	639.825989	0.0051	iTRAQ4plex@N-term	99	2126.86	336.58	56.74	463.91	67.37	1.3783	66	65	76
AISEAAAQY	2	534.2795	534.278137	0.0027	iTRAQ4plex@N-term	84.6	13888.22	1006.52	98.27	1546.48	124.52	0.8172	66	65	74
SEAAAQYNPEPPPPRTH	3	669.3361	669.33313	0.0090	iTRAQ4plex@N-term	99	3567.48	103.88	31.62	174.25	40.98	1.6774	68	67	84
SEAAAQYNPEPPPPRTHYSNIE	4	653.8213	653.817871	0.0136	iTRAQ4plex@N-term	99	929.99	90.4	29.15	50.57	23.45	0.5594	68	67	89

EAAAQYNPEPPPPRTH	3	640.3251	640.322449	0.0079	iTRAQ4plex@N-term	99	45016.88	2049.47	140.8	3953.21	198.44	1.9289	69	68	84
EAAAQYNPEPPPPRTHY	3	694.6813	694.67688	0.0132	iTRAQ4plex@N-term	99	4681.07	312.11	54.86	550.93	73.2	1.7652	69	68	85
EAAAQYNPEPPPPR	3	560.9557	560.953552	0.0063	iTRAQ4plex@N-term	99	5091.77	273.44	51.08	348.84	61.96	1.2757	69	68	82
EAAAQYNPEPPPPRT	3	594.6382	594.636169	0.0061	iTRAQ4plex@N-term	99	502.5	63.37	24.49	56.32	24.49	1.1048	69	68	83
EAAAQYNPEPPPPRTHYSNIE	4	632.0628	632.059875	0.0117	iTRAQ4plex@N-term	99	13730.26	981.98	96.32	785.28	93.15	1.2828	69	68	89
AAAQYNPEPPPPRTHY	3	651.6649	651.66272	0.0067	iTRAQ4plex@N-term	99	6510.52	708.47	82.21	890.49	93.42	1.2569	70	69	85
AAAQYNPEPPPPR	3	517.9394	517.939392	0.0000	iTRAQ4plex@N-term	99	2363.39	462.06	66.4	584.87	76.47	1.2658	70	69	82
AAAQYNPEPPPPRTH	3	597.3105	597.308228	0.0067	iTRAQ4plex@N-term	99	68535.54	5895.74	238.25	10038.05	312.49	1.7026	70	69	84
AAAQYNPEPPPPRTHYSNIE	4	599.8018	599.799255	0.0103	iTRAQ4plex@N-term	99	27416.76	3313.51	176.67	2188.58	152.56	2.5575	70	69	89
AAQYNPEPPPPRTH	3	573.6305	573.629211	0.0038	iTRAQ4plex@N-term	99	11294.81	727.21	84.55	1966.99	138.07	2.7048	71	70	84
AAQYNPEPPPPRTHY	3	627.986	627.983643	0.0071	iTRAQ4plex@N-term	99	853.37	105.18	31.62	113.88	33.91	1.0827	71	70	85
AAQYNPEPPPPRTHYSNIE	3	775.7231	775.717529	0.0167	iTRAQ4plex@N-term	99	4232.98	246.69	48.37	241.37	50.49	1.7006	71	70	89
AQYNPEPPPPRTH	3	549.951	549.950195	0.0024	iTRAQ4plex@N-term	99	31981.35	2555.15	157.49	5365.05	230.75	2.0997	72	71	84
AQYNPEPPPPR	3	470.5818	470.581299	0.0014	iTRAQ4plex@N-term	99	1376.9	479.6	68.11	920.14	95.28	1.9186	72	71	82
AQYNPEPPPPRTHYSNIE	3	752.0434	752.038513	0.0147	iTRAQ4plex@N-term	99	1.34E+04	805.87	87.51	868.16	96.84	1.0773	72	71	89
AQYNPEPPPPRTHY	3	604.3071	604.304626	0.0075	iTRAQ4plex@N-term	99	2999.97	272.23	50.98	353.24	59.41	1.2976	72	71	85
QYNPEPPPPRTHY	3	580.6279	580.625549	0.0071	iTRAQ4plex@N-term	99	4579.42	769.57	86.07	1299.14	112.59	1.6881	73	72	85
QYNPEPPPPRTHYSNIE	3	728.3635	728.359436	0.0122	iTRAQ4plex@N-term	99	23990.46	2356.64	148.81	1285.01	124.28	0.5453	73	72	89
QYNPEPPPPRTH	3	526.2719	526.271118	0.0023	iTRAQ4plex@N-term	99	54199.75	2963.05	168.85	4955.74	222	1.6725	73	72	84
YNPEPPPPRTHY	3	537.9401	537.939392	0.0022	iTRAQ4plex@N-term	99	5981.46	867.48	90.93	1056.43	102.89	1.2178	74	73	85
NPEPPPPRTHYSNIE	3	631.3216	631.318787	0.0083	iTRAQ4plex@N-term	99	29642.94	1060.84	100.14	874.6	98.63	0.8244	75	74	89
NPEPPPPRTH	3	429.2299	429.230499	-0.0018	iTRAQ4plex@N-term	98.8	37432.26	1116.59	103.28	1483.85	122.82	1.3289	75	74	84
YSNIEANESEEVR	3	561.9373	561.936279	0.0032	iTRAQ4plex@N-term	99	3043.63	535.7	71.4	616.59	78.22	1.1510	85	84	97
SNIEANESEEVR	2	760.8741	760.86908	0.0100	iTRAQ4plex@N-term	99	6591.9	1152.58	105.1	1711.75	128.86	1.4851	86	85	97
IEANESEEVR	2	660.3355	660.331604	0.0078	iTRAQ4plex@N-term	99	2213.1	272.42	50.98	344.56	60.16	1.2648	88	87	97
AQLAGDDMEVSATELM	2	912.9361	912.927429	0.0173	iTRAQ4plex@N-term	99	6465.81	249.69	49.08	452.19	66.32	1.8110	104	103	119
AQLAGDDMEVSATELM	2	920.9344	920.924866	0.0190	iTRAQ4plex@N-term; Oxidation(M)@16	99	4557.59	220.74	45.82	245.35	49.59	1.1115	104	103	119
AQLAGDDMEVSATELM	2	920.9333	920.924866	0.0168	iTRAQ4plex@N-term; Oxidation(M)@8	99	3862.25	168.21	39.99	185.84	43.01	1.1048	104	103	119
AQLAGDDMEVSATELM	2	928.9322	928.922363	0.0197	iTRAQ4plex@N-term; Oxidation(M)@8; Oxidation(M)@16	99	1453.12	73.14	26.45	102.47	31.62	1.4010	104	103	119
AQLAGDDMEVSA	2	675.8231	675.820007	0.0062	iTRAQ4plex@N-term	99	2173.6	87.22	29.15	198.38	44.71	1.4010	104	103	115
AQLAGDDMEVS	2	640.3039	640.301453	0.0049	iTRAQ4plex@N-term	89.8	5.80E+02	146.81	37.41	179.68	42.42	1.0925	104	103	114
NILNKVVTRHPDLK	5	416.6607	416.661072	-0.0018	iTRAQ4plex@N-term; iTRAQ4plex(K)@5; iTRAQ4plex(K)@14	94.9	1920.33	474.41	67.22	563.93	75.75	1.5667	120	119	133
KVVTRHPDLK	4	407.0104	407.011017	-0.0025	iTRAQ4plex@N-term; iTRAQ4plex(K)@1; iTRAQ4plex(K)@10	99	4932.73	1305	111.65	1733.74	131.43	1.3285	124	123	133
RHPDLKTDGFGIDTCR	4	542.0283	542.026306	0.0081	iTRAQ4plex@N-term; iTRAQ4plex(K)@6; Methylthio(C)@15	99	1388.45	296.16	53.1	343.79	58.47	1.1608	128	127	143
TDGFGIDTCR	2	637.788	637.784912	0.0062	iTRAQ4plex@N-term; Methylthio(C)@9	99	4935.2	538.35	71.68	690.61	85.54	1.2828	134	133	143
TDGFGIDTC	2	559.7353	559.734375	0.0018	iTRAQ4plex@N-term; Methylthio(C)@9	99	2636.04	119.4	33.91	203.05	46.9	1.7006	134	133	142

SMVAVMDSDTTGKLG	3	600.6454	600.642212	0.0095	iTRAQ4plex@N-term; iTRAQ4plex(K)@13	99	2729.55	265.54	50.49	416.01	63.87	1.5667	144	143	158
SMVAVMDSDTTGKLG	3	605.9765	605.973877	0.0079	iTRAQ4plex@N-term; Oxidation(M)@2; iTRAQ4plex(K)@13	99	688.91	223.41	45.93	171.55	42.42	0.7679	144	143	158
SMVAVMDSDTTGKLG	3	605.977	605.973877	0.0094	iTRAQ4plex@N-term; Oxidation(M)@6; iTRAQ4plex(K)@13	99	473.09	222.21	45.93	226.96	48.06	1.0214	144	143	158
VAVMDSDTTGKLG	3	527.9521	527.951416	0.0021	iTRAQ4plex@N-term; iTRAQ4plex(K)@11	99	1816.49	646.72	78.41	706.57	85.25	1.0925	146	145	158
KQFDTDRSGTICS	3	597.9609	597.958435	0.0073	iTRAQ4plex@N-term; iTRAQ4plex(K)@1; Methylthio(C)@12	99	6056.26	1766.6	130.06	2539.99	156.76	0.7679	179	178	191
SELPGAFEAAGFHLNEH	3	657.3259	657.322327	0.0108	iTRAQ4plex@N-term	99	290.65	84.63	28.28	69.16	26.45	0.8172	190	189	206
EAAGFHLNEHLY	3	515.5927	515.59198	0.0022	iTRAQ4plex@N-term	99	3867.03	147.94	38.07	378.35	60.98	2.5575	197	196	208

Peptides were extracted from the distinct peptide list generated with ProteinPilot software by the following conditions; Accessions, P04632|CPNS1_HUMAN; "Best conf (Peptide)" > 81.6 (FDR < 1%); permitted modifications, iTRAQ at N-terminus and/or Lys, oxidation at Met, methylthio at Cys, PyroGlu at N-termimus; permitted quant annotation, Auto and Auto--shared MS/MS with intensity higher than 50; start position, > 25. The cleavage sites are indicated by the number of amino acid position N-terminus to the cleavage site.

Table S7 List of C2 specific peptide in PC domain generated by C2 in the presence and absence of C1:CS.

Sequence	Theor z	Obs m/z	Theor m/z	dMass	Modifications	Best Conf (Peptide)	Intensity (Peptide)	Intensity of iTRAQ C2 Area 114	Err 114	C1:CS+C2 Area 115	Err 115	(C1:CS/C2) 115/114	Start Position	cleavage sites N site	C site
YEALRNECLE	2	715.3372	715.332214	0.00996	iTRAQ4plex@N-term; Methylthio(C)@8	99	3837.29	890.56	92.07	1034.12	100.43	1.161202	32	31	41
ALRNECLEAGTLF	3	542.9427	542.941528	0.0035	iTRAQ4plex@N-term; Methylthio(C)@6	99	1569.16	137.93	36.05	93.25	30.82	0.6760676	34	33	46
NECLEAGTLF	2	643.8008	643.797485	0.00657	iTRAQ4plex@N-term; Methylthio(C)@3	99	21449.41	2579.44	156.57	2780.32	164.81	1.077877	37	36	46
CLEAGTLF	2	522.2553	522.254761	1.14E-03	iTRAQ4plex@N-term; Methylthio(C)@1	98.8	362.62	108.65	32.09	102.41	31.78	0.9425679	39	38	46
QDPSFPAIPSALGF	2	795.9238	795.917847	0.01196	iTRAQ4plex@N-term	99	6486.7	605.71	75.55	414.55	64.64	0.6844034	47	46	60
QDPSFPAIPSAL	2	693.875	693.872925	0.00418	iTRAQ4plex@N-term	99	925.33	113.49	32.86	127.69	35.35	1.125121	47	46	58
QDPSFPAIPSA	2	637.3348	637.330872	0.0078	iTRAQ4plex@N-term	99	6183.22	891.74	92.24	1129.36	105.06	1.266468	47	46	57
QDPSFPAIPSALG	2	722.3873	722.383667	0.00738	iTRAQ4plex@N-term	99	4798.67	444.07	65.03	520.49	71.19	1.17209	47	46	59
EEILARVVPLNQ	3	621.6841	621.679993	0.01225	iTRAQ4plex@N-term; iTRAQ4plex(K)@15	99	766.96	265.71	50.19	257.84	50.49	0.9703813	47	46	58
QDPSFPAIPSALGFK	3	702.3926	702.388855	1.11E-02	iTRAQ4plex@N-term; iTRAQ4plex(K)@15	99	3146.49	477.59	66.92	216.18	47.74	0.4526477	47	46	61
QDPSFPAIPSALGFKEL	3	837.1195	837.112061	0.02228	iTRAQ4plex@N-term; iTRAQ4plex(K)@15	99	1151.55	368.69	58.64	72.96	29.83	0.1978898	47	46	63
QDPSFPAIPSALGFKELGPYS	3	695.3885	695.383972	0.00903	iTRAQ4plex@N-term	99	1559.4	434.3	64.18	424.6	64.72	0.9776652	83	82	103
CSIDITSA	2	623.3119	623.310303	0.00485	iTRAQ4plex@N-term; Methylthio(C)@16	99	468.82	99.54	30.65	75.71	27.56	0.7605987	83	82	90
ADPQFIIGGATR	2	665.9995	665.99646	0.00914	iTRAQ4plex@N-term; Methylthio(C)@16	99	4565.21	845.72	89.32	611.75	78.41	0.7233482	83	82	94
ADPQFIIGGATRTDIC	3	584.6768	584.676025	0.00231	iTRAQ4plex@N-term	99	422.55	98.4	30.49	79.28	28.28	0.805691	116	115	131
ADPQFIIGGATRTDICQ	3	546.9827	546.981384	4.10E-03	iTRAQ4plex@N-term	99	5280.49	963.74	95.75	1084.81	102.94	1.125625	117	116	133
LNEEILARVVPLNQ	3	504.2952	504.295166	8.34E-05	iTRAQ4plex@N-term	99	5005.4	1025.03	98.93	1332.01	113.61	1.299484	117	116	130
NEEILARVVPLNQ	3	508.9678	508.967041	0.00226	iTRAQ4plex@N-term	99	5113.72	483.46	67.89	590.8	75.82	1.222025	118	117	130
NEEILARVVPLN	3	698.9216	698.917664	7.84E-03	iTRAQ4plex@N-term	99	9617.32	1591.4	123.07	1830.22	133.51	1.150069	118	117	129
EEILARVVPLN	2	724.3567	724.352356	0.0087	iTRAQ4plex@N-term	99	1829.28	157.94	38.72	162.88	39.99	1.031278	129	128	139
QSFQENYAGIF	2	660.3257	660.323059	5.26E-03	iTRAQ4plex@N-term	99	1385.96	178.79	41.23	194.47	43.58	1.087701	130	129	140
SFQENYAGIF	2	555.7979	555.796265	0.00327	iTRAQ4plex@N-term	99	3364.3	442.14	64.88	511.27	70.56	1.156353	136	135	145
AGIFHFQF	2	720.3437	720.339233	0.00891	iTRAQ4plex@N-term	99	676.19	115.54	33.16	132.68	36.05	1.148347	167	166	174
FVHSAEGSEFW	2	646.8085	646.805054	0.00689	iTRAQ4plex@N-term	99	1396.01	252.39	49.08	325.13	56.21	1.288205	168	167	178
VHSAEGSEFW	2	631.7825	631.779297	0.00633	iTRAQ4plex@N-term; Methylthio(C)@1	99	697.2	121	33.91	129.86	36.05	1.073223	191	190	200
CYEALSGGATT	2	518.9536	518.951721	5.68E-03	iTRAQ4plex@N-term; iTRAQ4plex(K)@11	94	288.93	142.99	36.87	158.34	39.36	1.10735	208	207	218
TGGIAEWYELK	3	698.8936	698.889343	0.00849	iTRAQ4plex@N-term; iTRAQ4plex(K)@9	99	1319.25	296.06	52.91	246.34	49.59	0.8320611	210	209	220

GIAEWYELK	2	500.2341	500.234009	1.95E-04	iTRAQ4plex@N-term; Methylthio(C)@1	96.1	2060.67	559.21	73.06	725.34	84.01	1.29708	240	239	248
CSIDITSAADS	2	636.7867	636.782043	9.27E-03	iTRAQ4plex@N-term; Methylthio(C)@1	99	310.41	52.2	22.36	75.26	27.38	1.441762	240	239	250
CSIDITSAADSEAI	2	793.3699	793.363953	0.01188	iTRAQ4plex@N-term; Methylthio(C)@1	99	843.54	57.43	23.45	82.47	28.28	1.436009	240	239	253
CSIDITSAADSEAIT	2	843.8964	843.887756	0.01731	iTRAQ4plex@N-term; Methylthio(C)@1	99	4034.38	293.43	52.43	120.41	36.05	0.4103534	240	239	254
CSIDITSAADSE	2	701.3077	701.303345	0.00867	iTRAQ4plex@N-term; Methylthio(C)@1	99	2912.74	277.72	51.47	350.3	58.39	1.261342	240	239	251
CSIDITSAAD	2	593.2691	593.266052	0.00615	iTRAQ4plex@N-term; Methylthio(C)@1	99	718.64	73.6	26.45	81.09	28.28	1.101766	240	239	249
IDITSAADSEAIT	2	725.8792	725.873291	0.01182	iTRAQ4plex@N-term	99	727	73.28	26.45	96.5	30.82	1.316867	242	241	254
IDITSAADSEAITF	2	799.4129	799.407532	0.01079	iTRAQ4plex@N-term	99	727.29	57.81	23.45	64.27	25.1	1.111745	242	241	255
SAADSEAITF	2	578.2878	578.286133	0.00329	iTRAQ4plex@N-term	99	349.51	51.61	22.36	102.79	31.62	1.991668	246	245	255
ADSEAITFQKLV	3	537.3062	537.306152	0.00015	iTRAQ4plex@N-term; iTRAQ4plex(K)@10	99	728.63	125.75	34.64	158.1	39.36	1.257257	248	247	259
ADSEAITFQKLVK	3	628.0416	628.038513	0.00937	iTRAQ4plex@N-term; iTRAQ4plex(K)@10; iTRAQ4plex(K)@13	99	689.64	238.39	47.53	227.06	47.43	0.9524728	248	247	260
GHAYSVTGAEEVESNGSLQK	4	588.5512	588.548401	0.01128	iTRAQ4plex@N-term; iTRAQ4plex(K)@20	99	1051.4	283.8	51.95	317.69	55.76	1.119415	261	260	280
GHAYSVTGAEEVE	2	746.8579	746.855469	0.00493	iTRAQ4plex@N-term	99	586.73	73.81	26.45	70.92	26.45	0.9608454	261	260	273
GHAYSVTGAEEVESNG	2	875.9103	875.903625	0.01325	iTRAQ4plex@N-term	99	1112.14	63.27	24.49	60.91	24.49	0.9626995	261	260	276
YSVTGAEEVESNG	2	743.3503	743.34491	0.01074	iTRAQ4plex@N-term	99	3123.82	292.62	52.91	406.11	62.6	1.387841	264	263	276
YSVTGAEEVESNGSLQK	3	696.0284	696.022949	1.64E-02	iTRAQ4plex@N-term; iTRAQ4plex(K)@17	99	2602.5	1483.94	118.39	1163.04	107.97	0.7837514	264	263	280
YSVTGAEEVE	2	614.2995	614.296692	0.0056	iTRAQ4plex@N-term	87.4	563.99	148.84	37.41	85.36	30	0.5735018	264	263	273
SVTGAEEVESNG	2	661.8165	661.813232	0.00657	iTRAQ4plex@N-term	99	1683.8	149.6	37.81	200.18	44.15	1.338102	265	264	276
SVTGAEEVESNGSLQK	3	641.6721	641.668518	0.01089	iTRAQ4plex@N-term; iTRAQ4plex(K)@16	99	1916.66	705.15	81.54	496.37	70.98	0.7039211	265	264	280
GAEEVESNGSLQK	3	545.9534	545.952454	0.00295	iTRAQ4plex@N-term; iTRAQ4plex(K)@13	99	21285.25	3548.08	183.66	3890.71	195.01	1.096568	268	267	280
GAEEVESNGSLQKLI	3	621.3462	621.341797	0.0131	iTRAQ4plex@N-term; iTRAQ4plex(K)@13	99	7761.72	999.49	97.4	1023.85	100.19	1.024372	268	267	282
GAEEVESNGSLQ	2	682.3307	682.326538	0.00834	iTRAQ4plex@N-term	99	2207.57	134.96	36.05	230.57	47	1.708432	268	267	279
GAEEVESNG	2	518.2404	518.239197	0.00246	iTRAQ4plex@N-term	99	1741.18	281.11	51.47	192.64	44.15	0.6852834	268	267	276
AEEVESNGSLQ	2	653.8187	653.815796	5.87E-03	iTRAQ4plex@N-term	99	2339.59	179.81	41.35	196.63	44.04	1.093543	269	268	279
AEEVESNGSLQK	3	526.9456	526.945313	0.00097	iTRAQ4plex@N-term; iTRAQ4plex(K)@12	99	17513.49	7454.23	265.95	7503.43	271.4	1.0066	269	268	280
AEEVESNGSLQKLI	3	602.3381	602.334656	0.01021	iTRAQ4plex@N-term; iTRAQ4plex(K)@12	99	3039.28	721.91	82.69	663.5	81.04	0.9190897	269	268	282
CPSWNTIDPEERERLT	3	712.6747	712.670532	0.01246	iTRAQ4plex@N-term; Methylthio(C)@1	99	8194.83	2695.38	160.1	2985.08	170.97	1.10748	301	300	316

CPSWNTIDPEE	2	740.8179	740.813904	0.00799	iTRAQ4plex@N-term; Methylthio(C)@1	99	5948.75	693.28	80.98	596.33	76.87	0.8601575	301	300	311
RRHEDGEFWMSF	3	586.2725	586.270935	4.81E-03	iTRAQ4plex@N-term; Oxidation(M)@10	84.7	2279.83	135.13	36.05	222.46	46.36	1.646267	317	316	328

Peptides were extracted from the distinct peptide list generated with ProteinPilot software by the following conditions; Accessions, P04632|CPNS1_HUMAN; "Best conf (Peptide)" > 81.6 (FDR < 1%); permitted modifications, iTRAQ at N-terminus and/or Lys, oxidation at Met, methylthio at Cys, PyroGlu at N-termimus; permitted quant annotation, Auto and Auto--shared MS/MS with intensity higher than 50; start position, > 25.
The cleavage sites are indicated by the number of amino acid position N-terminus to the cleavage site.

Table S8 List of common peptide among C1 and C2 generated by C2.

Sequence	Theor z	Obs m/z	Theor m/z	dMass	Modifications	Best Conf (Peptide)	Intensity (Peptide)	Intensity of iTRAQ C2 Area 114	Err 114	C1:CS+C2 Area 115	Err 115	(C1:CS/C2) 115/114	Start Position	cleavage sites N site	C site
AAIASLTL	2	452.2855	452.285217	5.64E-04	iTRAQ4plex@N-term	99	1080.82	187	42.42	313.03	54.86	1.673957	109	108	116
AIASLTLN	2	473.7881	473.788116	-6.2E-05	iTRAQ4plex@N-term	99	5309.32	795.69	87.45	1290.96	111.34	1.622441	110	109	117
GATRTDICQ	2	577.7757	577.774353	0.00268	iTRAQ4plex@N-term; Methylthio(C)@8	99	2086.58	249.4	48.78	315.5	55.58	1.265036	91	90	99
AAIASLTLN	2	509.308	509.306671	0.00268	iTRAQ4plex@N-term	99	1387.04	262.25	49.99	318.72	55.76	1.215329	109	108	117

Peptides were extracted from the distinct peptide list generated with ProteinPilot software by the following conditions; Accessions, P04632|CPNS1_HUMAN; "Best conf (Peptide)" > 81.6 (FDR < 1%); permitted modifications, iTRAQ at N-terminus and/or Lys, oxidation at Met, methylthio at Cys, PyroGlu at N-termimus; permitted quant annotation, Auto and Auto--shared MS/MS with intensity higher than 50; start position, > 25. The cleavage sites are indicated by the number of amino acid position N-terminus to the cleavage site.
